# Hybridization and Polyploidy Shaped the Evolutionary History of a Complex of Cryptic Species in European Woodrushes (*Luzula* sect. *Luzula*)

**DOI:** 10.1093/sysbio/syaf065

**Published:** 2025-09-25

**Authors:** Valentin Heimer, Pau Carnicero, Carolina Carrizo García, Andreas Hilpold, Jasna Dolenc Koce, J Luis Leal, Mingai Li, Claudio Varotto, Peter Schönswetter, Božo Frajman

**Affiliations:** Department of Botany, University of Innsbruck, Sternwartestraße 15, 6020 Innsbruck, Austria; Institute for Alpine Environment, Eurac Research, Drususallee 1/Viale Druso 1, 39100, Bozen/Bolzano, Italy; Department of Botany, University of Innsbruck, Sternwartestraße 15, 6020 Innsbruck, Austria; Department of Animal Biology, Plant Biology and Ecology, Autonomous University of Barcelona, 08193 Bellaterra, Spain; Centro Ricerca e Innovazione, Fondazione Edmund Mach, Via Mach 1, 38098 San Michele all’Adige, Italy; Instituto Multidisciplinario de Biología Vegetal (CONICET-UNC), Av. Vélez Sarsfield 1611, 5000 Córdoba, Argentina; Institute for Alpine Environment, Eurac Research, Drususallee 1/Viale Druso 1, 39100, Bozen/Bolzano, Italy; Department of Biology, Biotechnical Faculty, University of Ljubljana, Jamnikarjeva 101, 1000, Ljubljana, Slovenia; Department of Zoology, Stockholm University, Svante Arrheniusväg 18 B, 106 91 Stockholm, Sweden; Centro Ricerca e Innovazione, Fondazione Edmund Mach, Via Mach 1, 38098 San Michele all’Adige, Italy; Centro Ricerca e Innovazione, Fondazione Edmund Mach, Via Mach 1, 38098 San Michele all’Adige, Italy; Department of Botany, University of Innsbruck, Sternwartestraße 15, 6020 Innsbruck, Austria; Department of Botany, University of Innsbruck, Sternwartestraße 15, 6020 Innsbruck, Austria

**Keywords:** allopolyploidy, ddRADseq, Eastern Alps, hybridization, interploidy, polyploid phylogenetics, reticulate evolution

## Abstract

Polyploidization has played a central role in the evolutionary history of most plant lineages, yet it poses significant challenges for phylogenetic inference, particularly in allopolyploid complexes with reticulate species relationships. *Luzula* sect. *Luzula* (Juncaceae) is a taxonomically intricate group characterized by widespread polyploidy, agmatoploidy, and high morphological uniformity. Focusing on the Eastern Alps, a key center of its diversity, we collected 1002 samples of nine species and applied an integrative framework combining ddRADseq, plastid sequencing, relative genome size estimation, and chromosome counting to disentangle its evolutionary history. We extended previously inferred phylogenetic relationships and assessed gene flow among diploids, establishing a baseline for investigating the origin of polyploids. By analyzing patterns of genotype frequencies and genetic affinities to diploids, we inferred the most likely parental species of polyploids and identified key hybridization events shaping the current taxonomic and karyotypic diversity within this group. Our results reveal weak genetic differentiation among some diploid lineages, likely reflecting gene flow and incomplete lineage sorting. We propose a common allopolyploid origin of two tetraploids, which subsequently gave rise to a third tetraploid and a hexaploid species through interploidy hybridization. Although the parental species of some polyploids remain obscure, our genomic data highlight polyploidy and hybridization as major drivers of speciation in this poorly understood lineage. This study underscores the value of integrative approaches in resolving reticulate plant phylogenies and advances our understanding of polyploid speciation.

Polyploidization, or whole genome duplication (WGD), is one of the key drivers of angiosperm diversification (Soltis et al. 2009; Wood et al. 2009). Two common types of polyploidy, auto- and allopolyploidy, are widely recognized (Ramsey and Schemske 2002). Autopolyploids are formed within a single species and possess multiple sets of homologous chromosomes that pair non-preferentially during meiosis, resulting in polysomic inheritance. In contrast, allopolyploids arise from interspecific hybridization and combine divergent parental genomes. During allopolyploid meiosis, chromosomes generally recombine with homologs of the same parental genome rather than with homeologous chromosomes of the other parental genome, leading to disomic inheritance (Lloyd and Bomblies 2016). Between the two extremes of auto- and allopolyploids, WGD can occur along a gradient of divergence between parental genomes, giving rise to intermediate cases such as intervarietal autopolyploids or segmental allopolyploids (Stebbins 1947; Stift et al. 2008; Parisod et al. 2010), and the degree of divergence between subgenomes may change over time due to homoeologous exchange (Edger et al. 2018; Mason and Wendel 2020).

While WGD is associated with a proportional increase in genome size (GS), changes in chromosome number can also occur without changes in GS through chromosome fragmentation or fusion. When these processes affect all chromosomes in a concerted fashion, they are termed agmatoploidy and symploidy, respectively (Malheiros and Gardé 1950; Luceño and Guerra 1996). In contrast to polyploidy, which is widespread in plants (Husband et al. 2013), agmatoploidy and symploidy are restricted to lineages with holocentric (= holokinetic) chromosomes, which are only found in a few plant genera (Melters et al. 2012; Escudero et al. 2016; Zedek and Bureš 2018). Holocentric chromosomes have non-localized centromeres with kinetochore activity along the entire length of the chromatid (Haizel et al. 2005). This enables the successful segregation of the resulting chromosome fragments to daughter nuclei, rendering chromosome fission not necessarily deleterious (Bureš et al. 2013).

*Luzula* (Juncaceae) is one of the few genera where both polyploidy and agmatoploidy/symploidy occur, and it is the only known genus with documented cases of simultaneous fragmentation of entire chromosome sets in different species. Partial agmatoploidy and symploidy, however, have been evidenced in several other plant groups (Guerra 2016). *Luzula* comprises *ca*. 115 species worldwide (Kirschner 2002) and is characterized by high karyotypic diversity with three common chromosome sizes, namely, full-size (AL-type), half-size (BL-type), and quarter-size (CL-type) chromosomes (Nordenskiöld 1951). Its ancestral karyotype is suggested to be 2*n* = 12AL (Bozek et al. 2012). Within the genus, *Luzula* sect. *Luzula* is remarkably diverse and taxonomically challenging, including 57 species worldwide, many of which are morphologically highly similar and thus often misidentified (Kirschner 2002; Bačič et al. 2007a, 2019). In Europe, the Alps in particular represent a center of diversity for *Luzula* sect. *Luzula* and harbor eight species of three ploidy levels (Bačič et al. 2019). At the diploid level, five species with three distinct chromosome sizes have been documented in the Eastern Alps and surrounding regions, the focal area of this study: *Luzula campestris* (2*n* = 12AL) is widespread in low-elevation nutrient-poor meadows; *L. exspectata* (2*n* = 24BL) and *L. divulgatiformis* (2*n* = 24BL), two calcicole species, occur in montane to alpine grasslands and open woodlands at low elevations, respectively (Bačič et al. 2007b); and *L. pallescens* (2*n* = 12AL) is a forest understory species distributed across Eurasia and only reaching the easternmost margin of the Alps. Finally, *L. sudetica* (2*n* = 48CL) is karyologically, morphologically, and ecologically the most distinct, growing in (sub)alpine moist meadows and fens. In addition, three polyploid species occur in the Eastern Alps: the lowland species *L. divulgata* (tetraploid, 2*n* = 24AL) that grows in deciduous woodlands, as well as *L. alpina* (tetraploid, 2*n* = 12AL + 24BL), a partial agmatoploid of alpine grasslands, and *L. multiflora*, which comprises a tetraploid cytotype (2*n* = 24AL) occurring in montane to alpine grasslands and a hexaploid cytotype (2*n* = 36AL) that is widespread in forests and wet meadows (Bačič et al. 2007b).

Hybridization has been evidenced in *Luzula* sect. *Luzula* across and within ploidy levels (Nordenskiöld 1956; Kirschner 1991), possibly hindering phylogenetic inference. Despite several studies aimed at disentangling the evolutionary history of *Luzula* sect. *Luzula* using plastid and internal transcribed spacer (ITS) sequences, the phylogenetic relationships among the species remained unresolved (Drábková et al. 2006; Záveská Drábková and Vlček 2009, 2010; Bozek et al. 2012; Brožová et al. 2022). The higher resolution of double-digest restriction site-associated DNA sequencing (ddRADseq) has recently provided insights into the phylogenetic relationships among diploid *Luzula* species (Carrizo García et al. 2025). However, the species involved in the origin of polyploids remain obscure, even though some hypotheses have been proposed based on morphological or molecular data. Concerning the origin of *L. divulgata*, Kirschner (1992) proposed a close relationship to *L. taurica* (12AL), a diploid species distributed from the Balkan Peninsula to the Caucasus (Kirschner 2002). Furthermore, Kirschner (1992) hypothesized that tetraploid *L. multiflora* might be an autopolyploid of *L. pallescens*, whereas hexaploid *L. multiflora* may be of allopolyploid origin (Kirschner 1996). Finally, an allopolyploid origin of *L. alpina* has been postulated (Kirschner 1992), with *L. exspectata* (24BL) and *L. taurica* (12AL) acting as putative parents (Bačič et al. 2019). Alternatively, *L. alpina* may have originated via fission of half the chromosomes of tetraploid *L. multiflora*, to which it bears strong morphological, ecological, and genetic similarities (Pungaršek et al. 2023).

In this study, we combine ddRADseq with plastid DNA sequencing, relative GS estimation, and chromosome counts to disentangle evolutionary relationships among the European species of *Luzula* sect. *Luzula* and assess long-standing hypotheses regarding the origin of polyploids. Using 1002 samples belonging to nine species with geographic focus on the Eastern Alps and adjacent areas, we (1) extend phylogenetic relationships and infer gene flow among diploids to establish a framework for inferring the origin of polyploids, (2) elucidate the inheritance mode and genetic structure of tetraploids, and finally, (3) identify the most likely progenitor species of the polyploids. Based on previous cytological and phylogenetic studies, we expect to detect signals of gene flow among diploids that contributed to the origin of at least one allopolyploid, *L. alpina*, which we hypothesize to have arisen through hybridization between the agmatoploid *L. exspectata* and a species with full-sized chromosomes. *Luzula divulgata* and both cytotypes of *L. multiflora* may be of either auto- or allopolyploid origin but are expected to have non-agmatoploid ancestors, likely including *L. pallescens*. Due to its smaller CL-type chromosomes, we do not expect *L. sudetica* to be a parental species of any polyploid.

## Materials and Methods

### Plant Material

**Figure 1.**
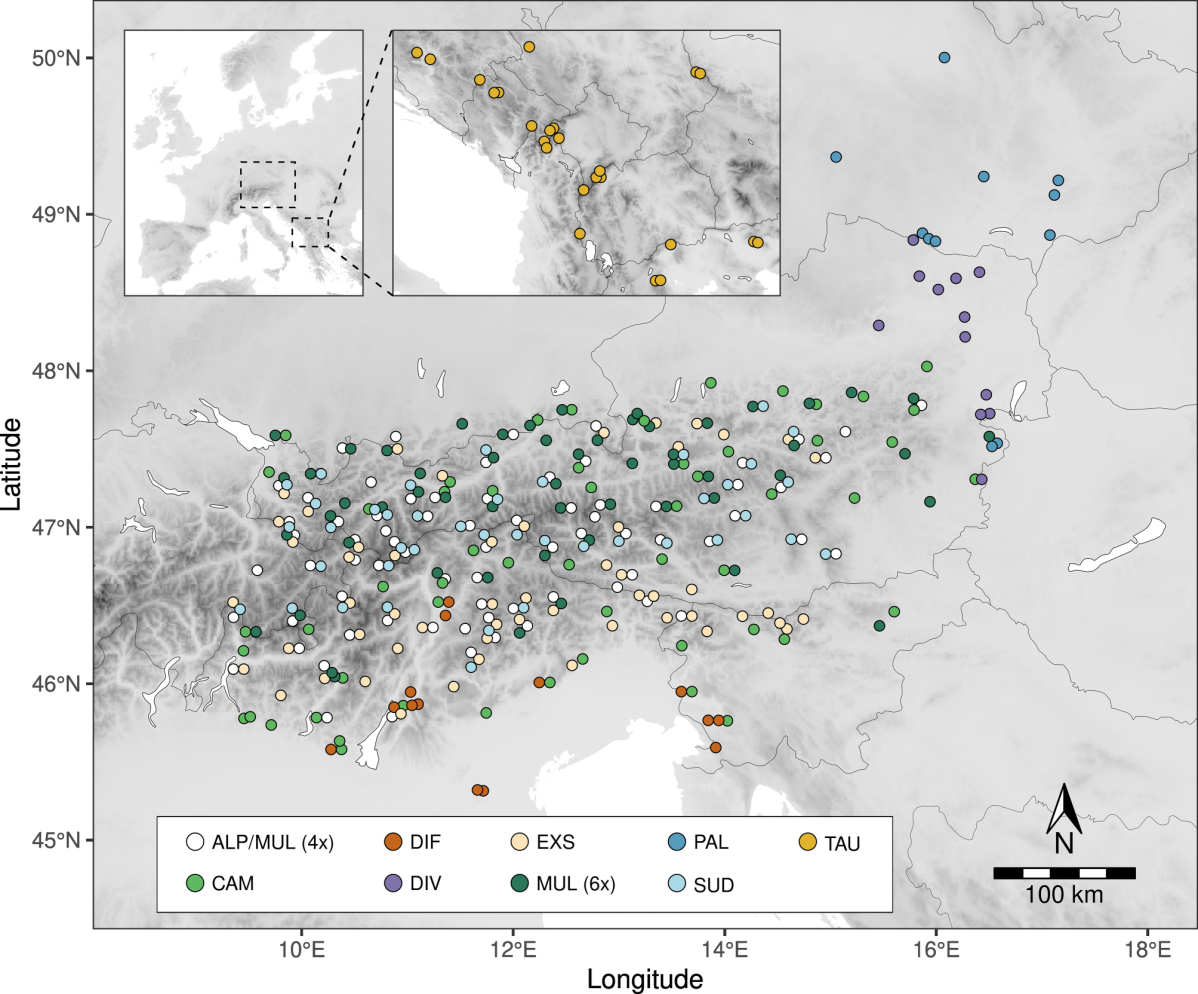
Distribution of 340 *Luzula* sect. *Luzula* populations analyzed in this study. Colors and abbreviations correspond to different species and are consistently used throughout the manuscript. ALP, *L. alpina*; CAM, *L. campestris*; DIF, *L. divulgatiformis*; DIV, *L. divulgata*; EXS, *L. exspectata*; MUL (4×), *L. multiflora* (4*x*); MUL (6×), *L. multiflora* (6*x*); PAL, *L. pallescens*; SUD, *L. sudetica*; TAU, *L. taurica*. Morphologically indistinguishable *L. alpina* and *L. multiflora* (4*x*) were sampled collectively as “alpine tetraploids.” Detailed sampling maps for each species are provided in Supplementary Fig. S1.

We conducted a comprehensive sampling of ten European taxa of *Luzula* sect. *Luzula*, including *L. alpina, L. campestris, L. divulgata, L. divulgatiformis, L. exspectata, L. multiflora* (tetraploid and hexaploid), *L. pallescens, L. sudetica*, and *L. taurica*, across the Eastern Alps and surrounding areas, including the Balkan Peninsula for *L. taurica* (Fig. 1, Supplementary Fig. S1 and Table S1). We sampled between 11 and 73 populations per species (median: 41), amounting to a total of 340 populations. Plants were identified following Bačič et al. (2016, 2019) and Fischer et al. (2008). However, due to the high morphological similarity of *L. alpina* and tetraploid *L. multiflora*, we sampled them collectively as “alpine tetraploids.” Leaf tissue was collected from 1 to 8 (median: 2) individuals per population and was immediately dried in silica gel for DNA extraction and relative GS (RGS) estimation. In addition, we included three individuals of *L. spicata* (*Luzula* sect. *Alpinae*) and two of *L. nivea* (*Luzula* sect. *Anthelaea*) as outgroups (see Supplementary Table S2 for details on sample numbers for each taxon). An herbarium voucher was prepared for each specimen and deposited at IB.

### Relative GS Estimation and Chromosome Counts

The ploidy level of all samples was inferred by measuring the RGS via flow cytometry as described in Suda and Trávníček (2006), using *Bellis perennis* L. (2C = 3.38 pg; Schönswetter et al. 2007) as an internal standard. To accommodate the large number of samples, we also employed high-throughput flow cytometry with a pocketSPU automated sample loading device (Quantum Analysis) for some samples. Nuclei were extracted from silica-dried leaf material by either chopping or, for high-throughput flow cytometry, grinding with glass beads in a tissue lyser (Qiagen), and were stained with 4′,6-diamidino-2-phenylindole (DAPI). Fluorescence of at least 3000 nuclei was measured with a CyFlow space flow cytometer (Sysmex Partec) and analyzed in R v.3.6.3 (R Core Team 2020) using the package *flowPloidy* (Smith et al. 2018). RGS was then calculated as the ratio of mean relative fluorescence between sample and standard.

Because *L. alpina* and tetraploid *L. multiflora* are morphologically extremely similar and cannot be distinguished based on GS (Pungaršek et al. 2023), we generated chromosome counts for a subset of samples. Seeds derived from herbarium vouchers were germinated on filter paper at room temperature in the dark. Root tips were stained with Schiff’s reagent following the protocol described in Bačič et al. (2019), and metaphase chromosomes were counted under an Axiovert 200M microscope (Zeiss) equipped with an AxioCam HRc camera. We complemented our own data with already published chromosome counts from previous studies (Nordenskiöld 1951, 1956; Kirschner et al. 1988; Kirschner 1992; Bačič et al. 2007a, 2016; Pungaršek et al. 2023), and included six samples from Pungaršek et al. (2023).

### DNA Extraction and Plastid DNA Sequencing

Total genomic DNA was extracted from 10 to 20 mg of dried leaf tissue following a modified CTAB protocol (Tel-zur et al. 1999). DNA extracts were subsequently purified using the NucleoSpin gDNA clean-up kit (Macherey-Nagel) and quantified with a Qubit 4 fluorometer (ThermoFisher Scientific).

Because traditional plastid markers such as the *trnL*–*F* region are largely unable to resolve relationships within *Luzula* sect. *Luzula* (Záveská Drábková and Vlček 2010), we identified two variable plastid regions (henceforth referred to as V1 and V5) based on plastid assemblies for nine of our study taxa (Li et al., unpublished). These regions were amplified and sequenced for 405 individuals from 314 populations (Supplementary Table S1). Each region was amplified on Eppendorf 5331 thermocyclers (Applied Biosystems) in a reaction mix (total volume 21 µL) containing 10.2 µL dH_2_O, 7.73 µL REDTaq ReadyMix (Merck), 1.0 µL BSA (1 mg/µL; Promega), 0.53 µl of each primer (10 µM; V1.F and V1.R or V5.F and V5.R; Supplementary Table S3), and 1 µL DNA template. PCR conditions for V1 were 5 min at 95 °C, 35 cycles of 30 s at 95 °C, 30 s at 61 °C and 2 min at 65 °C, followed by 10 min at 65 °C. The same conditions were used for V5, however with a lower annealing temperature of 57.2 °C. After purification of PCR products with *E. coli* Exonuclease I and SAP (Shrimp Alkaline Phosphatase; Fermentas) according to manufacturer’s instructions, Sanger sequencing was performed at Eurofins Genomics (Ebersberg, Germany) using the newly designed primers V1.F, V1.R, and V1.S for V1 and V5.F for V5. Contigs were assembled, aligned, and edited in Geneious Pro v.5.5.9 (Kearse et al. 2012), and both regions were concatenated to a single alignment. Indels were coded as binary characters by applying simple gap coding (Simmons and Ochoterena 2000) in SeqState v.1.25 (Müller 2005).

### 3RAD Library Preparation, Sequencing, Demultiplexing, and Alignment

Double-digest RADseq (ddRADseq) libraries were prepared following a 3RAD protocol adapted from Bayona-Vásquez et al. (2019). Briefly, 100 ng of DNA per sample was digested with the restriction enzymes XbaI and EcoRI-HF (New England Biolabs), and each sample was indexed with a unique combination of EcoRI and XbaI adapters. Digestion and adapter ligation were performed simultaneously, using NheI as a third restriction enzyme to cut adapter-dimers for improved ligation efficiency (Bayona-Vásquez et al. 2019). Following sample pooling, DNA fragments were size-selected for 470–600 bp using SPRIselect magnetic beads (Beckman Coulter) and were subsequently amplified in 14 PCR cycles with Illumina iTru5 and iTru7 primers. After additional size selection on a Pippin Prep (Sage Science) and quality assessment with a High Sensitivity DNA Kit on an Agilent 2100 Bioanalyzer (Agilent Technologies), libraries were sequenced as 150-cycle paired-end reads on an Illumina NovaSeq X Plus platform at Novogene (Munich, Germany). We used FastQC v.0.11.8 (Andrews 2010) for quality assessment of raw Illumina reads, which were then demultiplexed using *process_radtags.pl* implemented in STACKS v.2.62 (Catchen et al. 2013), and restriction sites were trimmed with Trimmomatic v.0.39 (Bolger et al. 2014). Demultiplexed reads were aligned to the reference genome of *L. sylvatica* (Goodwin et al. 2024) using BWA-MEM v.0.7.17 (Li and Durbin 2009) with default settings. The resulting SAM files were converted to BAM format, sorted by reference coordinates, and indexed using samtools v.1.9 (Danecek et al. 2021). Finally, read groups were added in Picard v.2.26.2 (http://broadinstitute.github.io/picard/). Single nucleotide polymorphisms (SNPs) were called using two complementary approaches for diploid and mixed-ploidy data sets in order to maximize resolution while also ensuring accurate SNP calling of polyploids, as described in the following paragraphs. The data sets and filtering applied for each analysis are listed in Supplementary Table S4.

### Diploid Variant Calling and Exploratory Analyses of SNP Data

We first analyzed relationships among diploids to establish a phylogenetic backbone, which was later expanded to include the polyploids. To do this, we created a data set containing only the six diploid species and two diploid outgroup taxa. SNP calling for diploids was performed with STACKS v.2.62 (Catchen et al. 2013). A catalogue was built from indexed BAM files using *refmap.pl* with default settings, and the program *populations* implemented in STACKS was then used to export data in various formats, which were further filtered using BCFtools v.1.20 (Danecek et al. 2021), dependent on subsequent analyses (Supplementary Table S4). We first produced a baseline data set containing all diploid samples and loci present in at least 50% of individuals with a maximum observed heterozygosity (maxH_o_) of 0.65 to construct a NeighborNet phylogenetic network in SplitsTree v.6.3.16 (Huson and Bryant 2024) based on Nei’s distance (Nei 1972) computed in *adegenet* (Jombart and Ahmed 2011).

### Phylogenetic Analyses of Diploids

Phylogenetic relationships among diploids were inferred in IQ-TREE 2 (Minh et al. 2020) based on a data set of concatenated RAD loci present in at least 50% of individuals with a minimum read depth (minDP) of eight, maxH_o_ = 0.65, and a minimum minor allele count (MAC) of 3. The filtered VCF file was converted to PHYLIP format using *vcf2phylip.py* (https://github.com/edgardomortiz/vcf2phylip) and invariant sites were removed in IQ-TREE 2. Finally, a maximum likelihood (ML) tree was constructed with 1000 ultrafast bootstrap replicates (Hoang et al. 2018), ascertainment bias correction, and correction for overestimated node support (-bnni), with the best substitution model automatically determined by ModelFinder (Kalyaanamoorthy et al. 2017). The final tree was visualized in FigTree v.1.4.4 (http://tree.bio.ed.ac.uk/software/figtree/). The same data set excluding the outgroup was also used to construct a NeighborNet phylogenetic network.

In addition, we estimated the species tree using the Bayesian species-tree inference approach SNAPP v1.6.1 (Bryant et al. 2012) implemented in BEAST v.2.7.4 (Bouckaert et al. 2019) based on a data set including only a single SNP for each RAD locus present in at least 80% of individuals. The data set was reduced to four representative individuals of each diploid ingroup species and two of *L. spicata* as outgroup to reduce computation time. The input XML file for SNAPP was prepared in BEAUti v.2.7.4 (Bouckaert et al. 2019) with the default option for a pure birth (Yule) model hyperparameter λ equal to the birth rate of the species tree, which was allowed to vary and was sampled during the MCMC, as recommended by Bryant et al. (2012) and Drummond and Bouckaert (2015). The SNAPP analysis was performed for four independent runs with 3,000,000 generations each and saving a tree every 1000th generation. The coalescent rate and forward (u) and backward (v) mutation rates were sampled from within the MCMC. Mixing of MCMC chains and convergence of the SNAPP analyses were assessed in Tracer v.1.7.2. The four runs were combined in LogCombiner v.2.7.7, discarding 10% of trees as burn-in, and the frequency of alternative topologies was computed in TreeSetAnalyser. Posterior probabilities (PPs) for each clade were computed in TreeAnnotator v.2.7.6, and SNAPP trees were visualized as a cloudogram in DensiTree v.3.0.2 (Bouckaert 2010).

### Analyses of Genetic Structure and Gene Flow among Diploids

We assessed potential admixture among diploid species using Bayesian clustering in STRUCTURE v.2.3.4 (Pritchard et al. 2000). SNPs present in at least 70% of individuals and with maxH_o_ = 0.65 and MAC ≥ 3 were exported from the STACKS catalogue in STRUCTURE format using *populations* with the *–write-random-snp* flag to reduce linkage by including only one random SNP per locus, resulting in 2948 SNPs. STRUCTURE was run with the admixture model for 1,000,000 MCMC generations with 100,000 generations as burn-in for *K* (number of groups) ranging from 1 to 12 with 10 replicates each. We aggregated STRUCTURE results across replicates using CLUMPAK (Kopelman et al. 2015) and inferred the optimal *K* following Evanno et al. (2005) in the R package *pophelper* (Francis 2017), while also considering other biologically meaningful values of *K* (Meirmans 2015). To test for introgression while accounting for the effects of incomplete lineage sorting (ILS), we computed the *D-*statistic (Green et al. 2010) and *f*-branch statistic (Malinsky et al. 2018), constrained with the species tree topology obtained from SNAPP, between all species pairs (max. 50% missing data, minDP = 8) using Dsuite v.0.5 (Malinsky et al. 2021).

### Mixed-Ploidy Variant Calling and Exploratory Analyses of SNP Data

We used GATK v.4.2.5.0 (McKenna et al. 2010) for variant calling of mixed ploidy data sets. Briefly, *HaplotypeCaller* was run in gVCF mode to call haplotypes for each sample individually, with the correct ploidy specified as inferred from RGS estimation. Then, the resulting g.vcf files were combined with *GenomicsDBImport* before joint genotyping for all samples was performed using *GenotypeGVCF*. We used *SelectVariants* to retain only biallelic SNPs and performed quality filtering with *VariantFiltration* following the Broad Institute’s hard filtering recommendations (https://gatk.broadinstitute.org/hc/en-us/articles/360035890471-Hard-filtering-germline-short-variants;  Supplementary Table S4). To allow for accurate genotyping of polyploids, which require higher read depths to accurately infer dosage information (Uitdewilligen et al. 2013), only SNPs with a minimum genotype read depth of 8 for diploids, 30 for tetraploids, and 40 for hexaploids were retained. In addition, genotypes with a coverage higher than 200 were removed to exclude potential paralogues. As for the diploid data set, a baseline VCF was produced, containing high-quality SNPs present in at least 50% of individuals, which was used to construct a NeighborNet network in SplitsTree.

### Inference of Inheritance Modes and Taxonomic Assignment of Tetraploids

We followed the approach of Grünig et al. (2024) to infer the inheritance mode, and thus the type of polyploidy, of tetraploids. This method allows the distinction between di- and tetrasomic inheritance from population-wide genotype frequencies. In short, VCF files with a maximum of 30% missing data were produced for each tetraploid population, and allele frequency tables were retrieved using GATK *VariantsToTable*. Then, we used a custom R script (Grünig et al. 2024) to plot the distribution of genotype frequencies against allele frequencies. Because only two individuals per population were sampled for *L. divulgata*, we merged geographically close populations to increase the number of samples per population, a simplification we believe reasonable because *Luzula* is wind-pollinated and populations can likely interbreed across large distances, as reflected by the low d_XY_ values observed between populations (d_XY_ < 0.06). As a complementary approach for inferring inheritance modes, we used nQuire (Weiß et al. 2018), which does not rely on called genotypes but instead estimates allele depth distributions from raw read depth. BAM files for all tetraploid individuals were denoised using *nQuire denoise*, and smoothed distributions of allelic depth retrieved from *nQuire histo* were plotted in R.

To specifically address genetic structure within the morphologically indistinguishable *L. alpina* and tetraploid *L. multiflora* (alpine tetraploids), we performed principal component analysis (PCA) in *adegenet* and STRUCTURE analysis for this data set. The PCA was based on SNPs with a maximum of 50% missing data and MAC ≥ 3. STRUCTURE was run for *K* ranging from 1 to 10 using 2084 SNPs with MAC ≥ 3 and present in at least 70% of individuals that were linkage-pruned using BCFtools (*r*^2^ < 0.2 in a window of 1000 bp). The assignment of alpine tetraploids to genetic clusters at *K* = 2, together with the placement of samples in monophyletic clades in the ML tree obtained from IQ-TREE 2 (see next paragraph) and chromosome counts, was then used to assign all alpine tetraploid individuals to one of the two species for further analyses.

### Analyses of Monophyly, Genetic Structure, and Gene Flow among Diploids and Polyploids

To investigate whether polyploids formed monophyletic clades, an ML tree of the full data set (SNPs present in at least 50% of samples, MAC ≥ 3) was constructed in IQ-TREE 2 using the same settings as for diploids. However, as a bifurcating tree is not an appropriate representation of the evolutionary history of allopolyploids, we refrained from inferring species relationships from it. To account for the often-reticulate relationships in polyploid complexes, a NeighborNet network based on Nei’s distances and a PCA of genomic variation were produced for the same data set excluding the outgroup. Admixture among species and populations was assessed with STRUCTURE based on 3644 unlinked SNPs with MAC ≥ 3 present in at least 70% of individuals that were linkage-pruned in BCFtools (*r*^2^ < 0.2 in a window of 1000 bp). The resultant VCF file was converted to STRUCTURE format using a custom Python script from J. Gerchen (https://github.com/jgerchen/polyintro/blob/main/workflow/scripts/structure/vcf_to_structure.py) adapted for hexaploids. STRUCTURE was run with the same settings as for diploids but specifying a maximum ploidy of six. Although STRUCTURE has been shown to be robust regarding ploidy (Stift et al. 2019), it can be biased by sample size (Lawson et al. 2018). We, therefore, repeated the same analysis for a balanced subset of 25 individuals per species.

We inferred signatures of gene flow among the species using Dsuite and TreeMix. Notably, introgression can be difficult to distinguish from the genomic signatures of allopolyploidization events (Leal et al. 2024). However, we refer to the inferred signals as “introgression” for better readability and elaborate on this distinction in the Discussion. *D*-statistics and *f*-branch statistics for all possible species trios were computed in Dsuite based on a data set with SNPs present in at least 50% of individuals, constrained with the topology of the ML tree. Genome-wide allele frequency data derived from 8899 SNPs present in at least 80% of individuals were used to test for the presence of gene flow among lineages using TreeMix v.1.12 (Pickrell and Pritchard 2012). TreeMix input files were produced with a Python script from M. Bohutínská (https://github.com/mbohutinska/TreeMix_input/blob/master/conversionTreemixMajda.py) and the analysis was run using a pipeline written by C. Dahms (https://github.com/carolindahms/TreeMix/tree/main). We tested variable numbers of migration edges *m* from 0 to 10, performing 500 bootstrap replicates by resampling blocks of *k* = 100 SNPs to build a consensus tree. The R package *OptM* (Fitak 2021) was then used to infer the best number of migration edges (*m*) based on the change in log likelihood, and 30 independent TreeMix runs were performed for this *m* to compute mean migration weights and *P* values.

### Inference of the Putative Parental Species of Polyploids

The most likely parental species of polyploids were inferred using the method-of-moment estimator of relatedness coefficients implemented in Polyrelatedness (Huang et al. 2014). Based on a data set including SNPs present in at least 50% of individuals, we computed pairwise relatedness coefficients based on allele frequencies among all individuals and plotted their distribution for each polyploid taxon and putative progenitor species. Differences in mean relatedness were assessed with Wilcoxon rank sum tests.

We further explored the origin of tetraploids by applying genomic polarization (Leal et al. 2023). This approach utilizes multiple-sequence alignments (MSAs) to polarize the sequence of a polyploid using another species present in the MSA (i.e., the reference sequence). It effectively masks variants that are identical between polyploid and reference sequence and thus retains only the fraction of the polyploid genome deviating from the reference. If the reference sequence was one of the parental species of an allopolyploid, the remaining polarized sequence is expected to have high similarity to the second parental species. Conducting phylogenetic inference on gene trees derived from these polarized sequences will result in the polyploid pairing with the most similar species after polarization. Thus, the parental species of allopolyploids can be inferred iteratively by selecting different reference sequences until convergence. We followed the pipeline of Leal et al. (2023) but adapted the generation of MSAs for use with RADseq data. In short, genotypes were called for each sample individually, including invariant sites, and quality filtering was performed following GATK hard filtering recommendations, keeping only sites with a minimum read depth of five (Supplementary Table S5). We then used *BCFtools consensus* to produce FASTA files for each sample with heterozygous sites encoded as IUPAC ambiguity codes. As RADseq data are not suited for the computation of gene trees, we instead split each chromosome of the reference sequence into 20 regions with equal numbers of SNPs, resulting in a total of 120 regions, for each of which we produced an MSA from the individual FASTA files. After polarization of the polyploid, IQ-TREE 2 was used to infer an ML tree for each of the MSAs (hereafter referred to as “locus tree” for simplicity). Single-locus phylogenies were summarized to estimate the species tree using the super-tree inference method ASTRAL-III v.5.7.8 (Zhang et al. 2018), which is consistent under the multispecies coalescent and thus able to handle ILS. Finally, the frequency of pairings between the polyploid and all other species/clades included in the phylogeny was computed across individual locus trees. The species sister to the polarized polyploid in the species tree was chosen as reference for the next iteration until the analysis converged. If the polyploid ended up being sister to an entire clade instead of a single species, we used the species within this clade, to which the most frequent pairing occurred, as a reference. The analysis was performed separately for each of the three tetraploid species included in this study, including one representative individual of the polyploid and of all potential parental taxa as well as *L. nivea* as outgroup. In all cases, *L. campestris* was used as the first reference sequence. Because *L. sudetica* has a higher chromosome number (48CL) than any other species, and none of the previous analyses gave any indication of it being involved in the origin of polyploids (see the “Results” section), we excluded it from this analysis.

### Phylogenetic and Phylogeographic Analyses of Plastid Sequences

Bayesian phylogenetic analyses of plastid data were performed using MrBayes v.3.2.7 (Ronquist et al. 2012) applying the F81 (V1) and JC69 (V5) substitution models proposed by the corrected Akaike information criterion (AICc) in MrAIC.pl v.1.4 (Nylander 2004). The alignment was partitioned into nucleotide and indel sets, and indels were treated as morphological data (Lewis 2001). The Metropolis-coupled Markov chain Monte Carlo process included four runs with four chains each (three heated using the default heating scheme), which were run simultaneously for 10,000,000 generations each. Trees were sampled every 1000th generation using default priors. PPs of the consensus tree were determined from all trees after discarding the first 1001 trees of each run as burn-in. Convergence of chains was assessed in TRACER v.1.7.2 (Rambaut et al. 2018). In addition, an ML tree of the same data was inferred in IQ-TREE 2, using 1000 ultrafast bootstrap replicates and the same substitution models as above, with the JC2 model for binary indel data. To assess phylogeographic patterns, we inferred a statistical parsimony network based on the plastid alignment in TCS (Clement et al. 2000), implemented in POPART (Leigh and Bryant 2015), with a connection limit of 95%.

## Results

### Relative GS and Chromosome Number Estimation

Relative GS estimation confirmed three ploidy levels, with RGS ranging from 0.21 to 0.29 for diploids, 0.47 to 0.60 for tetraploids, and 0.77 to 0.84 for hexaploids (Supplementary Table S1). The ploidy level of two samples (Lalp_7_07 and Ltau_210_2) could not be estimated. We produced new chromosome counts for 52 alpine tetraploid individuals, which, combined with previously published counts for six individuals (Pungaršek et al. 2023) also included in this study, yielded 42 counts corresponding to the 12AL + 24BL karyotype of *L. alpina* and 16 to the 24AL karyotype of *L. multiflora* (Supplementary Fig. S2). In addition, we retrieved from literature 13 counts for *L. alpina* and seven for *L. multiflora*.

### Phylogenetic Relationships among Diploids

**Figure 2.**
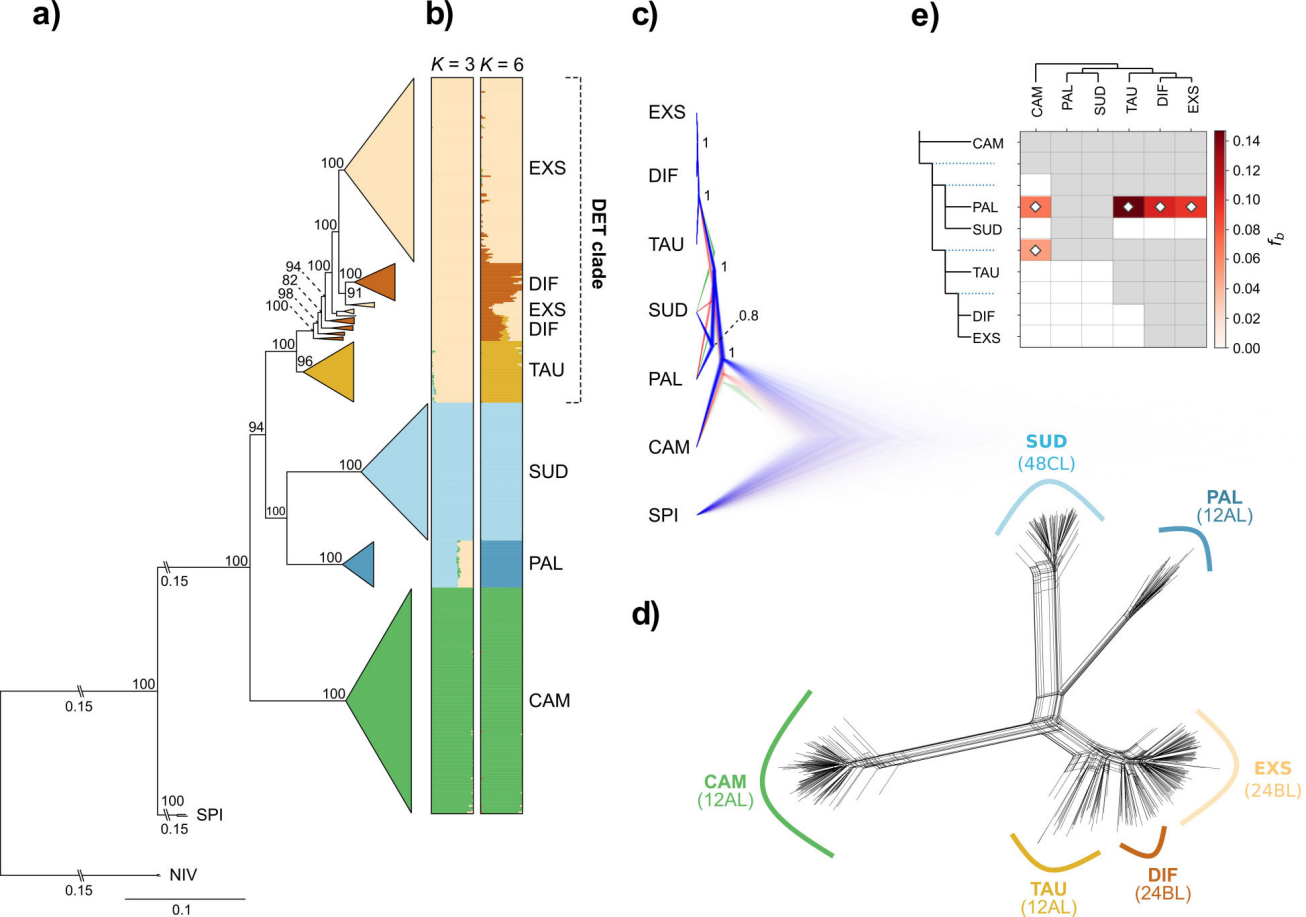
Phylogenetic relationships and gene flow patterns among European diploid species of *Luzula* sect. *Luzula*. a) Best-scoring maximum likelihood tree inferred in IQ-TREE 2 based on 27,664 SNPs derived from RADseq, including *L. nivea* (NIV) and *L. spicata* (SPI) as outgroups. Terminal nodes were collapsed and colored according to species. The DET clade containing *L. divulgatiformis, L. exspectata*, and *L. taurica* is indicated. Numbers above major branches indicate bootstrap support, and numbers (0.15) next to double dashes show branch lengths that were shortened for readability. The full tree is provided in Supplementary Fig. S3. b) Allocation of individuals to genetic clusters inferred by STRUCTURE for *K* = 3 and *K* = 6. c) Species tree estimated by SNAPP with alternative topologies visualized in red and green. Numbers are posterior probabilities. d) NeighborNet network based on Nei’s distance calculated from 25,011 SNPs with species and their karyotypes indicated. e) Introgression inferred by *f*-branch statistics estimated in Dsuite. Gray cells indicate inadmissible comparisons due to topological constraints of the underlying SNAPP tree topology, and dotted lines represent ancestral lineages. White diamonds indicate significant *f*-branch statistics (*P <* 0.05).

After demultiplexing and quality trimming, an average number of 13.15 million (SD = 8.31) reads per sample were mapped against the reference genome of *L. sylvatica*. Variant calling of diploid individuals with STACKS produced 567,744 SNPs. Following initial filtering for missing data, we excluded 12 samples with >50% missing data and two samples, which were identified as putative hybrids or contaminations based on the NeighborNet, retaining a total of 457 samples and 87,921 SNPs for further analyses. The ML phylogenetic tree inferred in IQ-TREE 2 based on the best substitution model (TVM + F + ASC + R6) identified by ModelFinder recovered most diploid species as monophyletic clades with high bootstrap support (Fig. 2a, Supplementary Fig. S3). *Luzula campestris* was sister to all other diploid taxa (BS 100%), which were split into two sister clades. One included *L. pallescens* and *L. sudetica* (BS 100%), and the second clade contained *L. divulgatiformis, L. exspectata*, and *L. taurica* (BS 100%; DET clade hereafter). Within this group, *L. taurica* was retrieved as a monophyletic (BS 96%) sister to a clade with paraphyletic *L. divulgatiformis* forming a grade of diverging lineages, in which three lineages of polyphyletic *L. exspectata* were nested. The species tree estimated by SNAPP showed the same overall topology as the ML tree (Fig. 2c). Most nodes were highly supported (PP = 1), with only the clade comprising *L. pallescens* and *L. sudetica* having lower support (PP = 0.80). Accordingly, the 95% highest posterior densities contained two out of three recovered topologies, with the main topology being supported by 79.6% of trees. In the alternative topology, which was supported by 16.5% of trees, *L. sudetica* was sister to a clade containing *L. pallescens* and the DET clade. The main clades of both phylogenetic trees were also recovered as distinct groups in the NeighborNet phylogenetic network, with *L. divulgatiformis, L. exspectata*, and *L. taurica* showing little differentiation (Fig. 2d).

### Genetic Structure and Gene Flow among Diploids

The optimal number of clusters in the STRUCTURE analysis according to Δ*K* was 3 (Supplementary Fig. S4), corresponding to the major clades of the ML tree (Fig. 2b, Supplementary Fig. S5). *Luzula pallescens* showed considerable admixture with the DET clade. Considering *K* = 6 as a biologically meaningful grouping (equal to the number of species and also represented by a second peak in Δ*K*; Supplementary Fig. S4), strong admixture was revealed within the DET clade, particularly between *L. divulgatiformis* and *L. exspectata* and, to a lesser degree, between *L. divulgatiformis* and *L. taurica* (Fig. 2b). When testing for potential introgression among all diploid species pairs with Dsuite, ten trios had significant (*P <* 0.05) *D*-statistics ranging from 0.04 to 0.23 (Supplementary Table S6) and *f4*-ratios between 0.04 and 0.15. The *f*-branch statistic (*f_b_*) revealed significant excess allele sharing indicative of introgression between all members of the DET clade and *L. pallescens*, with a particularly strong signal (*f_b_* = 0.15) for *L. taurica* (Fig. 2e). Weaker signals of introgression were also found between *L. campestris* and *L. pallescens*, as well as between *L. campestris* and the ancestral branch leading to the DET clade.

### Inheritance Mode and Taxonomic Assignment of Tetraploids

**Figure 3.**
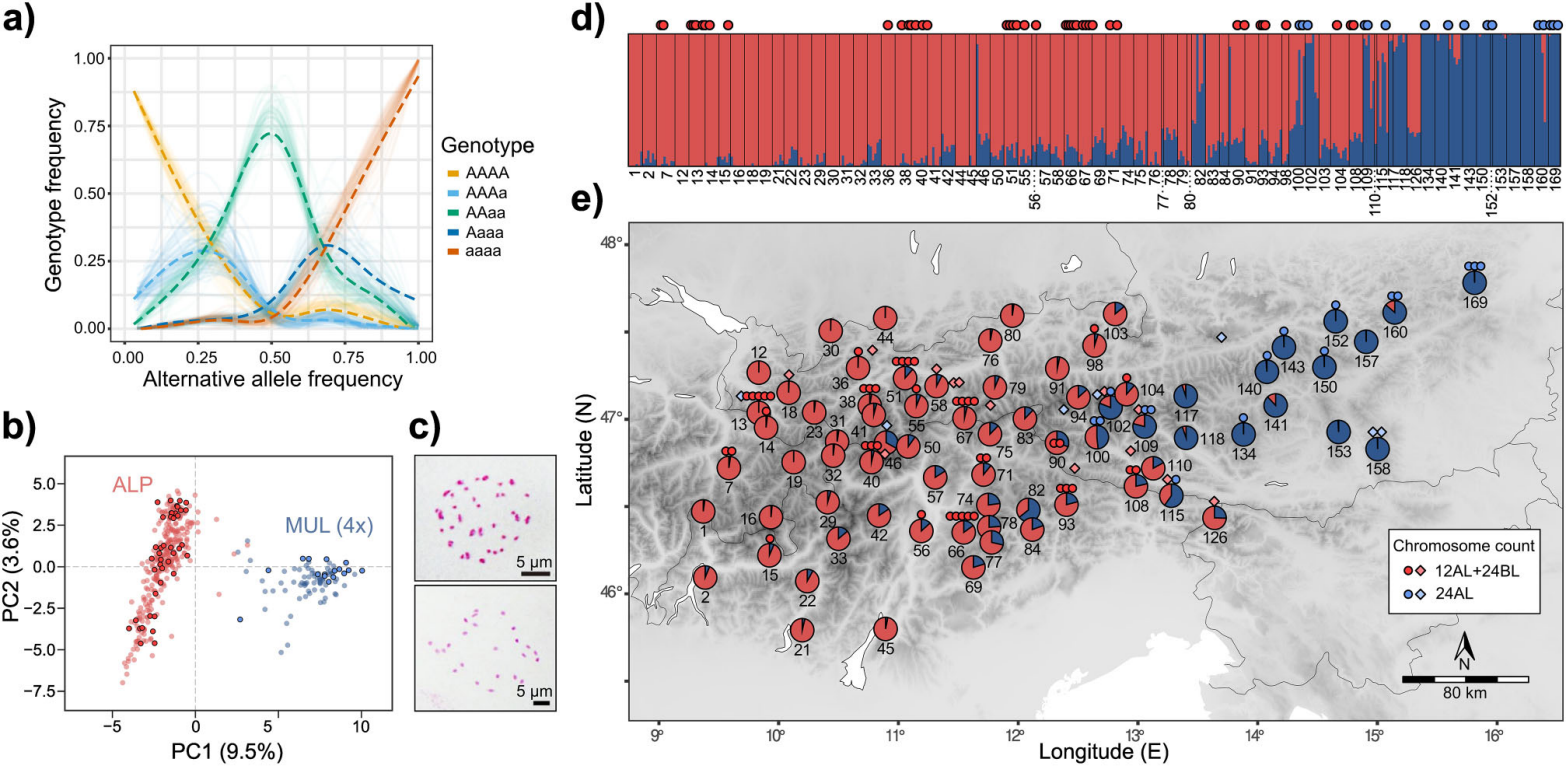
Inheritance mode, chromosome counts, and genetic structure of alpine tetraploid species of *Luzula* sect. *Luzula*. a) Genotype frequencies (*y*-axis) relative to alternative allele frequency (*x*-axis) for the alpine tetraploids *L. alpina* and *L. multiflora*. Thin lines are per-population frequencies, and thick broken lines represent smoothed averages across all populations. Genotype frequencies for individual populations are given in Supplementary Fig. S6a. b) PCA of genetic variation among alpine tetraploids along PC1 and PC2 with colors corresponding to *L. alpina* (red) and *L. multiflora* (blue). c) Metaphase chromosomes for *L. alpina* (2*n* = 4*x* = 12AL + 24BL, population 38, top) and *L. multiflora* (2*n* = 4*x* = 24AL, population 169, bottom). d) STRUCTURE results at *K* = 2 for each individual grouped by population as indicated by population identifiers below the bar charts. e) Pie charts showing population-averaged assignment of alpine tetraploid *L. alpina* and *L. multiflora* to genetic clusters inferred by STRUCTURE for *K* = 2. Small dots in (b), (d), and (e) represent chromosome counts for individuals included in this study, either newly produced or from Pungaršek et al. (2023), corresponding to the 12AL + 24BL cytotype of *L. alpina* (red) and the 24AL cytotype of *L. multiflora* (blue). Additional chromosome counts retrieved from literature are shown as diamonds of lighter colors in (e).

**Figure 4.**
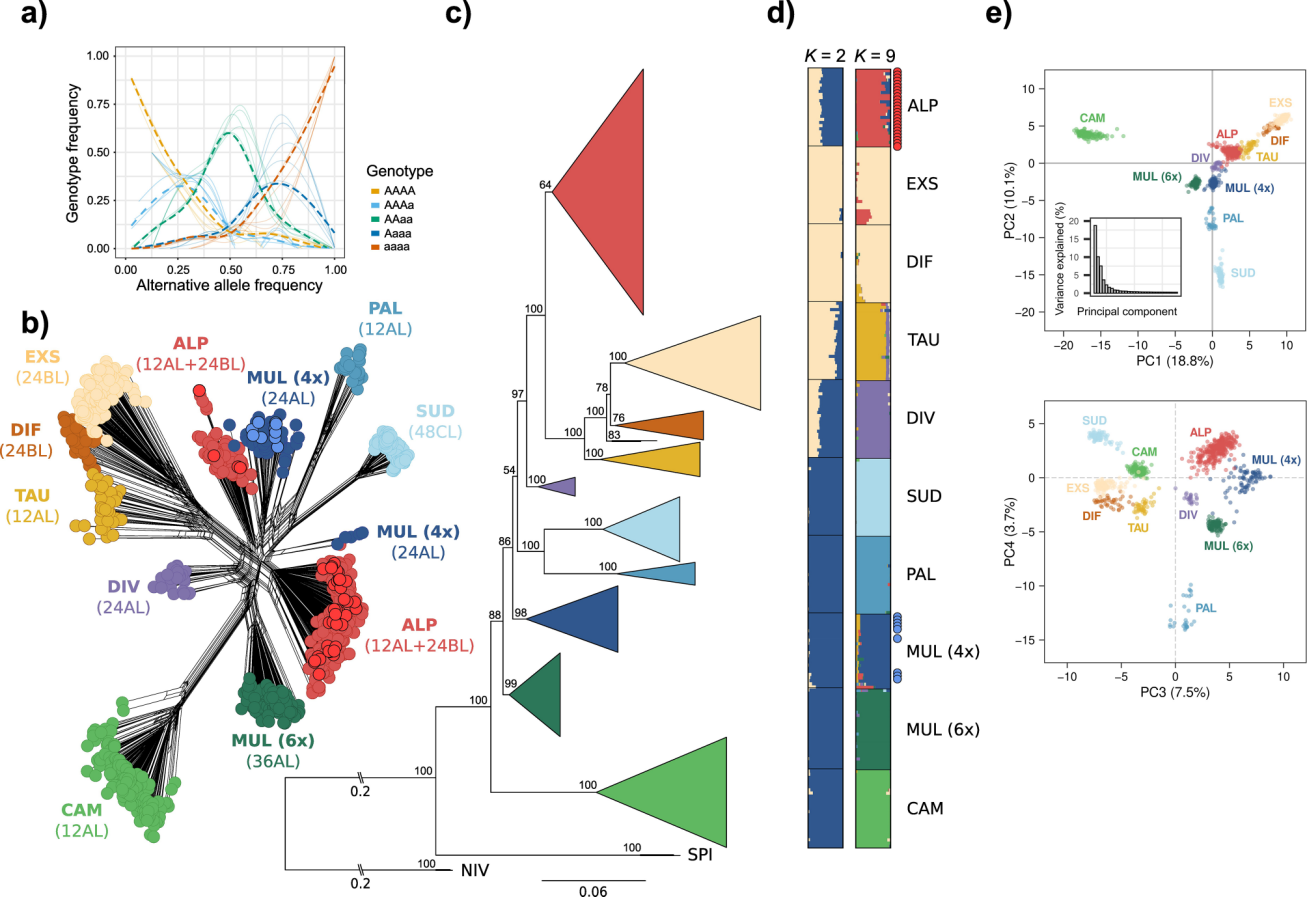
Genetic structure among European diploid and polyploid species of *Luzula* sect. *Luzula*. a) Genotype frequencies (*y*-axis) relative to alternative allele frequency (*x*-axis) for tetraploid *L. divulgata*. Thin lines are per-population frequencies, and thick broken lines represent smoothed averages across all populations. Genotype frequencies for individual populations are given in Supplementary Fig. S6b. b) NeighborNet phylogenetic network based on Nei’s distance calculated from 13,954 SNPs. Individuals are represented by dots colored according to species; individuals with chromosome counts corresponding to the 12AL + 24BL cytotype of *L. alpina* and the 24AL cytotype of *L. multiflora* (4*x*) are shown as red and blue circles with black outlines, respectively. Species and their karyotypes are indicated next to major clusters. c) Best-scoring maximum likelihood tree of diploid and polyploid members of *Luzula* sect. *Luzula* and the outgroup (NIV, *L. nivea*; SPI, *L. spicata*) inferred in IQ-TREE 2 based on 9112 SNPs derived from RADseq. Terminal nodes were collapsed for better legibility and colored according to species. Numbers above major branches indicate bootstrap support, and numbers (0.2) next to double dashes show branch lengths that were shortened for readability. The full tree is provided in Supplementary Fig. S12. d) Assignment of *Luzula* sect. *Luzula* individuals from a subsampled data set with equal group sizes to genetic clusters inferred by STRUCTURE for *K* = 2 and *K* = 9. Red and blue dots represent individuals with available chromosome counts as in (b). e) PCA of genetic variation along PC1 and PC2 (upper panel) and PC3 and PC4 (lower panel). The proportion of variance explained by each principal component is indicated in the axis labels, and the eigenvalues of the first 30 principal components are shown as bar plot.

A total of 869,020 biallelic SNPs were called using GATK for the data set including diploid and polyploid species. After initial quality filtering for read depth and missing data, 29,623 SNPs were retained. We removed six samples with >50% missing data and three putative hybrids or contaminated samples, resulting in a final data set of 1002 samples for subsequent analyses. All alpine tetraploid populations belonging to *L. alpina/L. multiflora* exhibited an excess of genotypes with intermediate allelic depth, consistent with expectations of Hardy–Weinberg equilibrium under disomic inheritance as found in allopolyploids (Fig. 3a, Supplementary Fig. S6a). This was confirmed at the level of individuals by nQuire, revealing an excess of intermediate allelic depths for all but six individuals, which showed a pattern more in line with tetrasomic inheritance, typical for autopolyploids (Supplementary Fig. S7a,b). In the case of *L. divulgata*, genotype frequencies were less conclusive and did not fully correspond to expectations under disomic, nor under tetrasomic inheritance (Fig. 4a, Supplementary Fig. S6b). Along the same line, some individuals of this species had an excess of intermediate allelic depths, whereas others showed a pattern more indicative of autopolyploidy (Supplementary Fig. S7c). However, because the six putatively autopolyploid alpine tetraploids were nested within the remaining alpine tetraploids across all analyses, and *L. divulgata* formed a single well-supported clade, we did not exclude samples with aberrant genotype frequencies from further analyses.

Alpine tetraploids segregated into two main groups in the PCA, corresponding to chromosome counts pertaining to either *L. alpina* (12AL + 24BL) or *L. multiflora* (24AL, Fig. 3b,c). STRUCTURE analysis of alpine tetraploids at *K* = 2, which was proposed by Δ*K* (Supplementary Fig. S8), revealed a west-to-east geographical pattern that again largely corresponded to the two karyotypes found through chromosome counts (Fig. 3d,e, Supplementary Figs. S2 and S9). The western cluster contained only counts corresponding to *L. alpina* (12AL + 24BL), whereas the eastern cluster contained exclusively the 24AL karyotype pertaining to *L. multiflora*. Individuals from several populations in the longitudinally central part of the Eastern Alps were assigned to either of the two genetic groups or showed signs of admixture, resulting in several mixed populations with co-occurring karyotypes in some cases (Fig. 3d,e). Following the segregation of alpine tetraploids into two groups in the PCA (Fig. 3b), STRUCTURE analyses (Figs. 3d,e and 4d, Supplementary Figs. S9–S11) and in the ML phylogenetic tree (Fig. 4c, Supplementary Fig. S12), which corresponded to available chromosome counts, we assigned alpine tetraploids to either *L. alpina* (316 individuals) or *L. multiflora* (86 individuals) for subsequent analyses.

### Genetic Structure within Diploids and Polyploids

Most species formed well-defined groups in the nearly star-like NeighborNet phylogenetic network, except for the poorly differentiated DET clade (Fig. 4b). Tetraploid *L. divulgata* was positioned near the center of the network, whereas hexaploid *L. multiflora* and *L. campestris* and *L. pallescens* and *L. sudetica* shared some common splits, respectively, and were positioned on opposite sides of the network. All other clusters had intermediate positions between them. The alpine tetraploids *L. alpina* and *L. multiflora* were split into four more or less coherent clusters. The two largest tetraploid clusters corresponded to *L. alpina*, which was closer to the DET cluster, whereas the two smaller clusters were closer to *L. pallescens* and *L. sudetica*, and pertained to tetraploid *L. multiflora*.

The major genetic groups present in the phylogenetic network were also recovered in the ML tree based on the SYM + ASC + R8 substitution model (Fig. 4c, Supplementary Fig. S12). Most species formed highly supported clades, except *L. alpina*, for which the support was notably lower (BS 64%). Relationships within the DET clade remained complex, as in the analysis of diploids, and *L. divulgatiformis* was paraphyletic and formed two subsequently divergent lineages with monophyletic *L. exspectata* (BS 100%) nested within. The tree topology was reflected in the PCA, in which *L. campestris* was clearly most divergent along PC1, and *L. sudetica* and *L. pallescens* were separated from the remaining species along PC2 (Fig. 4e). Mirroring the blurred species boundaries inferred from phylogenetic trees and NeighborNet analyses, *L. divulgatiformis* and *L. exspectata* were mostly overlapping along PC1 and PC2; however, some separation between the two species was visible along PC4. All polyploid taxa were placed near the center of the ordination when considering the first two principal components; *L. alpina* was shifted toward the DET clade, whereas *L. divulgata* and tetraploid *L. multiflora* were particularly close to each other and only slightly separated along PC2. Tetraploid *L. multiflora* was shifted toward *L. pallescens*, and *L. divulgata* was placed between hexaploid *L. multiflora* and *L. taurica*.

The optimal number of clusters in the STRUCTURE analysis of the data set subsampled to even group sizes according to Δ*K* was 2 (Supplementary Fig. S13), corresponding to the DET clade on one hand, and most of the remaining lineages on the other hand (Fig. 4d, Supplementary Fig. S10). Notably, the tetraploids *L. alpina* and *L. divulgata* were admixed between both clusters, and weak signals of admixture were also present in *L. taurica*. An additional smaller peak in Δ*K* was found for *K* = 9, for which all species but *L. exspectata* and *L. divulgatiformis* formed their own cluster. The latter showed some admixture with *L. taurica*, whereas *L. exspectata* had signals of admixture with *L. alpina*, which showed weak admixture with *L. exspectata* and tetraploid *L. multiflora*. A small proportion of genetic admixture with *L. divulgata* was present in *L. taurica*, and weak admixture with *L. alpina* and *L. taurica* was inferred for tetraploid *L. multiflora*. STRUCTURE analysis conducted on the full data set with uneven group sizes yielded different results, with the best *K* being 4, followed by 7 and 9 (Supplementary Fig. S14), corresponding to some of the major lineages but recovering *L. divulgata* and *L. pallescens* as admixed and revealing considerable substructure within *L. alpina* (Supplementary Fig. S11).

### Signatures of Introgression among Diploids and Polyploids

**Figure 5.**
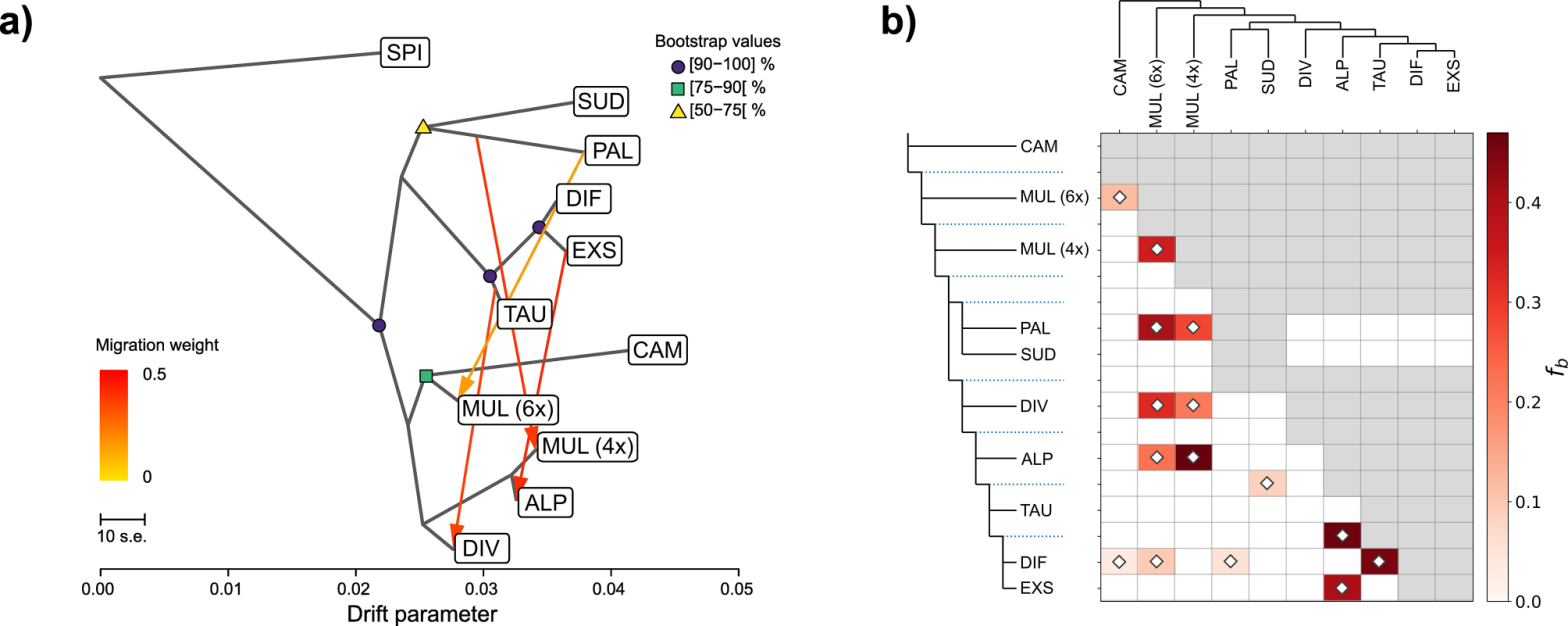
Signatures of introgression among diploid and polyploid species of *Luzula* sect. *Luzula*. a) Consensus TreeMix tree for *m* = 4 migration edges. Migration events correspond to the events with the highest likelihood among the 500 replicates. Bootstrap percentages are represented by colored symbols and migration weights are indicated by the color scale. b) Introgression inferred by the *f*-branch statistic estimated in Dsuite. Gray cells indicate inadmissible comparisons due to topological constraints of the underlying tree topology and dotted lines represent ancestral lineages. White diamonds indicate significant *f*-branch statistics (*P <* 0.05).

The optimal number of migration edges in the TreeMix analysis was four (Supplementary Fig. S15). Albeit the diploid clades were also recovered in the TreeMix tree, the positioning of polyploids differed from that of the ML tree, with hexaploid *L. multiflora* being a sister to *L. campestris* and all tetraploids forming a single clade (Fig. 5a). Most relationships, however, received low bootstrap support. Significant (*P <* 0.001) ancient gene flow was inferred from *L. taurica* to *L. divulgata* (migration weight, MW = 0.36) and from *L. pallescens* to tetraploid *L. multiflora* (MW = 0.38). More recent gene flow occurred from *L. exspectata* to *L. alpina* (MW = 0.41) and, albeit weaker, from *L. pallescens* to hexaploid *L. multiflora* (MW = 0.16).

*D*-statistics were significant for 63 species trios, ranging from 0.05 to 0.34; *f4*-ratios were between 0.03 and 0.68 (Supplementary Table S7). The strongest signals of introgression were observed between *L. pallescens* and hexaploid *L. multiflora* (species trio: EXS-PAL-MUL(6*×*); *D*-statistic = 0.34), *L. alpina* and tetraploid *L. multiflora* (EXS-ALP-MUL(4*×*); *D*-statistic = 0.30), as well as between tetra- and hexaploid *L. multiflora* (EXS-MUL(4*×*)-MUL(6*×*); *D*-statistic = 0.30). Largely congruent with the TreeMix results, *f*-branch statistics computed constrained with the ML tree topology revealed strong gene flow between tetraploid *L. multiflora* and the three species *L. alpina* (*f_b_* = 0.47), *L. pallescens* (*f_b_* = 0.28), and *L. divulgata* (*f_b_* = 0.22) (Fig. 5b). Strong signatures of introgression were found between *L. alpina* and *L. exspectata* (*f_b_* = 0.40), as well as between *L. alpina* and the common ancestor of *L. exspectata* and *L. divulgatiformis* (*f_b_* = 0.46). Unlike in the TreeMix analysis, no signal of introgression between *L. divulgata* and *L. taurica* could be detected. The latter, however, exhibited excess allele sharing with *L. divulgatiformis* (*f_b_* = 0.45). Hexaploid *L. multiflora* showed signals of introgression with several species, namely, tetraploid *L. multiflora* (*f_b_* = 0.35), *L. pallescens* (*f_b_* = 0.40), and *L. divulgata* (*f_b_* = 0.32), as well as weaker signals with *L. alpina* (*f_b_* = 0.23), *L. campestris* (*f_b_* = 0.12), and *L. divulgatiformis* (*f_b_* = 0.09).

### Inference of the Putative Parental Species of Polyploids

**Figure 6.**
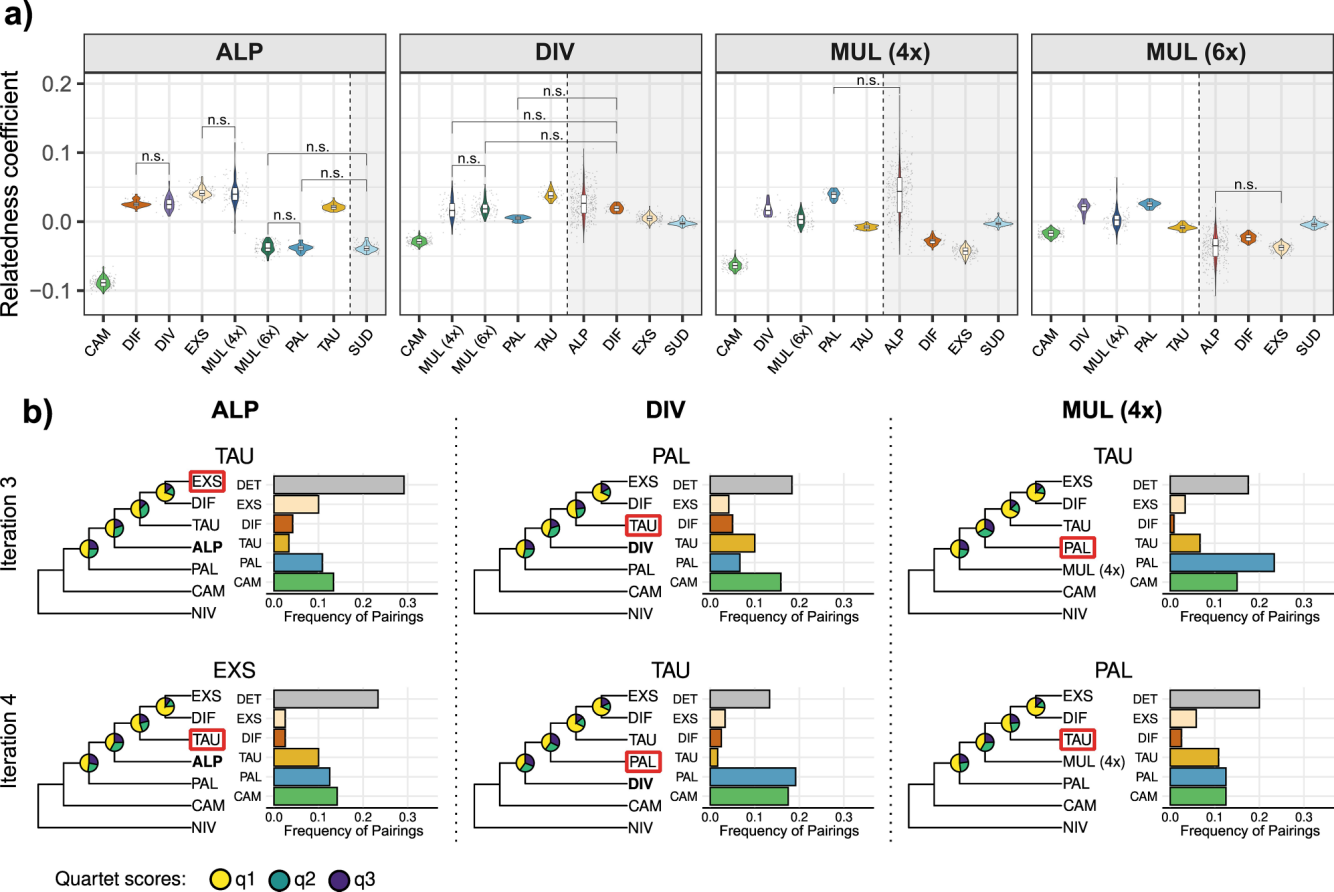
Inference of the most likely progenitors of polyploid species of *Luzula* sect. *Luzula*. a) Violin plots of relatedness coefficients of tetra- and hexaploid *Luzula* species to potential ancestors (left of the dashed line) and species that can be excluded as ancestral taxa based on karyology (right of the dashed line and shaded gray). All pairwise comparisons were significant (*P* < 0.05) according to Wilcoxon rank sum tests except those indicated (n.s.). b) Results of iterative genomic polarization of tetraploid *L. alpina* (left), *L. divulgata* (middle), and *L. multiflora* (4*x*) (right). Species tree cladograms obtained with ASTRAL based on 120 “locus trees” are presented for the last two iterations that represent convergence of the analysis. The species used as a reference sequence for polarization is indicated at the top, and the tetraploid is highlighted in bold. Pie charts show quartet support for each branch. Bar plots on the right show pairing frequencies of the polarized tetraploid with other species across the 120 “locus trees” inferred in IQ-TREE 2, with colors corresponding to species and gray corresponding to the DET clade. The species with the highest pairing within the sister clade of the tetraploid is highlighted with a red box in the cladograms and was chosen as the reference sequence for the next iteration. The results of all four iterations are shown in Supplementary Fig. S16.

Relatedness coefficients inferred in Polyrelatedness were used to determine the most likely progenitors of polyploids. *Luzula exspectata* and tetraploid *L. multiflora* had the highest relatedness coefficients with respect to *L. alpina* (mean ± SD: 0.04 ± 0.01 and 0.04 ± 0.02, respectively; Fig. 6a), which were both slightly, yet significantly, higher than those of the second-ranked *L. divulgata* and *L. divulgatiformis* (Wilcoxon rank sum test *P <* 0.001). In the case of *L. divulgata*, the highest relatedness was found for *L. taurica* (0.04 ± 0.01), followed (*P <* 0.001) by karyologically divergent *L. alpina* (0.03 ± 0.02), which exhibited exceptionally high variation in relatedness coefficients across individuals, *L. divulgatiformis* (0.02 ± 0.00), and both cytotypes of *L. multiflora* (0.02 ± 0.02 and 0.02 ± 0.01, respectively). *Luzula pallescens* (0.04 ± 0.01) and *L. alpina* (0.04 ± 0.04) had significantly higher relatedness to tetraploid *L. multiflora* than all other species (*P <* 0.001), followed by *L. divulgata* and hexaploid *L. multiflora*. The hexaploid *L. multiflora* had high relatedness to *L. pallescens* (0.03 ± 0.00), *L. divulgata* (0.02 ± 0.01), and tetraploid *L. multiflora* (0.00 ± 0.02). Notably, the hexaploid was the only case in which phylogenetically distant *L. campestris* had a degree of relatedness comparable to that of other species (−0.02 ± 0.01), even surpassing those of *L. divulgatiformis* and *L. exspectata*.

As a complementary approach, the most likely diploid progenitors of tetraploids were inferred using genomic polarization. Upon convergence, polarized *L. alpina* paired with the entire DET clade (Fig. 6b, Supplementary Fig. S16a, Supplementary Results). Within this clade, the individual species for which most pairings occurred were *L. exspectata* and *L. taurica*. In the case of *L. divulgata*, the analysis converged to *L. taurica* and *L. pallescens* as likely parental species (Fig. 6b, Supplementary Fig. S16b, Supplementary Results). However, also here most pairings across locus trees were with the entire DET clade rather than with an individual species when using *L. pallescens* as the reference sequence. A similar result was obtained for tetraploid *L. multiflora*, for which genomic polarization again suggested *L. taurica* and *L. pallescens* as parental species, with a particularly strong signal of the latter (Fig. 6, Supplementary Fig. S16c, Supplementary Results).

### Phylogenetic and Phylogeographic Analyses of Plastid Sequences

Although *Luzula* sect. *Luzula* was recovered as monophyletic in Bayesian and ML phylogenetic analyses of plastid sequences, relationships among species were largely unresolved, and many samples formed a polytomy (Supplementary Fig. S17). The only species that was monophyletic was *L. sudetica*. Another clade with relatively high support (PP > 0.9, BS > 90%) comprised many, but not all, individuals of the four polyploid species. Phylogeographic analyses of plastid sequences revealed 67 distinct haplotypes, which exhibited a structure that was partly taxonomic and partly geographic (Supplementary Fig. S18). *Luzula sudetica* and *L. taurica* were distinct, whereas the other diploid species shared many haplotypes (Supplementary Fig. S19). Alpine tetraploid *L. multiflora* and *L. alpina* mostly shared the same haplotype, although a considerable number of *L. alpina* populations had a common haplotype with *L. exspectata*. Hexaploid *L. multiflora* showed a high diversity of haplotypes, with no apparent pattern, although the predominant haplotype of alpine tetraploids was also present in hexaploid *L. multiflora* and *L. divulgata*.

## Discussion

The crucial role of hybridization in plant evolution has already been recognized by Stebbins (1959) and recent progress in genomics has led to numerous studies providing examples of hybrid speciation in both plants and animals (Soltis and Soltis 2009; Abbott et al. 2013; Svardal et al. 2020; Wu et al. 2022; Rosser et al. 2024). Even though there is limited evidence for homoploid hybridization being directly involved in speciation (Schumer et al. 2014; Yakimowski and Rieseberg 2014; Nieto Feliner et al. 2017), except for the well-studied examples of *Iris* (Arnold 1993) and *Helianthus* (Rieseberg et al. 1995; Owens et al. 2023), hybridization accompanied by genome duplication (i.e., allopolyploidy) is widely acknowledged as an important evolutionary driver (Soltis and Soltis 2009; Brandrud et al. 2020; Debray et al. 2022; Španiel et al. 2023). Our large-scale analysis, integrating genome-wide ddRADseq with plastid markers and chromosome counts, identifies *Luzula* sect. *Luzula* as a plant lineage whose evolutionary history has been shaped by both homoploid hybridization and allopolyploidy, as well as by interploidy gene flow. Although homoploid hybridization in our study group is limited to introgression without the establishment of a stable hybrid lineage, polyploid hybridization resulted in novel allopolyploid species.

### Introgression and Partially Blurred Species Boundaries among Diploids

Most diploid species included in this study were recovered as monophyletic (Fig. 2a), with *L. campestris* constituting the earliest diverging lineage and the remaining species segregating into two main clades: the DET clade comprising *L. divulgatiformis, L. exspectata*, and *L. taurica*, and a second clade containing *L. pallescens* and *L. sudetica*. The genetic divergence of *L. campestris* is consistent with its morphological distinctiveness, as it is the only species of the group that forms long stolons (Bačič et al. 2019). Even though phylogenetic inference from concatenated RAD loci does not account for heterotachy and may be biased due to hybridization and ILS (Degnan 2013; Liu et al. 2015; Som 2015), our phylogenomic results are largely congruent between concatenation (Fig. 2a) and single-locus-based (Fig. 2c) approaches. They are in line with findings of Carrizo García et al. (2025) and offer greatly improved resolution of species relationships within *Luzula* sect. *Luzula* compared with earlier studies (Drábková et al. 2006; Záveská Drábková and Vlček 2010).

Our findings support two putatively independent transitions toward agmatoploidy in the two major diploid clades (Carrizo García et al. 2025), namely in the DET clade giving rise to *L. divulgatiformis* and *L. exspectata*, as well as a second fragmentation event associated with the origin of *L. sudetica*. Our analyses also confirm blurred species boundaries within the DET clade (Carrizo García et al. 2025), particularly between *L. divulgatiformis* and *L. exspectata* (Figs. 2a,b,e and 4d). Both taxa are agmatoploids (24BL), likely derived from *L. taurica* (12AL) via chromosome fission, yet our results cast serious doubts on their recognition as separate species, especially in light of their morphological similarity (Bačič et al. 2007b).

Conflicting topologies in the species tree (Fig. 2c) and admixture between the DET clade and *L. pallescens* (Fig. 2b) correspond with patterns of excess allele sharing revealed by *f*-branch statistics (Fig. 2e), and are, thus, likely signatures of introgression rather than ILS. Specifically, introgression most probably occurred between *L. taurica* and *L. pallescens*, which share the same karyotype (12AL). In contrast, the weaker signatures of gene flow between *L. pallescens* and both *L. divulgatiformis* and *L. exspectata* are probably the result of shared ancestry among lineages of the DET clade (Malinsky et al. 2021). Although rate heterogeneity among lineages may affect the sensitivity and accuracy of *D* statistics and the derived *f*-statistics, resulting in false positives and incorrectly inferred admixture graphs (Frankel and Ané 2023, 2025), we consider our results to be robust, as the major introgression events inferred from these metrics are in line with the results of complementary analyses such as SNAPP and TreeMix. Hybridization among diploid species of *Luzula* sect. *Luzula* was observed in natural populations and confirmed through controlled crossing experiments by Nordenskiöld (1956). Notably, in those experiments, *L. pallescens* was among the species that could be crossed most easily and produced the highest proportion of fertile offspring. Although diploid (12AL) *L. campestris* and *L. pallescens* can also hybridize with agmatoploid *L. sudetica* (48CL), these crosses were sterile (Nordenskiöld 1956). The experimental results of Nordenskiöld were later verified in natural populations (Kirschner 1991), suggesting that *L. sudetica* was likely not involved in the origin of polyploids – a conclusion also supported by our genomic data.

### Evidence for an Allopolyploid Origin of Polyploids

**Figure 7.**
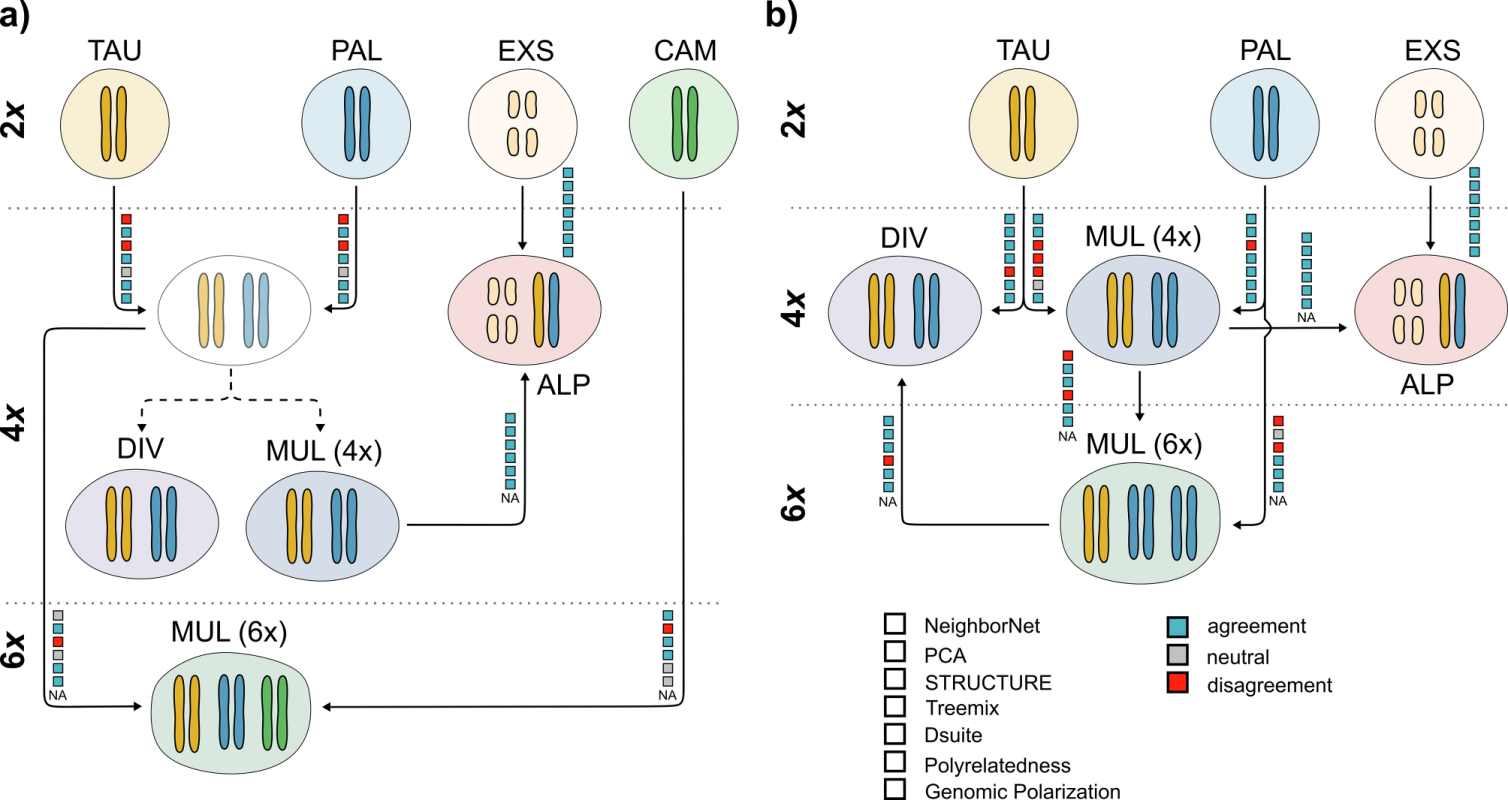
Synthesis across analyses and most likely evolutionary scenarios for the polyploid complex of European *Luzula* sect. *Luzula*. Ploidy levels are indicated on the left and separated by dotted horizontal lines. Karyotypes are depicted schematically with colors representing ancestral diploid subgenomes and large and small chromosome sizes corresponding to AL and BL chromosomes, respectively. Black arrows show proposed hybridization events, and dashed arrows represent evolutionary divergence without hybridization. Analyses supporting or objecting the involvement of each species in a given hybridization event are indicated as colored squares. Genomic polarization is currently not applicable to hexaploids and cannot accommodate allopolyploids as progenitor species and is thus indicated as NA for such cases. a) Hypothesized evolutionary scenario in which the tetraploids *L. divulgata* and *L. multiflora* (4*x*) have a common allopolyploid origin from *L. taurica* and *L. pallescens*. This common ancestor then hybridized with *L. campestris*, giving rise to hexaploid *L. multiflora* (6*x*) in a second allopolyploidization event. After divergence of *L. divulgata* and *L. multiflora* (4*x*), the latter was involved in the origin of *L. alpina* via interploidy hybridization with *L. exspectata*. b) Alternative scenario in which tetraploid *L. multiflora* (4*x*) arose via allopolyploidization from *L. taurica* and *L. pallescens*. It then gave rise to *L. alpina* via interploidy hybridization with diploid *L. exspectata*, and a second allopolyploidization event involving backcrossing with *L. pallescens* resulted in the hexaploid *L. multiflora* (6*x*). The latter then hybridized with *L. taurica*, giving rise to tetraploid *L. divulgata*.

Both tetraploid *L. multiflora* and *L. alpina* exhibited genotype frequency patterns indicative of disomic inheritance typical for allopolyploids (Fig. 3a, Supplementary Figs. S6a and S7a,b; Lloyd and Bomblies 2016; Scott et al. 2023). Although results for *L. divulgata* were less conclusive and did not fully conform to expectations under di- nor polysomic inheritance (Fig. 4a, Supplementary Figs. S6b and S7c), they nonetheless align more closely with an allopolyploid origin. Although disomic inheritance is no definitive proof of allopolyploidy and old autopolyploids are expected to transition toward disomic inheritance over time (Le Comber et al. 2010; Parisod et al. 2010), we consider a hybrid origin of the polyploid *Luzula* species the most plausible scenario, based on multiple lines of supporting evidence discussed in the upcoming paragraphs. Notably, all four polyploid taxa formed distinct groups across phylogenetic analyses (Fig. 4b,c,e), rather than appearing nested within diploid clusters, as would be expected for recent autopolyploids. While our data suggest an allopolyploid origin of all four polyploid species, their progenitors could not be unequivocally identified in each case. The most likely evolutionary scenario on the origin of the polyploids based on our data and a plausible alternative are illustrated in Figure 7.

### A Common Allopolyploid Origin of Tetraploid L. multiflora and L. divulgata

Tetraploid *L. multiflora* consistently exhibited strong genetic affinities with *L. pallescens* across analyses, rendering the latter a likely parental species. This aligns with earlier findings that artificial tetraploids produced from *L. pallescens* closely resemble *L. multiflora* morphologically (Nordenskiöld 1956), and is consistent with the hypothesized close relationship between the two species proposed by Kirschner (1992). However, whereas Kirschner (1992) suggested an autopolyploid origin of tetraploid *L. multiflora* from *L. pallescens –* with the exception of some Irish populations identified as allopolyploid (Jarolímová and Kirschner 1995; Kirschner 1995) – our results instead point to an allopolyploid origin. Specifically, they suggest *L. taurica* as the most likely second parental species, albeit with lower confidence (Fig. 7a). A similar but quantitatively different pattern was found for *L. divulgata*, which shows strong genetic affinity to *L. taurica*, though the identity of its second progenitor remains uncertain. Genomic polarization consistently identified *L. taurica* or the DET clade and *L. pallescens* as the most likely progenitors of both tetraploids, albeit with a clearer signal of *L. pallescens* in the case of *L. multiflora* (Fig. 6b). Ambiguous outcomes of genomic polarization analyses, where all tetraploids were consistently recovered as sisters to the entire DET clade rather than to any individual species, are likely the result of weak genetic differentiation within the DET clade mentioned above. Both *L. divulgata* and tetraploid *L. multiflora* occupied intermediate positions between *L. taurica* and *L. pallescens* in the PCA (Fig. 4e), further supporting a hybrid origin from these two diploids, which is also in agreement with the inferred patterns of introgression (Fig. 2c,e).

Whereas independent allopolyploidization events involving the same diploids cannot be excluded and have been evidenced in other plant genera (Perrie et al. 2010; Guo et al. 2013; Brandrud et al. 2020), we argue that a common origin of *L. divulgata* and tetraploid *L. multiflora* is the most likely scenario, given that the two species exhibit high relatedness coefficients to each other (Fig. 6a). Subsequent divergence of the two taxa may then have been accompanied by differential introgression, from *L. taurica* in the case of *L. divulgata* (Fig. 5a) and from *L. pallescens* in the case of tetraploid *L. multiflora* (Fig. 5a,b). Although it is difficult to disentangle the genomic signatures of allopolyploidization from more recent gene flow (Leal et al. 2024), such post-polyploidization introgression from either of the parental species could explain the apparently closer relationship of *L. divulgata* with *L. taurica* and of *L. multiflora* with *L. pallescens*, as reflected by relatedness coefficients (Fig. 6a). Unidirectional gene flow from diploid progenitors to polyploids has been reported in both auto- (Arnold et al. 2015; Grünig et al. 2024) and allopolyploids (Ma et al. 2010; Nibau et al. 2022). Additionally, introgression from different diploid lineages in different parts of the polyploid’s range may even confer local adaptation (Arnold et al. 2015) and result in different forms of a tetraploid, where each new form resembles more closely the local diploid (Stebbins 1956). The absence of admixture signals between *L. multiflora* and *L. taurica* (Fig. 5a,b) may, therefore, reflect a lack of ongoing or relatively recent gene flow between these species. Instead, more recent admixture with *L. pallescens* (Fig. 5a) and *L. alpina* (Fig. 3d), as previously reported (Nordenskiöld 1956; Kirschner 1991), may have obscured the older genomic signature of the allopolyploidization event.

Even though we consider a common origin of *L. divulgata* and tetraploid *L. multiflora* to be the most parsimonious explanation for the observed genomic patterns, our results are not definitive. Notably, the absence of shared splits between the two species in the NeighborNet phylogenetic network (Fig. 4b) challenges the single-origin hypothesis. Instead, the intermediate position of *L. divulgata* between *L. taurica* and hexaploid *L. multiflora* in the network and PCA (Fig. 4b,e), along with high relatedness to both species (Fig. 6a), raises the alternative possibility of a hybrid origin for *L. divulgata* involving *L. taurica* and hexaploid *L. multiflora* (Fig. 7b). Under this scenario, the apparent genomic affinity of *L. divulgata* to *L. pallescens* may be a spurious signal, possibly arising from hexaploid *L. multiflora* (a likely descendant of *L. pallescens*; see below), acting as a genetic bridge (McDonald et al. 2008; Grant and Grant 2020; Leal et al. 2024). However, hybridization between hexaploids and diploids is rarely found in nature (Dewey 1973; Asay and Dewey 1979; Ingram and Noltie 1995; Hegarty et al. 2012), and even if it occurs, no adult plants have been found (Castro et al. 2011, 2012). Given the general rarity of such hybridization events in nature, we consider a hybrid origin of *L. divulgata* via crosses between *L. taurica* and hexaploid *L. multiflora* less likely than a shared tetraploid ancestor with *L. multiflora*, followed by differential introgression from the parental diploid species.

### Hexaploid L. multiflora Originated from Allopolyploidization Involving Interploidy Hybridization

Our results support the long-standing hypothesis that hexaploid *L. multiflora* is of allopolyploid origin (Kirschner 1992), even though the precise identity of its parental species remains somewhat uncertain. The hexaploid cytotype of *L. multiflora* has close genetic affinities to the tetraploid species, and genomic data suggest an origin through interploidy hybridization between the common ancestor of *L. divulgata* and tetraploid *L. multiflora* and diploid *L. campestris* (Fig. 7a). This hypothesis is supported by shared splits with *L. campestris* in the phylogenetic network (Fig. 4b), the position of the hexaploid in the TreeMix analysis (Fig. 5a), signals of weak but significant introgression (Fig. 5b), relatively high relatedness coefficients (Fig. 6a), and partially shared genetic clusters in the STRUCTURE analysis (Supplementary Fig. S10). The elevated relatedness of hexaploid *L. multiflora* to *L. divulgata* (Fig. 6a) compared with tetraploid *L. multiflora* may indicate that the hexaploid originated directly from *L. divulgata*, rather than from the common ancestor of both tetraploids. However, while significant, these differences are small, and our data do not allow inference of the timing of polyploidization events. An origin involving genetically distant *L. campestris* aligns with the observation that the likelihood of polyploidization increases with increasing divergence of parental genomes during hybridization (Paun et al. 2009). Hexaploid *L. multiflora* thus likely constitutes an allopolyploid comprising three distinct subgenomes that arose from hybridization between a diploid and an allotetraploid, similar to *Avena sativa* (Peng et al. 2022) and *Triticum aestivum* (Li et al. 2015).

Like its tetraploid counterpart, the hexaploid cytotype of *L. multiflora* also has genetic affinities (Figs. 5a,b and 6a) with *L. pallescens*, which we, however, interpret as a result of recent (post-polyploidization) introgression. Given that *L. pallescens* is likely a progenitor of the tetraploids and assuming limited evolutionary divergence of polyploids, such introgression would introduce a homologous chromosome set likely to engage in successful meiotic pairing. An alternative scenario *–* where *L. pallescens* served as a parental species of the hexaploid, and the signal of *L. campestris* reflects later introgression *–* appears less plausible, because introgression from genetically distant *L. campestris* would introduce a divergent chromosome set, potentially causing meiotic instability.

Finally, under the alternative hypothesis that *L. divulgata* arose from interploidy hybridization between *L. taurica* and hexaploid *L. multiflora*, the latter could have originated from backcrossing between tetraploid *L. multiflora* and its likely progenitor *L. pallescens* (Fig. 7b). However, this scenario does not account for the genomic contribution of *L. campestris* found in the hexaploid. Together with the limited evidence for diploid–hexaploid hybridization in general (see above), our results support a common origin of tetraploid *L. multiflora* and *L. divulgata*, followed by allopolyploidization with *L. campestris* that gave rise to hexaploid *L. multiflora*, as the most likely scenario.

Although polyploidization is generally associated with strong postzygotic reproductive isolation from diploid progenitors (Levin 2002; Ramsey and Schemske 2002), gene flow across ploidy levels has been documented in diverse plant genera. Such interploidy gene flow can occur with diploid progenitors *–* as observed in autotetraploid *Biscutella laevigata* (Grünig et al. 2024) *–* but may also involve more distantly related, non-parental species, as evidenced in *Arabidopsis* (Jørgensen et al. 2011; Marburger et al. 2019; Monnahan et al. 2019), *Betula* (Ashburner and McAllister 2013; Zohren et al. 2016; Leal et al. 2024), *Miscanthus* (Clark et al. 2015), *Rorippa* (Bleeker and Hurka 2001; Bleeker 2003) and *Triticum* (Cheng et al. 2019). A recent study by Brown et al. (2024) found that 35% of hybrids in the British flora are cross-ploidy hybrids, notably also in Juncaceae, and suggests that interploidy gene flow is often overlooked and may be more common than previously assumed, particularly in allopolyploids.

In *Luzula* sect. *Luzula*, hybridization between diploids and tetraploids has been deemed impossible (Nordenskiöld 1956; Kirschner 1991). However, gene flow between tetra- and hexaploids has been documented (Nordenskiöld 1956; Kirschner 1991), and recent findings suggest that interploidy gene flow does occur at least sporadically in this section, possibly mediated through a “pentaploid bridge” (Ptáček et al. 2023). For instance, putative pentaploids, inferred from relative GS estimation, were reported in mixed-ploidy populations (Geurden et al. 2025). In support of these findings, we identified six putative triploid and three putative pentaploid individuals in a data set of 3462 European *Luzula* sect. *Luzula* accessions based on RGS measurements (Supplementary Fig. S20, Supplementary Table S8).

### Luzula alpina arose through Interploidy Hybridization between L. exspectata and Tetraploid L. multiflora

An allopolyploid origin of *L. alpina* has long been postulated (Kirschner 1992), and our results support this hypothesis, thus rejecting the alternative hypothesis that *L. alpina* is a paraphyletic taxon formed through recurrent partial chromosome fragmentation within *L. multiflora* populations (Pungaršek et al. 2023). Specifically, we identify *L. exspectata* as one of the parental species of *L. alpina*. Contrary to a hypothesis by Bačič et al. (2019), however, *L. taurica* is unlikely to be the second progenitor. Instead, multiple lines of genomic evidence consistently indicate that *L. alpina* originated via interploidy hybridization between reduced gametes of tetraploid *L. multiflora* and unreduced gametes of *L. exspectata* (Fig. 7a,b). This is supported by patterns of genetic admixture (Fig. 4d), the intermediate position of *L. alpina* between *L. exspectata* and tetraploid *L. multiflora* in the PCA (Fig. 4e), strong signatures of introgression (Fig. 5a,b), and high pairwise relatedness to both parental species (Fig. 6a).

At a first glance, such interploidy hybridization may seem unlikely, given the distinct karyotypes of the parental taxa: *L. exspectata* is agmatoploid with 24 fragmented BL chromosomes, whereas *L. multiflora* has 24 full-sized AL chromosomes. However, in *Luzula*, AL- and BL-type chromosomes are known to readily pair during meiosis (Nordenskiöld 1961), and a hybrid origin from these two species matches the 12AL + 24BL karyotype of *L. alpina*. The low genetic divergence between *L. exspectata* and *L. taurica –* a likely progenitor of *L. multiflora –* further supports the possibility of successful chromosome pairing and hybrid viability. In addition, a close relationship between *L. alpina* and tetraploid *L. multiflora* from the Alps is also supported by their ability to hybridize and produce fertile offspring (Nordenskiöld 1956), which likely accounts for the presence of admixed individuals, particularly in the contact zone of both species (Fig. 3d).

Finally, the presence of both *L. multiflora* and *L. exspectata* plastid haplotypes in *L. alpina* suggests two independent origins of the species: one involving *L. multiflora* as the maternal progenitor, constituting the majority of populations, and a secondary origin with *L. exspectata* as the maternal parent that probably occurred in the northwestern region of the Eastern Alps (Supplementary Figs. S18 and S19). Although two independent origins may partially explain the substantial genetic substructure observed within *L. alpina* (Fig. 4b, Supplementary Fig. S9), they do not fully account for it, as the distribution of plastid haplotypes and nuclear genetic structure are not entirely congruent (Supplementary Fig. S21).

### Reduced Recombination Rates in Holocentrics and Adaptive Introgression may assist the Establishment of Polyploids

Meiotic chromosome segregation poses a major challenge for newly formed polyploids during their establishment (Comai 2005; Baduel et al. 2018; Bomblies 2023) and reduced fertility and aneuploidy in new polyploids are often associated with the formation of multivalents during meiosis (Ramsey and Schemske 2002; Lloyd and Bomblies 2016). Although multivalents are more common in autopolyploids due to the lack of subgenome divergence (Bomblies 2023), they also occur in allopolyploids, albeit less frequently (Loidl et al. 1990; Grandont et al. 2014; Lloyd and Bomblies 2016). In contrast to established allopolyploids, neo-allopolyploids often exhibit higher rates of multivalent formation (Lloyd and Bomblies 2016), leading to increased rates of homeologous exchange, as observed in *Brassica napus* (Szadkowski et al. 2010; Chalhoub et al. 2014), *Arabidopsis suecica* (Henry et al. 2014; Jiang et al. 2021), *Oryza sativa* (Xu et al. 2014; Wu et al. 2021), and *Tragopogon miscellus* (Chester et al. 2012). Homeologous recombination can destabilize polyploid genomes by homogenizing divergent subgenomes, promoting aneuploidy, and causing gene loss (Feldman and Levy 2009; Zhang et al. 2013). One way to improve meiotic stability and to reduce the prevalence of multivalents and homeologous recombination is a reduced crossover rate (Carvalho et al. 2010; Yant et al. 2013). In fact, having only a single crossover event per chromosome prevents multivalent formation completely (Lloyd and Bomblies 2016).

Holocentric chromosomes, as found in *Luzula*, typically exhibit low crossover rates, usually limited to one or two crossovers per chromosome (Nordenskiöld 1962; Nokkala et al. 2004; Martinez-Perez et al. 2008; Heckmann et al. 2014). This suggests the intriguing possibility that reduced recombination rates may facilitate polyploid establishment in *Luzula*, consistent with observations that diploids with low crossover frequencies tend to give rise to more fertile tetraploids (Lavania 1991; Srivastava et al. 1992). However, polyploidy is conspicuously rare in the megadiverse holocentric genus *Carex* (Hipp et al. 2009; Lipnerova et al. 2013). Although it occurs more frequently in other Cyperaceae (Vanzela et al. 2000; Yano and Hoshino 2005), chromosome fragmentations and fusions rather than WGDs seem to be the predominant drivers behind the high karyotypic diversity of *Carex* and other holocentrics (Kondo and Lavarack 1984; Hipp et al. 2009; Escudero et al. 2024).

Improved meiotic stability may also be obtained via introgression of adaptive alleles, which can reduce multivalent formation. This is well documented in the case of bidirectional gene flow between the autotetraploids *Arabidopsis arenosa* and *A. lyrata* (Yant et al. 2013; Marburger et al. 2019), and also in the allotetraploid *A. suecica*, which carries beneficial meiotic alleles from diploid *A. arenosa* (Nibau et al. 2022). Likewise, introgression from diploids into polyploids has recently been evidenced in birches (Leal et al. 2024). Importantly, in lineages with complex reticulate evolution, such post-polyploidization introgression can be difficult to distinguish from genomic signals associated with the original polyploidization event (Mandel et al. 2017; Wagner et al. 2020; Wang et al. 2021; Leal et al. 2024).

A similar scenario may be plausible for the European members of *Luzula* sect. *Luzula*. As an alternative to the allopolyploid origin proposed above, the complex genomic signals observed in *L. divulgata* may reflect an ancient autopolyploid origin from *L. taurica* followed by gradual diploidization and a transition toward disomic inheritance (Parisod et al. 2010), which might explain the ambiguous results regarding the inheritance mode of *L. divulgata* (Fig. 4a, Supplementary Figs. S6b and 7c). Extensive post-polyploidization introgression from both *L. pallescens* and hexaploid *L. multiflora* might be misinterpreted as a signature of allopolyploidy, similar to the case of *Betula pubescens* (Leal et al. 2024). These three *Luzula* species frequently co-occur, and hybridization between tetra- and hexaploids has been documented (Kirschner 1991). Such a scenario would agree with known mechanisms of meiotic stabilization through introgression and could explain the conflicting genomic signatures found in *L. divulgata* without invoking an allopolyploid origin.

### Scope and Taxonomic Implications

Genomic evidence points to a central role of *L. pallescens* in the formation of the studied polyploids, supporting earlier conjectures (Kirschner 1992), yet the precise evolutionary history of *Luzula* sect. *Luzula* remains to be elucidated. Although our sampling is broad, it does not cover the entire geographic distribution and taxonomic diversity of the section. This is particularly relevant for *L. multiflora*, a widespread species with numerous subspecies across the northern hemisphere (Kirschner 2002), which may involve unsampled progenitors in its origin. For the other polyploids studied, such contributions appear less likely given their more restricted ranges. Nonetheless, involvement of extinct lineages remains a plausible explanation for the limited plastid haplotype sharing observed between polyploids and diploids (Supplementary Fig. S19). Such contributions from extinct “ghost lineages” have been evidenced in numerous other plant groups (Kamneva et al. 2017; Wei et al. 2017; Sancho et al. 2022; Španiel et al. 2023) and seem especially pertinent in the case of hexaploid *L. multiflora*.

Although not conclusive, our results challenge the current taxonomic circumscription of *Luzula* sect. *Luzula*. The lack of genetic differentiation between the diploids *L. divulgatiformis* and *L. exspectata*, confirming results of Carrizo García et al. (2025), together with their shared karyotype, questions their status as separate species. Their distinct ecological niches *–* calcareous lowland meadows and open woodlands, and (sub)alpine limestone grasslands, respectively *–* suggest they may represent ecotypes of a single, more broadly distributed species rather than taxonomically distinct lineages. Similarly, treating the tetraploid and the hexaploid cytotypes of *L. multiflora* as the same species defies genetic evidence. We, therefore, suggest treating the allohexaploid *L. multiflora* as a distinct species but acknowledge that a revised taxonomic treatment of the group goes beyond the scope of this study.

## Conclusions

Our results suggest a complex evolutionary history of European *Luzula* sect. *Luzula* that involved multiple polyploidization and hybridization events. The tetraploids *L. divulgata* and *L. multiflora* emerge as the oldest polyploid lineages within the group, likely originating from a common ancestor formed through allopolyploidization from *L. taurica* and *L. pallescens*. This common ancestor may then have hybridized with diploid *L. campestris*, giving rise to hexaploid *L. multiflora* in a second allopolyploidization event. Finally, tetraploid *L. multiflora* appears ancestral to *L. alpina*, which arose via interploidy hybridization with *L. exspectata*. The shared major haplotypes across all polyploids (Supplementary Figs. S18 and S19) indicate that they likely served as maternal progenitors in the proposed hybridization events. This is consistent with the general phenomenon that interploidy crosses are more likely to produce viable offspring when the maternal parent has higher ploidy, due to a better tolerated ratio of parental contributions to the endosperm (Burton and Husband 2000; Josefsson et al. 2006). Strong signals of admixture in *L. alpina* support its comparatively younger age, consistent with earlier hypotheses (Nordenskiöld 1956), and imply ongoing gene flow with its progenitors. Taken together, these findings reveal reticulate species relationships and highlight a rare case of hybrid speciation across ploidy barriers. To our knowledge, *L. alpina* is the only example where such interploidy hybridization involves chromosomes of different sizes, showcasing the holocentric genus *Luzula* as a unique system for investigating the evolutionary consequences of agmatoploidy and polyploidy.

In addition to the inherent biological complexity of the group, methodological limitations of our study may also hinder conclusive inference of the evolutionary history of *Luzula* sect. *Luzula*. Although plastid markers have successfully disentangled reticulate relationships when combined with biparentally inherited low-copy nuclear genes (Frajman et al. 2009; Marcussen et al. 2015; Wang et al. 2021), the plastid markers used in this study fail to resolve species relationships within *Luzula* sect. *Luzula*. This is likely related to the young age of *Luzula* sect. *Luzula*, which originated in the Pliocene and started to diversify in the Pleistocene (Carrizo García et al. 2025), and differential sorting of ancestral polymorphisms, but also ongoing gene flow. Sharing of plastid haplotypes across species is common in wind-pollinated species and thus hardly surprising in *Luzula* (Morrone et al. 2012; Kuzmanović et al. 2017). On the other hand, the plastid markers used here offer enough resolution for phylogenetic inference among more distantly related *Luzula* species from other sections (Carrizo García et al. 2025).

Finally, RADseq data, despite providing greatly improved resolution compared with previous ITS and plastid phylogenies (Drábková et al. 2006; Záveská Drábková and Vlček 2010), are not ideal for inferring phylogenetic relationships, particularly in allopolyploid complexes. Genomic data derived from targeted sequencing of low-copy nuclear genes (Kamneva et al. 2017), transcriptome sequencing (Yang et al. 2023), or whole-genome sequencing (WGS; Kryvokhyzha et al. 2019) will open the door for applying more sophisticated approaches that allow phasing of homeologs and appropriately model allopolyploid networks (Jones et al. 2013; Freyman et al. 2023; Tiley et al. 2024; Kong et al. 2025). Also, the genomic polarization approach adapted here would prove more effective when applied to target-capture or WGS sequence data (Leal et al. 2023). Ultimately, combining these techniques with a more complete taxon sampling could finally enable researchers to disentangle the complex yet exciting evolutionary history of the notoriously challenging genus *Luzula*.

## Supplementary Material

syaf065_Supplemental_Files

## Data Availability

Data are deposited at the Dryad Digital Repository: https://doi.org/10.5061/dryad.gb5mkkx2p. Code used for bioinformatic analyses can be accessed from Zenodo: https://doi.org/10.5281/zenodo.15719018. Raw sequence reads are publicly available at NCBI under BioProjects PRJNA1313421 and PRJNA1225458. GenBank accession numbers for plastid sequences are provided in Supplementary Table S1.

## References

[bib1] Abbott R., Albach D., Ansell S., Arntzen J.W., Baird S.J.E., Bierne N., Boughman J., Brelsford A., Buerkle C.A., Buggs R., Butlin R.K., Dieckmann U., Eroukhmanoff F., Grill A., Cahan S.H., Hermansen J.S., Hewitt G., Hudson A.G., Jiggins C., Jones J., Keller B., Marczewski T., Mallet J., Martinez‐Rodriguez P., Möst M., Mullen S., Nichols R., Nolte A.W., Parisod C., Pfennig K., Rice A.M., Ritchie M.G., Seifert B., Smadja C.M., Stelkens R., Szymura J.M., Väinölä R., Wolf J.B.W., Zinner D. 2013. Hybridization and speciation. J. Evol. Biol. 26(2):229–246. 23323997 10.1111/j.1420-9101.2012.02599.x

[bib2] Andrews S. 2010. FASTQC: a quality control tool for high throughput sequence data. http://www.bioinformatics.babraham.ac.uk/projects/fastqc.

[bib3] Arnold B., Kim S.-T., Bomblies K. 2015. Single geographic origin of a widespread autotetraploid *Arabidopsis arenosa* lineage followed by interploidy admixture. Mol. Biol. Evol. 32(6):1382–1395. 25862142 10.1093/molbev/msv089

[bib4] Arnold M.L. 1993. *Iris nelsonii* (Iridaceae): origin and genetic composition of a homoploid hybrid species. Am. J. Bot. 80(5):577–583. 30139150 10.1002/j.1537-2197.1993.tb13843.x

[bib5] Asay K.H., Dewey D.R. 1979. Bridging ploidy differences in crested wheatgrass with hexaploid ✕ diploid hybrids. Crop Sci. 19(4):519–523.

[bib6] Ashburner K., McAllister H.A. 2013. The genus Betula: a taxonomic revision of birches. London: Kew Publishing.

[bib7] Bačič T., Dolenc Koce J., Frajman B. 2019. Diversification and distribution patterns of *Luzula* sect. *Luzula* (Juncaceae) in the Eastern Alps: a cytogenetic approach combined with extensive herbarium revisions. Alp. Bot. 129:149–161.

[bib8] Bačič T., Frajman B., Dolenc Koce J. 2016. Diversification of *Luzula* sect. *Luzula* (Juncaceae) on the Balkan Peninsula—a cytogenetic approach. Folia Geobot. 51:51–63.

[bib9] Bačič T., Jogan N., Dolenc Koce J. 2007a. *Luzula* sect. *Luzula* in the south-eastern Alps—karyology and genome size. Taxon. 56:129–136.

[bib10] Bačič T., Koce J.D., Jogan N. 2007b. *Luzula* sect. *Luzula* (Juncaceae) in the South-Eastern Alps: morphology, determination and geographic distribution. Bot. Helvetica. 117:75–88.

[bib11] Baduel P., Bray S., Vallejo-Marin M., Kolář F., Yant L. 2018. The “Polyploid Hop”: shifting challenges and opportunities over the evolutionary lifespan of genome duplications. Front. Ecol. Evol. 6:117.

[bib12] Bayona-Vásquez N.J., Glenn T.C., Kieran T.J., Pierson T.W., Hoffberg S.L., Scott P.A., Bentley K.E., Finger J.W., Louha S., Troendle N., Diaz-Jaimes P., Mauricio R., Faircloth B.C. 2019. Adapterama III: quadruple-indexed, double/triple-enzyme RADseq libraries (2RAD/3RAD). PeerJ. 7:e7724. 31616583 10.7717/peerj.7724 PMC6791345

[bib13] Bleeker W. 2003. Hybridization and *Rorippa austriaca* (Brassicaceae) invasion in Germany. Mol. Ecol. 12(7):1831–1841. 12803635 10.1046/j.1365-294x.2003.01854.x

[bib14] Bleeker W., Hurka H. 2001. Introgressive hybridization in *Rorippa* (Brassicaceae): gene flow and its consequences in natural and anthropogenic habitats. Mol. Ecol. 10(8):2013–2022. 11555244 10.1046/j.1365-294x.2001.01341.x

[bib15] Bolger A.M., Lohse M., Usadel B. 2014. Trimmomatic: a flexible trimmer for Illumina sequence data. Bioinformatics. 30(15):2114–2120. 24695404 10.1093/bioinformatics/btu170 PMC4103590

[bib16] Bomblies K. 2023. Learning to tango with four (or more): the molecular basis of adaptation to polyploid meiosis. Plant Reprod. 36(1):107–124. 36149479 10.1007/s00497-022-00448-1 PMC9957869

[bib17] Bouckaert R.R. 2010, DensiTree: making sense of sets of phylogenetic trees, Bioinformatics. 26(10):1372–1373. 20228129 10.1093/bioinformatics/btq110

[bib18] Bouckaert R.R., Vaughan T.G., Barido-Sottani J., Duchêne S., Fourment M., Gavryushkina A., Heled J., Jones G., Kühnert D., De Maio N., Matschiner M., Mendes F.K., Müller N.F., Ogilvie H.A., Du Plessis L., Popinga A., Rambaut A., Rasmussen D., Siveroni I., Suchard M.A., Wu C.-H., Xie D., Zhang C., Stadler T., Drummond A.J. 2019. BEAST 2.5: an advanced software platform for Bayesian evolutionary analysis. PLoS Comput. Biol. 15(4):e1006650. 30958812 10.1371/journal.pcbi.1006650 PMC6472827

[bib19] Bozek M., Leitch A.R., Leitch I.J., Záveská Drábková L., Kuta E. 2012. Chromosome and genome size variation in *Luzula* (Juncaceae), a genus with holocentric chromosomes: chromosome and C-Value Evolution in *L uzula*. Bot J Linn Soc. 170(4):529–541.

[bib20] Brandrud M.K., Baar J., Lorenzo M.T., Athanasiadis A., Bateman R.M., Chase M.W., Hedrén M., Paun O. 2020. Phylogenomic relationships of diploids and the origins of allotetraploids in *Dactylorhiza* (Orchidaceae). Syst. Biol. 69(1):91–109. 31127939 10.1093/sysbio/syz035 PMC6902629

[bib21] Brown M.R., Abbott R.J., Twyford A.D. 2024. The emerging importance of cross-ploidy hybridisation and introgression. Mol. Ecol. 33(8):e17315. 38501394 10.1111/mec.17315

[bib22] Brožová V., Proćków J., Záveská Drábková L. 2022. Toward finally unraveling the phylogenetic relationships of Juncaceae with respect to another cyperid family, Cyperaceae. Mol. Phylogenet. Evol. 177:107588. 35907594 10.1016/j.ympev.2022.107588

[bib23] Bryant D., Bouckaert R.R., Felsenstein J., Rosenberg N.A., RoyChoudhury A. 2012. Inferring species trees directly from biallelic genetic markers: bypassing gene trees in a full coalescent analysis. Mol. Biol. Evol. 29(8):1917–1932. 22422763 10.1093/molbev/mss086 PMC3408069

[bib24] Bureš P., Zedek F., Marková M. 2013. Holocentric chromosomes. In: Greilhuber J., Dolezel J., Wendel J.F., editors. Plant genome diversity, Vol. 2. Vienna: Springer. p. 187–208.

[bib25] Burton T.L., Husband B.C. 2000. Fitness differences among diploids, tetraploids, and their triploid progeny in *Chamerion angustifolium*: mechanisms of inviability and implications for polyploid evolution. Evolution. 54:1182–1191. 11005287 10.1111/j.0014-3820.2000.tb00553.x

[bib26] Carrizo García C., Heimer V., Schönswetter P., Varotto C., Frajman B., Li M. 2025. Contrasting diversification patterns across wood rushes from *Luzula* sect. *Luzula* (Juncaceae) revealed by 3RAD genome-wide sequencing. Mol. Phylogenet. Evol. 214:108455. 40907588 10.1016/j.ympev.2025.108455

[bib27] Carvalho A., Delgado M., Barão A., Frescatada M., Ribeiro E., Pikaard C.S., Viegas W., Neves N. 2010. Chromosome and DNA methylation dynamics during meiosis in the autotetraploid *Arabidopsis arenosa*. Sex Plant Reprod. 23(1):29–37. 20165961 10.1007/s00497-009-0115-2

[bib28] Castro S., Loureiro J., Procházka T., Münzbergová Z. 2012. Cytotype distribution at a diploid–hexaploid contact zone in *Aster amellus* (Asteraceae). Ann. Bot. 110(5):1047–1055. 22887024 10.1093/aob/mcs177 PMC3448430

[bib29] Castro S., Münzbergová Z., Raabová J., Loureiro J. 2011. Breeding barriers at a diploid–hexaploid contact zone in *Aster amellus*. Evol Ecol. 25(4):795–814.

[bib30] Catchen J., Hohenlohe P.A., Bassham S., Amores A., Cresko W.A. 2013. Stacks: an analysis tool set for population genomics. Mol. Ecol. 22(11):3124–3140. 23701397 10.1111/mec.12354 PMC3936987

[bib31] Chalhoub B., Denoeud F., Liu S., Parkin I.A.P., Tang H., Wang X., Chiquet J., Belcram H., Tong C., Samans B., Corréa M., Da Silva C., Just J., Falentin C., Koh C.S., Le Clainche I., Bernard M., Bento P., Noel B., Labadie K., Alberti A., Charles M., Arnaud D., Guo H., Daviaud C., Alamery S., Jabbari K., Zhao M., Edger P.P., Chelaifa H., Tack D., Lassalle G., Mestiri I., Schnel N., Le Paslier M.-C., Fan G., Renault V., Bayer P.E., Golicz A.A., Manoli S., Lee T.-H., Thi V.H.D., Chalabi S., Hu Q., Fan C., Tollenaere R., Lu Y., Battail C., Shen J., Sidebottom C.H.D., Wang X., Canaguier A., Chauveau A., Bérard A., Deniot G., Guan M., Liu Z., Sun F., Lim Y.P., Lyons E., Town C.D., Bancroft I., Wang X., Meng J., Ma J., Pires J.C., King G.J., Brunel D., Delourme R., Renard M., Aury J.-M., Adams K.L., Batley J., Snowdon R.J., Tost J., Edwards D., Zhou Y., Hua W., Sharpe A.G., Paterson A.H., Guan C., Wincker P. 2014. Early allopolyploid evolution in the post-Neolithic *Brassica napus* oilseed genome. Science. 345(6199):950–953. 25146293 10.1126/science.1253435

[bib32] Cheng H., Liu J., Wen J., Nie X., Xu L., Chen N., Li Z., Wang Q., Zheng Z., Li M., Cui L., Liu Z., Bian J., Wang Z., Xu S., Yang Q., Appels R., Han D., Song W., Sun Q., Jiang Y. 2019. Frequent intra- and inter-species introgression shapes the landscape of genetic variation in bread wheat. Genome Biol. 20(1):136. 31300020 10.1186/s13059-019-1744-x PMC6624984

[bib33] Chester M., Gallagher J.P., Symonds V.V., Cruz Da Silva A.V., Mavrodiev E.V., Leitch A.R., Soltis P.S., Soltis D.E. 2012. Extensive chromosomal variation in a recently formed natural allopolyploid species, *Tragopogon miscellus* (Asteraceae). Proc. Natl. Acad. Sci. USA. 109(4):1176–1181. 22228301 10.1073/pnas.1112041109 PMC3268322

[bib34] Clark L.V., Stewart J.R., Nishiwaki A., Toma Y., Kjeldsen J.B., Jørgensen U., Zhao H., Peng J., Yoo J.H., Heo K., Yu C.Y., Yamada T., Sacks E.J. 2015. Genetic structure of *Miscanthus sinensis* and *Miscanthus sacchariflorus* in Japan indicates a gradient of bidirectional but asymmetric introgression. EXBOTJ. 66(14):4213–4225. 10.1093/jxb/eru511 PMC4493777 25618143

[bib35] Clement M., Posada D., Crandall K.A. 2000. TCS: a computer program to estimate gene genealogies. Mol. Ecol. 9(10):1657–1659. 11050560 10.1046/j.1365-294x.2000.01020.x

[bib36] Comai L. 2005. The advantages and disadvantages of being polyploid. Nat. Rev. Genet. 6(11):836–846. 16304599 10.1038/nrg1711

[bib37] Danecek P., Bonfield J.K., Liddle J., Marshall J., Ohan V., Pollard M.O., Whitwham A., Keane T., McCarthy S.A., Davies R.M., Li H. 2021. Twelve years of SAMtools and BCFtools. GigaScience. 10(2):giab008. 33590861 10.1093/gigascience/giab008 PMC7931819

[bib38] Debray K., Le Paslier M.-C., Bérard A., Thouroude T., Michel G., Marie-Magdelaine J., Bruneau A., Foucher F., Malécot V. 2022. Unveiling the patterns of reticulated evolutionary processes with phylogenomics: hybridization and polyploidy in the genus *Rosa*. Syst. Biol. 71(3):547–569. 34329460 10.1093/sysbio/syab064

[bib39] Degnan J.H. 2013. Anomalous unrooted gene trees. Syst. Biol. 62(4):574–590. 23576318 10.1093/sysbio/syt023

[bib40] Dewey D.R. 1973. Hybrids between diploid and hexaploid crested wheatgrass. Crop Sci. 13(4):474–477.

[bib41] Drábková L., Kirschner J., Vlček Č. 2006. Phylogenetic relationships within *Luzula* DC. and *Juncus* L. (Juncaceae): a comparison of phylogenetic signals of *trn* L- *trn* F intergenic spacer, *trn* L intron and *rbc* L plastome sequence data. Cladistics. 22(2):132–143. 34892869 10.1111/j.1096-0031.2006.00095.x

[bib42] Drummond A.J., Bouckaert R.R. 2015. Bayesian evolutionary analysis with BEAST. Cambridge: Cambridge University Press.

[bib43] Edger P.P., McKain M.R., Bird K.A., VanBuren R. 2018. Subgenome assignment in allopolyploids: challenges and future directions. Curr. Opin. Plant Biol. 42:76–80. 29649616 10.1016/j.pbi.2018.03.006

[bib44] Escudero M., Marques A., Lucek K., Hipp A.L. 2024. Genomic hotspots of chromosome rearrangements explain conserved synteny despite high rates of chromosome evolution in a holocentric lineage. Mol. Ecol. 33(24):e17086. 37486041 10.1111/mec.17086 PMC11628656

[bib45] Escudero M., Márquez-Corro J.I., Hipp A.L. 2016. The phylogenetic origins and evolutionary history of holocentric chromosomes. Systematic Botany. 41(3):580–585.

[bib46] Evanno G., Regnaut S., Goudet J. 2005. Detecting the number of clusters of individuals using the software structure: a simulation study. Mol. Ecol. 14(8):2611–2620. 15969739 10.1111/j.1365-294X.2005.02553.x

[bib47] Feldman M., Levy A.A. 2009. Genome evolution in allopolyploid wheat—a revolutionary reprogramming followed by gradual changes. Journal of Genetics and Genomics. 36(9):511–518. 19782952 10.1016/S1673-8527(08)60142-3

[bib48] Fischer M., Oswald K., Adler W. 2008. Exkursionsflora für Österreich, Liechtenstein und Südtirol, 3rd edn. Oberösterreichische Landesmuseen, Linz.

[bib49] Fitak R.R. 2021. *OptM*: estimating the optimal number of migration edges on population trees using *Treemix*. Biol. Methods Protoc. 6(1):bpab017. 34595352 10.1093/biomethods/bpab017 PMC8476930

[bib50] Frajman B., Eggens F., Oxelman B. 2009. Hybrid origins and homoploid reticulate evolution within *Heliosperma* (Sileneae, Caryophyllaceae)—a multigene phylogenetic approach with relative dating, Syst. Biol. 58(3):328–345. 20525587 10.1093/sysbio/syp030

[bib51] Francis R.M. 2017. pophelper: an R package and web app to analyse and visualize population structure. Mol. Ecol. Resour. 17(1):27–32. 26850166 10.1111/1755-0998.12509

[bib52] Frankel L.E., Ané C. 2023. Summary tests of introgression are highly sensitive to rate variation across lineages. Syst. Biol. 67:901–904. 10.1093/sysbio/syad056 37698548

[bib53] Frankel L.E., Ané C. 2025. Low accuracy of complex admixture graph inference from *f*-statistics. Preprint available at bioRxiv: doi: 10.1101/2025.03.07.642126. 41330409

[bib54] Freyman W.A., Johnson M.G., Rothfels C.J. 2023. homologizer: phylogenetic phasing of gene copies into polyploid subgenomes. Methods Ecol. Evol. 14(5):1230–1244. 10.1007/978-1-0716-2561-3_6 36720810

[bib55] Geurden J., Heimer V., Frajman B. 2025. Co-occurring *Luzula* species (Juncaceae) of different ploidies exhibit weak ecological differentiation at local scale in alpine grasslands of the Eastern Alps. Alp Botany. 135:275–288.

[bib56] Goodwin Z.A., Bell D., Hart M.L., Hollingsworth P.M., Royal Botanic Garden Edinburgh Genome Acquisition Lab, Plant Genome Sizing collective, Darwin Tree of Life Barcoding collective, Wellcome Sanger Institute Tree of Life Management, Samples and Laboratory team, Wellcome Sanger Institute Scientific Operations: Sequencing Operations, Wellcome Sanger Institute Tree of Life Core Informatics team, Tree of Life Core Informatics collective, Darwin Tree of Life Consortium. 2024. The genome sequence of great wood-rush, *Luzula sylvatica* (Huds) Gaudin. Wellcome Open Res. 9:124. 39246514 10.12688/wellcomeopenres.20997.1 PMC11380069

[bib57] Grandont L., Cuñado N., Coriton O., Huteau V., Eber F., Chèvre A.M., Grelon M., Chelysheva L., Jenczewski E. 2014. Homoeologous chromosome sorting and progression of meiotic recombination in *Brassica napus*: ploidy does matter!. Plant Cell. 26(4):1448–1463. 24737673 10.1105/tpc.114.122788 PMC4036564

[bib58] Grant P.R., Grant B.R. 2020. Triad hybridization via a conduit species. Proc. Natl. Acad. Sci. USA. 117(14):7888–7896. 32213581 10.1073/pnas.2000388117 PMC7148562

[bib59] Green R.E., Krause J., Briggs A.W., Maricic T., Stenzel U., Kircher M., Patterson N., Li H., Zhai W., Fritz M.H.-Y., Hansen N.F., Durand E.Y., Malaspinas A.-S., Jensen J.D., Marques-Bonet T., Alkan C., Prüfer K., Meyer M., Burbano H.A., Good J.M., Schultz R., Aximu-Petri A., Butthof A., Höber B., Höffner B., Siegemund M., Weihmann A., Nusbaum C., Lander E.S., Russ C., Novod N., Affourtit J., Egholm M., Verna C., Rudan P., Brajkovic D., Kucan Ž., Gušic I., Doronichev V.B., Golovanova L.V., Lalueza-Fox C., De La Rasilla M., Fortea J., Rosas A., Schmitz R.W., Johnson P.L.F., Eichler E.E., Falush D., Birney E., Mullikin J.C., Slatkin M., Nielsen R., Kelso J., Lachmann M., Reich D., Pääbo S. 2010. A draft sequence of the Neandertal genome. Science. 328(5979):710–722. 20448178 10.1126/science.1188021 PMC5100745

[bib60] Grünig S., Patsiou T., Parisod C. 2024. Ice age-driven range shifts of diploids and expanding autotetraploids of *Biscutella laevigata* within a conserved niche. New Phytol. 244:1616–1628. 39253771 10.1111/nph.20103

[bib61] Guerra M. 2016. Agmatoploidy and symploidy: a critical review. Genet. Mol. Biol. 39(4):492–496. 27791217 10.1590/1678-4685-GMB-2016-0103 PMC5127162

[bib62] Guo Y., Tong X., Wang L., Vogl C. 2013. A population genetic model to infer allotetraploid speciation and long-term evolution applied to two yarrow species. New Phytol. 199(2):609–621. 23574432 10.1111/nph.12262

[bib63] Haizel T., Lim Y.K., Leitch A.R., Moore G. 2005. Molecular analysis of holocentric centromeres of *Luzula* species. Cytogenet. Genome Res. 109(1-3):134–143. 15753569 10.1159/000082392

[bib64] Heckmann S., Jankowska M., Schubert V., Kumke K., Ma W., Houben A. 2014. Alternative meiotic chromatid segregation in the holocentric plant *Luzula* elegans. Nat. Commun. 5(1):4979. 25296379 10.1038/ncomms5979 PMC4214429

[bib65] Hegarty M.J., Abbott R.J., Hiscock S.J. 2012. Allopolyploid speciation in action: the origins and evolution of *Senecio cambrensis*. In: Soltis P.S., Soltis D.E., editors. Polyploidy and genome evolution. Berlin: Springer. p. 245–270.

[bib66] Henry I.M., Dilkes B.P., Tyagi A., Gao J., Christensen B., Comai L. 2014. The BOY NAMED SUE quantitative trait locus confers increased meiotic stability to an adapted natural allopolyploid of *Arabidopsis*. Plant Cell. 26(1):181–194. 24464296 10.1105/tpc.113.120626 PMC3963567

[bib67] Hipp A.L., Rothrock P.E., Roalson E.H. 2009. The evolution of chromosome arrangements in *Carex* (Cyperaceae). Bot. Rev. 75(1):96–109.

[bib68] Hoang D.T., Chernomor O., Von Haeseler A., Minh B.Q., Vinh L.S. 2018. UFBoot2: improving the ultrafast bootstrap approximation. Mol. Biol. Evol. 35(2):518–522. 29077904 10.1093/molbev/msx281 PMC5850222

[bib69] Huang K., Ritland K., Guo S., Shattuck M., Li B. 2014. A pairwise relatedness estimator for polyploids. Mol. Ecol. Resour. 14(4):734–744. 24460904 10.1111/1755-0998.12217

[bib70] Husband B.C., Baldwin S.J., Suda J. 2013. The incidence of polyploidy in natural plant populations: major patterns and evolutionary processes. In: Greilhuber J., Dolezel J., Wendel J.F., editors. Plant genome diversity volume 2. Vienna: Springer. p. 255–276.

[bib71] Huson D.H., Bryant D. 2024. The SplitsTree App: interactive analysis and visualization using phylogenetic trees and networks. Nat. Methods. 21(10):1773–1774. 39223398 10.1038/s41592-024-02406-3

[bib72] Ingram R., Noltie H.J. 1995. *Senecio cambrensis* Rosser. The Journal of Ecology. 83(3):537.

[bib73] Jarolímová V., Kirschner J. 1995. Tetraploids in *Luzula multiflora* (Juncaceae) in Ireland: karyology and meiotic behaviour. Folia Geobot. 30:389–396.

[bib74] Jiang X., Song Q., Ye W., Chen Z.J. 2021. Concerted genomic and epigenomic changes accompany stabilization of *Arabidopsis* allopolyploids. Nat. Ecol. Evol. 5(10):1382–1393. 34413505 10.1038/s41559-021-01523-y PMC8484014

[bib75] Jombart T., Ahmed I. 2011. *Adegenet 1.3-1*: new tools for the analysis of genome-wide SNP data. Bioinformatics. 27(21):3070–3071. 21926124 10.1093/bioinformatics/btr521 PMC3198581

[bib76] Jones G., Sagitov S., Oxelman B. 2013. Statistical inference of allopolyploid species networks in the presence of incomplete lineage sorting. Syst. Biol. 62(3):467–478. 23427289 10.1093/sysbio/syt012

[bib77] Jørgensen M.H., Ehrich D., Schmickl R., Koch M.A., Brysting A.K. 2011. Interspecific and interploidal gene flow in Central European *Arabidopsis* (Brassicaceae). BMC Evol. Biol. 11(1):346. 22126410 10.1186/1471-2148-11-346 PMC3247304

[bib78] Josefsson C., Dilkes B., Comai L. 2006. Parent-dependent loss of gene silencing during interspecies hybridization. Curr. Biol. 16(13):1322–1328. 16824920 10.1016/j.cub.2006.05.045

[bib79] Kalyaanamoorthy S., Minh B.Q., Wong T.K.F., Von Haeseler A., Jermiin L.S. 2017. ModelFinder: fast model selection for accurate phylogenetic estimates. Nat. Methods. 14(6):587–589. 28481363 10.1038/nmeth.4285 PMC5453245

[bib80] Kamneva O.K., Syring J., Liston A., Rosenberg N.A. 2017. Evaluating allopolyploid origins in strawberries (*Fragaria*) using haplotypes generated from target capture sequencing. BMC Evol. Biol. 17(1):180. 28778145 10.1186/s12862-017-1019-7 PMC5543553

[bib81] Kearse M., Moir R., Wilson A., Stones-Havas S., Cheung M., Sturrock S., Buxton S., Cooper A., Markowitz S., Duran C., Thierer T., Ashton B., Meintjes P., Drummond A. 2012. Geneious Basic: an integrated and extendable desktop software platform for the organization and analysis of sequence data. Bioinformatics. 28(12):1647–1649. 22543367 10.1093/bioinformatics/bts199 PMC3371832

[bib82] Kirschner J. 1991. An account of natural hybridization within *Luzula* sect. *Luzula* (Juncaceae) in Europe. Preslia. 63:81–112.

[bib83] Kirschner J. 1992. Karyological differentiation of *Luzula* sect. *Luzula* in Europe. Thaiszia. 2:11–39.

[bib84] Kirschner J. 1995. Allozyme analysis of *Luzula* sect. *Luzula* (Juncaceae) in Ireland: evidence of the origin of tetraploids. Folia Geobot. Phytotax. 30(3):283–290.

[bib85] Kirschner J. 1996. *Luzula* sect. *Luzula* (Juncaceae) in Spain. Plant Syst. Evol. 200(1–2):1–11.

[bib86] Kirschner J. , editor. 2002. Luzula. In: Juncaeceae 1: Rostkovia to *Luzula*. Species plantarum: flora of the world part 6. Canberra: National Library of Australia. p. 18–188.

[bib87] Kirschner J., Engelskjoen T., Knaben G.S. 1988. *Luzula alpina* Hoppe, a neglected Alpine species. Preslia. 60:97–108.

[bib88] Kondo K., Lavarack P.S. 1984. A cytotaxonomic study of some Australian species of *Drosera* L. (Droseraceae). Bot. J. Linn. Soc. 88(4):317–333.

[bib89] Kong S., Swofford D.L., Kubatko L.S. 2025. Inference of phylogenetic networks from sequence data using composite likelihood. Syst. Biol. 74(1):53–69. 39387633 10.1093/sysbio/syae054

[bib90] Kopelman N.M., Mayzel J., Jakobsson M., Rosenberg N.A., Mayrose I. 2015. Clumpak: a program for identifying clustering modes and packaging population structure inferences across *K*. Mol. Ecol. Resour. 15(5):1179–1191. 25684545 10.1111/1755-0998.12387 PMC4534335

[bib91] Kryvokhyzha D., Salcedo A., Eriksson M.C., Duan T., Tawari N., Chen J., Guerrina M., Kreiner J.M., Kent T.V., Lagercrantz U., Stinchcombe J.R., Glémin S., Wright S.I., Lascoux M. 2019. Parental legacy, demography, and admixture influenced the evolution of the two subgenomes of the tetraploid *Capsella bursa-pastoris* (Brassicaceae). PLoS Genet. 15(2):e1007949. 30768594 10.1371/journal.pgen.1007949 PMC6395008

[bib92] Kuzmanović N., Lakušić D., Frajman B., Alegro A., Schönswetter P. 2017. Phylogenetic relationships in Seslerieae (Poaceae) including resurrection of *Psilathera* and *Sesleriella*, two monotypic genera endemic to the Alps. Taxon. 66(6):1349–1370.

[bib93] Lavania U.C. 1991. Polyploid Breeding: meiosis in the diploid progenitor and its predictive value for fertility in the autotetraploid. Proc. Indian Natl. Sci. Acad. Part B Biol. Sci. 57:17–24.

[bib94] Lawson D.J., Van Dorp L., Falush D. 2018. A tutorial on how not to over-interpret STRUCTURE and ADMIXTURE bar plots. Nat. Commun. 9(1):3258. 30108219 10.1038/s41467-018-05257-7 PMC6092366

[bib95] Leal J.L., Milesi P., Hodková E., Zhou Q., James J., Eklund D.M., Pyhäjärvi T., Salojärvi J., Lascoux M. 2024. Complex polyploids: origins, genomic composition, and role of introgressed alleles. Syst. Biol. 73(2):392–418. 38613229 10.1093/sysbio/syae012 PMC11282369

[bib96] Leal J.L., Milesi P., Salojärvi J., Lascoux M. 2023. Phylogenetic analysis of allotetraploid species using polarized genomic sequences. Syst. Biol. 72(2):372–390. 36932679 10.1093/sysbio/syad009 PMC10275558

[bib97] Le Comber S.C., Ainouche M.L., Kovarik A., Leitch A.R. 2010. Making a functional diploid: from polysomic to disomic inheritance. New Phytol. 186(1):113–122. 20028473 10.1111/j.1469-8137.2009.03117.x

[bib98] Leigh J.W., Bryant D. 2015. POPART: full-feature software for haplotype network construction. Methods Ecol. Evol. 6(9):1110–1116.

[bib99] Levin D.A. 2002. The role of chromosomal change in plant evolution. New York (NY): Oxford University Press.

[bib100] Lewis P.O. 2001. A likelihood approach to estimating phylogeny from discrete morphological character data. Syst. Biol. 50(6):913–925. 12116640 10.1080/106351501753462876

[bib101] Li A., Geng S., Zhang L., Liu D., Mao L. 2015. Making the bread: insights from newly synthesized allohexaploid wheat. Mol. Plant. 8(6):847–859. 25747845 10.1016/j.molp.2015.02.016

[bib102] Li H., Durbin R. 2009. Fast and accurate short read alignment with Burrows–Wheeler transform. Bioinformatics. 25(14):1754–1760. 19451168 10.1093/bioinformatics/btp324 PMC2705234

[bib103] Lipnerova I., Bures P., Horova L., Smarda P. 2013. Evolution of genome size in *Carex* (Cyperaceae) in relation to chromosome number and genomic base composition. Ann. Bot. 111(1):79–94. 23175591 10.1093/aob/mcs239 PMC3523652

[bib104] Liu L., Xi Z., Wu S., Davis C.C., Edwards S.V. 2015. Estimating phylogenetic trees from genome-scale data. Ann. N.Y. Acad. Sci. 1360(1):36–53. 25873435 10.1111/nyas.12747

[bib105] Lloyd A., Bomblies K. 2016. Meiosis in autopolyploid and allopolyploid *Arabidopsis*. Curr. Opin. Plant Biol. 30:116–122. 26950252 10.1016/j.pbi.2016.02.004

[bib106] Loidl J., Ehrendorfer F., Schweizer D. 1990. EM analysis of meiotic chromosome pairing in a pentaploid *Achillea* hybrid. Heredity. 65(1):11–20.

[bib107] Luceño M., Guerra M. 1996. Numerical variations in species exhibiting holocentric chromosomes: a nomenclatural proposal. Caryologia. 49:301–309.

[bib108] Ma J.-X., Li Y.-N., Vogl C., Ehrendorfer F., Guo Y.-P. 2010. Allopolyploid speciation and ongoing backcrossing between diploid progenitor and tetraploid progeny lineages in the *Achillea millefolium* species complex: analyses of single-copy nuclear genes and genomic AFLP. BMC Evol. Biol. 10(1):100. 20388203 10.1186/1471-2148-10-100 PMC2873412

[bib109] Malheiros N., Gardé A. 1950. Fragmentation as a possible evolutionary process in the genus *Luzula* DC. Genética Ibérica. 2:257–262.

[bib110] Malinsky M., Matschiner M., Svardal H. 2021. Dsuite—fast *D*-statistics and related admixture evidence from VCF files. Mol. Ecol. Resour. 21(2):584–595. 33012121 10.1111/1755-0998.13265 PMC7116594

[bib111] Malinsky M., Svardal H., Tyers A.M., Miska E.A., Genner M.J., Turner G.F., Durbin R. 2018. Whole-genome sequences of Malawi cichlids reveal multiple radiations interconnected by gene flow. Nat. Ecol. Evol. 2(12):1940–1955. 30455444 10.1038/s41559-018-0717-x PMC6443041

[bib112] Mandel J.R., Barker M.S., Bayer R.J., Dikow R.B., Gao T., Jones K.E., Keeley S., Kilian N., Ma H., Siniscalchi C.M., Susanna A., Thapa R., Watson L., Funk V.A. 2017. The Compositae tree of life in the age of phylogenomics. J. Syt. Evol. 55(4):405–410.

[bib113] Marburger S., Monnahan P., Seear P.J., Martin S.H., Koch J., Paajanen P., Bohutínská M., Higgins J.D., Schmickl R., Yant L. 2019. Interspecific introgression mediates adaptation to whole genome duplication. Nat. Commun. 10(1):5218. 31740675 10.1038/s41467-019-13159-5 PMC6861236

[bib114] Marcussen T., Heier L., Brysting A.K., Oxelman B., Jakobsen K.S. 2015. From gene trees to a dated allopolyploid network: insights from the angiosperm genus *Viola* (Violaceae). Syst. Biol. 64(1):84–101. 25281848 10.1093/sysbio/syu071 PMC4265142

[bib115] Martinez-Perez E., Schvarzstein M., Barroso C., Lightfoot J., Dernburg A.F., Villeneuve A.M. 2008. Crossovers trigger a remodeling of meiotic chromosome axis composition that is linked to two-step loss of sister chromatid cohesion. Genes Dev. 22(20):2886–2901. 18923085 10.1101/gad.1694108 PMC2569886

[bib116] Mason A.S., Wendel J.F. 2020. Homoeologous exchanges, segmental allopolyploidy, and polyploid genome evolution. Front. Genet. 11:1014. 33005183 10.3389/fgene.2020.01014 PMC7485112

[bib117] McDonald D.B., Parchman T.L., Bower M.R., Hubert W.A., Rahel F.J. 2008. An introduced and a native vertebrate hybridize to form a genetic bridge to a second native species. Proc. Natl. Acad. Sci. USA. 105(31):10837–10842. 18658235 10.1073/pnas.0712002105 PMC2504823

[bib118] McKenna A., Hanna M., Banks E., Sivachenko A., Cibulskis K., Kernytsky A., Garimella K., Altshuler D., Gabriel S., Daly M., DePristo M.A. 2010. The genome analysis toolkit: a MapReduce framework for analyzing next-generation DNA sequencing data. Genome Res. 20(9):1297–1303. 20644199 10.1101/gr.107524.110 PMC2928508

[bib119] Meirmans P.G. 2015. Seven common mistakes in population genetics and how to avoid them. Mol. Ecol. 24(13):3223–3231. 25974103 10.1111/mec.13243

[bib120] Melters D.P., Paliulis L.V., Korf I.F., Chan S.W.L. 2012. Holocentric chromosomes: convergent evolution, meiotic adaptations, and genomic analysis. Chromosome Res. 20(5):579–593. 22766638 10.1007/s10577-012-9292-1

[bib121] Minh B.Q., Schmidt H.A., Chernomor O., Schrempf D., Woodhams M.D., Von Haeseler A., Lanfear R. 2020. IQ-TREE 2: new models and efficient methods for phylogenetic inference in the genomic era. Mol. Biol. Evol. 37(5):1530–1534. 32011700 10.1093/molbev/msaa015 PMC7182206

[bib122] Monnahan P., Kolář F., Baduel P., Sailer C., Koch J., Horvath R., Laenen B., Schmickl R., Paajanen P., Šrámková G., Bohutínská M., Arnold B., Weisman C.M., Marhold K., Slotte T., Bomblies K., Yant L. 2019. Pervasive population genomic consequences of genome duplication in *Arabidopsis arenosa*. Nat. Ecol. Evol. 3(3):457–468. 30804518 10.1038/s41559-019-0807-4

[bib123] Morrone O., Aagesen L., Scataglini M.A., Salariato D.L., Denham S.S., Chemisquy M.A., Sede S.M., Giussani L.M., Kellogg E.A., Zuloaga F.O. 2012. Phylogeny of the Paniceae (Poaceae: panicoideae): integrating plastid DNA sequences and morphology into a new classification. Cladistics. 28(4):333–356. 34836451 10.1111/j.1096-0031.2011.00384.x

[bib124] Müller K. 2005. SeqState: primer design and sequence statistics for phylogenetic DNA datasets. Appl. Bioinformatics. 4:65–69. 16000015 10.2165/00822942-200504010-00008

[bib125] Nei M. 1972. Genetic distance between populations. Am. Nat. 106(949):283–292.

[bib126] Nibau C., Gonzalo A., Evans A., Sweet‐Jones W., Phillips D., Lloyd A. 2022. Meiosis in allopolyploid *Arabidopsis suecica*. Plant J. 111(4):1110–1122. 35759495 10.1111/tpj.15879 PMC9545853

[bib127] Nieto Feliner G., Álvarez I., Fuertes-Aguilar J., Heuertz M., Marques I., Moharrek F., Piñeiro R., Riina R., Rosselló J.A., Soltis P.S., Villa-Machío I. 2017. Is homoploid hybrid speciation that rare? An empiricist’s view. Heredity. 118(6):513–516. 28295029 10.1038/hdy.2017.7 PMC5436029

[bib128] Nokkala S., Kuznetsova V.G., Maryanska-Nadachowska A., Nokkala C. 2004. Holocentric chromosomes in meiosis. I. Restriction of the number of chiasmata in bivalents. Chromosome Res. 12(7):733–739. 15505408 10.1023/B:CHRO.0000045797.74375.70

[bib129] Nordenskiöld H. 1951. Cyto-taxonomical studies in the genus *Luzula*: I. somatic chromosomes and chromosome numbers. Hereditas. 37(3):325–355.

[bib130] Nordenskiöld H. 1956. Cyto-taxonomical studies in the genus *Luzula* II. Hybridization experiments in the *campestris-multiflora* complex. Hereditas. 42(1–2):7–73.

[bib131] Nordenskiöld H. 1961. Tetrad analysis and the course of meiosis in three hybrids of *Luzula campestris*. Hereditas. 47(2):203–238.

[bib132] Nordenskiöld H. 1962. Studies of meiosis in *Luzula purpurea*. Hereditas. 48(3):503–519.

[bib133] Nylander J.A.A. 2004. MrAIC.Pl. program distributed by the author. Uppsala: Uppsala University.

[bib134] Owens G.L., Huang K., Todesco M., Rieseberg L.H. 2023. Re-evaluating homoploid reticulate evolution in *Helianthus* sunflowers. Mol. Biol. Evol. 40(2):msad013. 36648104 10.1093/molbev/msad013 PMC9907532

[bib135] Parisod C., Holderegger R., Brochmann C. 2010. Evolutionary consequences of autopolyploidy. New Phytol. 186(1):5–17. 20070540 10.1111/j.1469-8137.2009.03142.x

[bib136] Paun O., Forest F., Fay M.F., Chase M.W. 2009. Hybrid speciation in angiosperms: parental divergence drives ploidy. New Phytol. 182(2):507–518. 19220761 10.1111/j.1469-8137.2009.02767.x PMC2988484

[bib137] Peng Y., Yan H., Guo L., Deng C., Wang C., Wang Y., Kang L., Zhou P., Yu K., Dong X., Liu X., Sun Z., Peng Y., Zhao J., Deng D., Xu Y., Li Y., Jiang Q., Li Y., Wei L., Wang J., Ma J., Hao M., Li W., Kang H., Peng Z., Liu D., Jia J., Zheng Y., Ma T., Wei Y., Lu F., Ren C. 2022. Reference genome assemblies reveal the origin and evolution of allohexaploid oat. Nat. Genet. 54(8):1248–1258. 35851189 10.1038/s41588-022-01127-7 PMC9355876

[bib138] Perrie L.R., Shepherd L.D., De Lange P.J., Brownsey P.J. 2010. Parallel polyploid speciation: distinct sympatric gene-pools of recurrently derived allo-octoploid *Asplenium* ferns. Mol. Ecol. 19(14):2916–2932. 20579287 10.1111/j.1365-294X.2010.04705.x

[bib139] Pickrell J., Pritchard J. 2012. Inference of population splits and mixtures from genome-wide allele frequency data. PLoS Genet. 8(11): e1002967 23166502 10.1371/journal.pgen.1002967 PMC3499260

[bib140] Pritchard J.K., Stephens M., Donnelly P. 2000. Inference of population structure using multilocus genotype data. Genetics. 155(2):945–959. 10835412 10.1093/genetics/155.2.945 PMC1461096

[bib141] Ptáček J., Ekrt L., Hornych O., Urfus T. 2023. Interploidy gene flow via a “pentaploid bridge” and ploidy reduction in *Cystopteris fragilis* fern complex (Cystopteridaceae: Polypodiales). Plant Reprod. 36:321–331. 37532893 10.1007/s00497-023-00476-5

[bib142] Pungaršek Š., Dolenc Koce J., Bačič M., Barfuss M.H.J., Schönswetter P., Frajman B. 2023. Disentangling relationships among the Alpine species of *Luzula* sect. *Luzula* (Juncaceae) in the Eastern Alps. Plants. 12(4):973. 36840321 10.3390/plants12040973 PMC9960804

[bib143] R Core Team . 2020. R: a language and environment for statistical computing. Vienna: R Foundation for Statistical Computing.

[bib144] Rambaut A., Drummond A.J., Xie D., Baele G., Suchard M.A. 2018. Posterior summarization in Bayesian phylogenetics using Tracer 1.7. Syst. Biol. 67(5):901–904. 29718447 10.1093/sysbio/syy032 PMC6101584

[bib145] Ramsey J., Schemske D.W. 2002. Neopolyploidy in flowering plants. Annu. Rev. Ecol. Syst. 33(1):589–639.

[bib146] Rieseberg L.H., Van Fossen C., Desrochers A.M. 1995. Hybrid speciation accompanied by genomic reorganization in wild sunflowers. Nature. 375(6529):313–316.

[bib147] Ronquist F., Teslenko M., Van Der Mark P., Ayres D.L., Darling A., Höhna S., Larget B., Liu L., Suchard M.A., Huelsenbeck J.P. 2012. MrBayes 3.2: efficient Bayesian phylogenetic inference and model choice across a large model space. Syst. Biol. 61(3):539–542. 22357727 10.1093/sysbio/sys029 PMC3329765

[bib148] Rosser N., Seixas F., Queste L.M., Cama B., Mori-Pezo R., Kryvokhyzha D., Nelson M., Waite-Hudson R., Goringe M., Costa M., Elias M., Mendes Eleres De Figueiredo C., Freitas A.V.L., Joron M., Kozak K., Lamas G., Martins A.R.P., McMillan W.O., Ready J., Rueda-Muñoz N., Salazar C., Salazar P., Schulz S., Shirai L.T., Silva-Brandão K.L., Mallet J., Dasmahapatra K.K. 2024. Hybrid speciation driven by multilocus introgression of ecological traits. Nature. 628(8009):811–817. 38632397 10.1038/s41586-024-07263-w PMC11041799

[bib149] Sancho R., Inda L.A., Díaz‐Pérez A., Des Marais D.L., Gordon S., Vogel J.P., Lusinska J., Hasterok R., Contreras‐Moreira B., Catalán P. 2022. Tracking the ancestry of known and “ghost” homeologous subgenomes in model grass *Brachypodium* polyploids. Plant J. 109(6):1535–1558. 34951515 10.1111/tpj.15650

[bib150] Schönswetter P., Suda J., Popp M., Weiss-Schneeweiss H., Brochmann C. 2007. Circumpolar phylogeography of *Juncus biglumis* (Juncaceae) inferred from AFLP fingerprints, cpDNA sequences, nuclear DNA content and chromosome numbers. Mol. Phylogenet. Evol. 42(1):92–103. 16905337 10.1016/j.ympev.2006.06.016

[bib151] Schumer M., Rosenthal G.G., Andolfatto P. 2014. How common is homoploid hybrid speciation?. Evolution. 68(6):1553–1560. 24620775 10.1111/evo.12399

[bib152] Scott A.D., Van De Velde J.D., Novikova P.Y. 2023. Inference of polyploid origin and inheritance mode from population genomic data. In: Van De Peer Y., editor. Polyploidy. New York, NY: Springer US. p. 279–295. 10.1007/978-1-0716-2561-3_15 36720819

[bib153] Simmons M.P., Ochoterena H. 2000. Gaps as characters in sequence-based phylogenetic analyses. Syst. Biol. 49(2):369–381. 12118412

[bib154] Smith T.W., Kron P., Martin S.L. 2018. flowPloidy: an R package for genome size and ploidy assessment of flow cytometry data. Appl. Plant Sci. 6(7):e01164. 30131906 10.1002/aps3.1164 PMC6055564

[bib155] Soltis D.E., Albert V.A., Leebens‐Mack J., Bell C.D., Paterson A.H., Zheng C., Sankoff D., de Pamphilis C.W., Wall P.K., Soltis P.S. 2009. Polyploidy and angiosperm diversification. Am. J of Bot. 96(1):336–348. 21628192 10.3732/ajb.0800079

[bib156] Soltis P.S., Soltis D.E. 2009. The role of hybridization in plant speciation. Annu. Rev. Plant Biol. 60(1):561–588. 19575590 10.1146/annurev.arplant.043008.092039

[bib157] Som A. 2015. Causes, consequences and solutions of phylogenetic incongruence. Briefings Bioinf. 16(3):536–548. 10.1093/bib/bbu015 24872401

[bib158] Španiel S., Šlenker M., Melichárková A., Caboňová M., Šandalová M., Zeisek V., Marhold K., Zozomová-Lihová J. 2023. Phylogenetic challenges in a recently diversified and polyploid-rich *Alyssum* (Brassicaceae) lineage: low divergence, reticulation, and parallel polyploid speciation. Evolution. 77:1226–1244. 36820521 10.1093/evolut/qpad035

[bib159] Srivastava S., Lavania U.C., Sybenga J. 1992. Genetic variation in meiotic behaviour and fertility in tetraploid *Hyoscyamus muticus*: correlation with diploid meiosis. Heredity. 68(3):231–239.

[bib160] Stebbins G.L. 1947. Types of polyploids: their classification and significance. Adv. Genet. 1:403–429. 20259289 10.1016/s0065-2660(08)60490-3

[bib161] Stebbins G.L. 1956. Cytogenetics and evolution of the grass family. Am. J Bot. 43(10):890–905.

[bib162] Stebbins G.L. 1959. The role of hybridization in evolution. Proc. Am. Philos. Soc. 103:231–251.

[bib163] Stift M., Berenos C., Kuperus P., Van Tienderen P.H. 2008. Segregation models for disomic, tetrasomic and intermediate inheritance in tetraploids: a general procedure applied to *Rorippa* (Yellow Cress) microsatellite data. Genetics. 179(4):2113–2123. 18689891 10.1534/genetics.107.085027 PMC2516083

[bib164] Stift M., Kolář F., Meirmans P.G. 2019. Structure is more robust than other clustering methods in simulated mixed-ploidy populations. Heredity. 123(4):429–441. 31285566 10.1038/s41437-019-0247-6 PMC6781132

[bib165] Suda J., Trávníček P. 2006. Estimation of relative nuclear DNA content in dehydrated plant tissues by flow cytometry. Curr. Protoc. Cytom. 38:1–14. 10.1002/0471142956.cy0730s38 18770844

[bib166] Svardal H., Quah F.X., Malinsky M., Ngatunga B.P., Miska E.A., Salzburger W., Genner M.J., Turner G.F., Durbin R. 2020. Ancestral hybridization facilitated species diversification in the Lake Malawi cichlid fish adaptive radiation. Mol. Biol. Evol. 37(4):1100–1113. 31821500 10.1093/molbev/msz294 PMC7086168

[bib167] Szadkowski E., Eber F., Huteau V., Lodé M., Huneau C., Belcram H., Coriton O., Manzanares‐Dauleux M.J., Delourme R., King G.J., Chalhoub B., Jenczewski E., Chèvre A.‐M. 2010. The first meiosis of resynthesized *Brassica napus*, a genome blender. New Phytol. 186(1):102–112. 20149113 10.1111/j.1469-8137.2010.03182.x

[bib168] Tel-zur N., Abbo S., Myslabodski D., Mizrahi Y. 1999. Modified CTAB procedure for DNA isolation from epiphytic cacti of the genera *Hylocereus* and *Selenicereus* (Cactaceae). Plant Mol. Biol. Rep. 17(3):249–254.

[bib169] Tiley G.P., Crowl A.A., Manos P.S., Sessa E.B., Solís-Lemus C., Yoder A.D., Burleigh J.G. 2024. Benefits and limits of phasing alleles for network inference of allopolyploid complexes. Syst. Biol. 73(4):666–682. 38733563 10.1093/sysbio/syae024

[bib170] Uitdewilligen J.G.A.M.L., Wolters A.-M.A., D’hoop B.B., Borm T.J.A., Visser R.G.F., Van Eck H.J. 2013. A next-generation sequencing method for genotyping-by-sequencing of highly heterozygous autotetraploid potato. PLoS One. 8(5):e62355. 23667470 10.1371/journal.pone.0062355 PMC3648547

[bib171] Vanzela A.L.L., Luceño M., Guerra M. 2000. Karyotype evolution and cytotaxonomy in Brazilian species of *Rhynchospora* Vahl (Cyperaceae). Bot. J. Linn. Soc. 134(4):557–566.

[bib172] Wagner N.D., He L., Hörandl E. 2020. Phylogenomic relationships and evolution of polyploid *Salix* species revealed by RAD sequencing data. Front. Plant Sci. 11:1077. 32765560 10.3389/fpls.2020.01077 PMC7379873

[bib173] Wang N., Kelly L.J., McAllister H.A., Zohren J., Buggs R.J.A. 2021. Resolving phylogeny and polyploid parentage using genus-wide genome-wide sequence data from birch trees. Mol. Phylogenet. Evol. 160:107126. 33647400 10.1016/j.ympev.2021.107126

[bib174] Wei N., Tennessen J.A., Liston A., Ashman T. 2017. Present-day sympatry belies the evolutionary origin of a high-order polyploid. New Phytol. 216(1):279–290. 28771729 10.1111/nph.14711 PMC5637924

[bib175] Weiß C.L., Pais M., Cano L.M., Kamoun S., Burbano H.A. 2018. nQuire: a statistical framework for ploidy estimation using next generation sequencing. BMC Bioinf. 19:122. 10.1186/s12859-018-2128-z PMC5885312 29618319

[bib176] Wood T.E., Takebayashi N., Barker M.S., Mayrose I., Greenspoon P.B., Rieseberg L.H. 2009. The frequency of polyploid speciation in vascular plants. Proc. Natl. Acad. Sci. USA. 106(33):13875–13879. 19667210 10.1073/pnas.0811575106 PMC2728988

[bib177] Wu S., Wang Y., Wang Z., Shrestha N., Liu J. 2022. Species divergence with gene flow and hybrid speciation on the Qinghai–Tibet Plateau. New Phytol. 234(2):392–404. 35020198 10.1111/nph.17956

[bib178] Wu Y., Lin F., Zhou Y., Wang J., Sun S., Wang B., Zhang Z., Li G., Lin X., Wang X., Sun Y., Dong Q., Xu C., Gong L., Wendel J.F., Zhang Z., Liu B. 2021. Genomic mosaicism due to homoeologous exchange generates extensive phenotypic diversity in nascent allopolyploids. Natl. Sci. Rev. 8(5):nwaa277. 34691642 10.1093/nsr/nwaa277 PMC8288387

[bib179] Xu C., Bai Y., Lin X., Zhao N., Hu L., Gong Z., Wendel J.F., Liu B. 2014. Genome-wide disruption of gene expression in allopolyploids but not hybrids of rice subspecies. Mol. Biol. Evol. 31(5):1066–1076. 24577842 10.1093/molbev/msu085 PMC3995341

[bib180] Yakimowski S.B., Rieseberg L.H. 2014. The role of homoploid hybridization in evolution: a century of studies synthesizing genetics and ecology. Am. J. Bot. 101(8):1247–1258. 25156978 10.3732/ajb.1400201

[bib181] Yang L., Harris A.J., Wen F., Li Z., Feng C., Kong H., Kang M. 2023. Phylogenomic analyses reveal an allopolyploid origin of Core Didymocarpinae (Gesneriaceae) followed by rapid radiation. Syst. Biol. 72(5):1064–1083. 37158589 10.1093/sysbio/syad029 PMC10627561

[bib182] Yano O., Hoshino T. 2005. Molecular phylogeny and chromosomal evolution of Japanese *Schoenoplectus* (Cyperaceae), based on ITS and ETS 1f Sequences. Acta Phytotax. Geobot. 56:183–195.

[bib183] Yant L., Hollister J.D., Wright K.M., Arnold B.J., Higgins J.D., Franklin F.C.H., Bomblies K. 2013. Meiotic adaptation to genome duplication in *Arabidopsis arenosa*. Curr. Biol. 23(21):2151–2156. 24139735 10.1016/j.cub.2013.08.059 PMC3859316

[bib184] Záveská Drábková L., Vlček Č. 2009. DNA variation within Juncaceae: comparison of impact of organelle regions on phylogeny. Plant Syst. Evol. 278(3–4):169–186.

[bib185] Záveská Drábková L., Vlček Č. 2010. Molecular phylogeny of the genus *Luzula* DC. (Juncaceae, Monocotyledones) based on plastome and nuclear ribosomal regions: a case of incongruence, incomplete lineage sorting and hybridisation. Mol. Phylogenet. Evol. 57(2):536–551. 20696260 10.1016/j.ympev.2010.07.022

[bib186] Zedek F., Bureš P. 2018. Holocentric chromosomes: from tolerance to fragmentation to colonization of the land. Ann. Bot. 121(1):9–16. 29069342 10.1093/aob/mcx118 PMC5786251

[bib187] Zhang C., Rabiee M., Sayyari E., Mirarab S. 2018. ASTRAL-III: polynomial time species tree reconstruction from partially resolved gene trees. BMC Bioinf. 19(S6):153. 10.1186/s12859-018-2129-y PMC5998893 29745866

[bib188] Zhang H., Bian Y., Gou X., Zhu B., Xu C., Qi B., Li N., Rustgi S., Zhou H., Han F., Jiang J., Von Wettstein D., Liu B. 2013. Persistent whole-chromosome aneuploidy is generally associated with nascent allohexaploid wheat. Proc. Natl. Acad. Sci. USA. 110(9):3447–3452. 23401544 10.1073/pnas.1300153110 PMC3587266

[bib189] Zohren J., Wang N., Kardailsky I., Borrell J.S., Joecker A., Nichols R.A., Buggs R.J.A. 2016. Unidirectional diploid–tetraploid introgression among British birch trees with shifting ranges shown by restriction site-associated markers. Mol. Ecol. 25(11):2413–2426. 27065091 10.1111/mec.13644 PMC4999052

