# Supplementary material for: Hybridization and Polyploidy Shaped the Evolutionary History of a Complex of Cryptic Species in European Woodrushes (Luzula sect. Luzula)
Source: Syst Biol. 2025 Sep 25;75(3):493–516. doi: 10.1093/sysbio/syaf065 (PMC13048012; doi:10.1093/sysbio/syaf065)
Supplement: syaf065_Supplemental_Files [file syaf065_supplemental_files.zip › Supplementary_Figures.pdf]

Supplementary Figures

Hybridization and Polyploidy shaped the Evolutionary History of a Complex of Cryptic Species in European Woodrushes (*Luzula* sect. *Luzula*)

Valentin Heimer[1,2*], Pau Carnicero[1,3], Carolina Carrizo García[4,5], Andreas Hilpold[2], Jasna Dolenc Koce[6], J. Luis Leal[7], Mingai Li[4], Claudio Varotto[4], Peter Schönswetter[1], Božo Frajman[1]

[1] *Department of Botany, University of Innsbruck, Sternwartestraße 15, 6020 Innsbruck, Austria*

[2] *Institute for Alpine Environment, Eurac Research, Drususallee 1/Viale Druso 1, 39100, Bozen/Bolzano, Italy*

[3] *Department of Animal Biology, Plant Biology and Ecology, Autonomous University of Barcelona, 08193 Bellaterra, Spain.*

[4] *Centro Ricerca e Innovazione, Fondazione Edmund Mach, Via Mach 1, 38098 San Michele all'Adige, Italy*

[5] *Instituto Multidisciplinario de Biología Vegetal (CONICET-UNC), Av. Vélez Sarsfield 1611, 5000 Córdoba, Argentina*

[6] *Department of Biology, Biotechnical Faculty, University of Ljubljana, Jamnikarjeva 101, 1000, Ljubljana, Slovenia*

[7] *Department of Zoology, Stockholm University, Svante Arrheniusväg 18 B, 106 91 Stockholm, Sweden*

[*] Corresponding author

E-mail address: valentin.heimer@uibk.ac.at

The following Supplementary Figures are available for this Article:

Supplementary Figure S1. Distribution of populations analyzed in this study for each species.

Supplementary Figure S2. Metaphase chromosomes of alpine tetraploids included in this study.

Supplementary Figure S3. Best-scoring maximum likelihood tree of diploid species of *Luzula* sect. *Luzula* and outgroup inferred in IQ-TREE 2 based on 27,664 SNPs derived from RADseq.

Supplementary Figure S4. Evanno plot for the selection of the best fitting K for STRUCTURE run on diploids only.

Supplementary Figure S5. STRUCTURE results for diploid *Luzula* sect. *Luzula* species for K = 1 to 12.

Supplementary Figure S6. Genotype frequencies relative to alternative allele frequency within each population of alpine tetraploids (*Luzula alpina* and tetraploid *L. multiflora*) and *L. divulgata*

Supplementary Figure S7. Smoothed distributions of relative allelic depth inferred from BAM files with nQuire for all individuals of *Luzula alpina*, tetraploid *L. multiflora* and *L. divulgata*.

Supplementary Figure S8. Evanno plot for the selection of the best fitting K for STRUCTURE run on alpine tetraploids only.

Supplementary Figure S9. STRUCTURE results for alpine tetraploids (*Luzula alpina* and tetraploid *L. multiflora*) for K = 1 to 10.

Supplementary Figure S10. STRUCTURE results for a subset of 25 individuals per species of diploid and polyploid species of *Luzula* sect. *Luzula* for K = 1 to 12.

Supplementary Figure S11. STRUCTURE results for diploid and polyploid species of *Luzula* sect. *Luzula* for K = 1 to 12.

Supplementary Figure S12. Best-scoring maximum likelihood tree of diploid and polyploid species of *Luzula* sect. *Luzula* and outgroup inferred in IQ-TREE 2 based on 9,112 SNPs derived from RADseq.

Supplementary Figure S13. Evanno plot for the selection of the best fitting K for STRUCTURE run a subset of 25 individuals per species of diploids and polyploids.

Supplementary Figure S14. Evanno plot for the selection of the best fitting K for STRUCTURE run on diploid and polyploid samples.

Supplementary Figure S15. Change in Log Likelihood for different numbers of migration edges (*m*) in the TreeMix analysis.

Supplementary Figure S16. Results of iterative genomic polarization of tetraploid *Luzula alpina*, *L. divulgata* and *L. multiflora* (4*x*).

Supplementary Figure S17. Bayesian consensus phylogram inferred from concatenated plastid sequences of diploid and polyploid species of *Luzula* sect. *Luzula* and outgroup.

Supplementary Figure S18. Phylogeographic patterns of plastid haplotypes within *Luzula* sect. *Luzula*.

Supplementary Figure S19. Plastid DNA haplotypes for the ten taxa of *Luzula* sect. *Luzula*.

Supplementary Figure S20. Distribution of relative genome size (RGS) in a dataset of 3462 samples of European *Luzula* sect. *Luzula* species.

Supplementary Figure S21. Genetic structure within alpine tetraploids and plastid haplotypes.

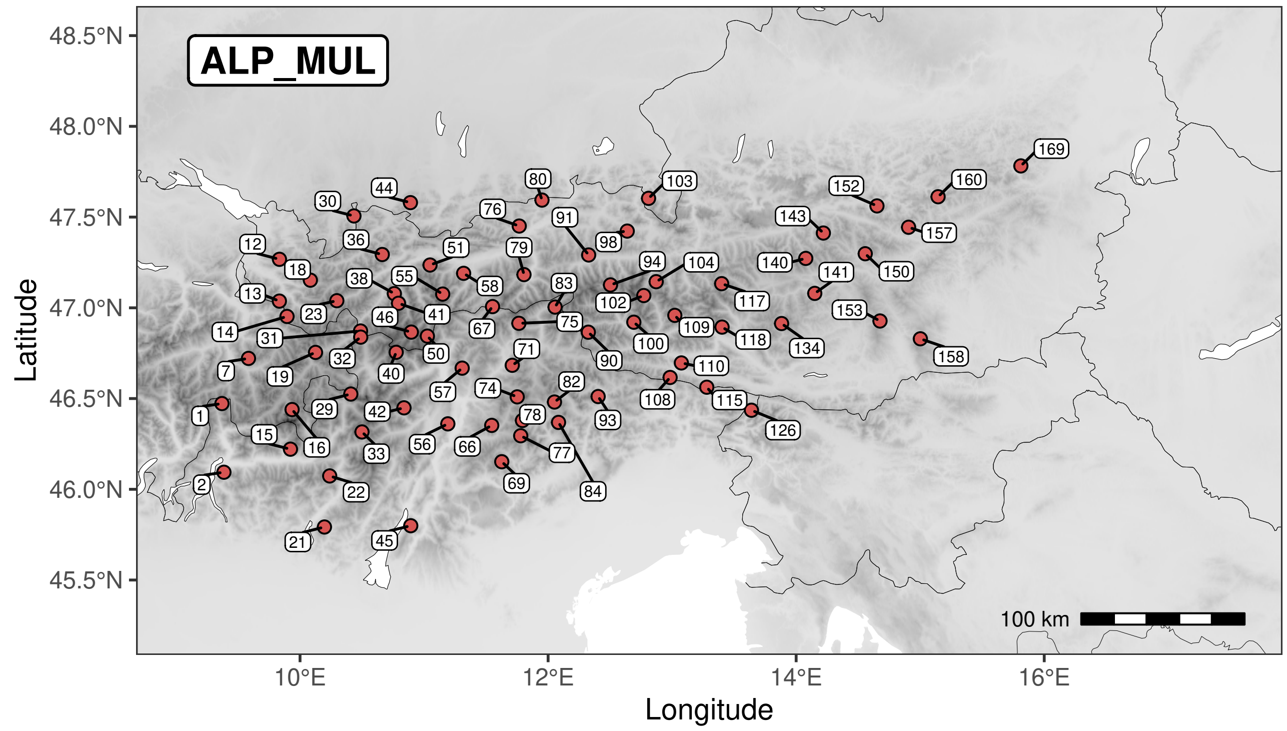
ALP_MUL
48.5°N
48.0°N
47.5°N
47.0°N
46.5°N
46.0°N
45.5°N
Latitude
10°E
12°E
14°E
16°E
Longitude
100 km
169
103
80
152
160
44
30
76
91
143
157
36
98
12
51
79
104
140
94
150
18
38
55
58
83
141
117
13
23
102
153
46
41
67
75
14
31
109
100
118
134
71
32
50
158
7
19
40
90
110
57
74
82
108
115
1
29
42
93
78
126
15
16
56
66
33
77
2
69
22
84
21
45

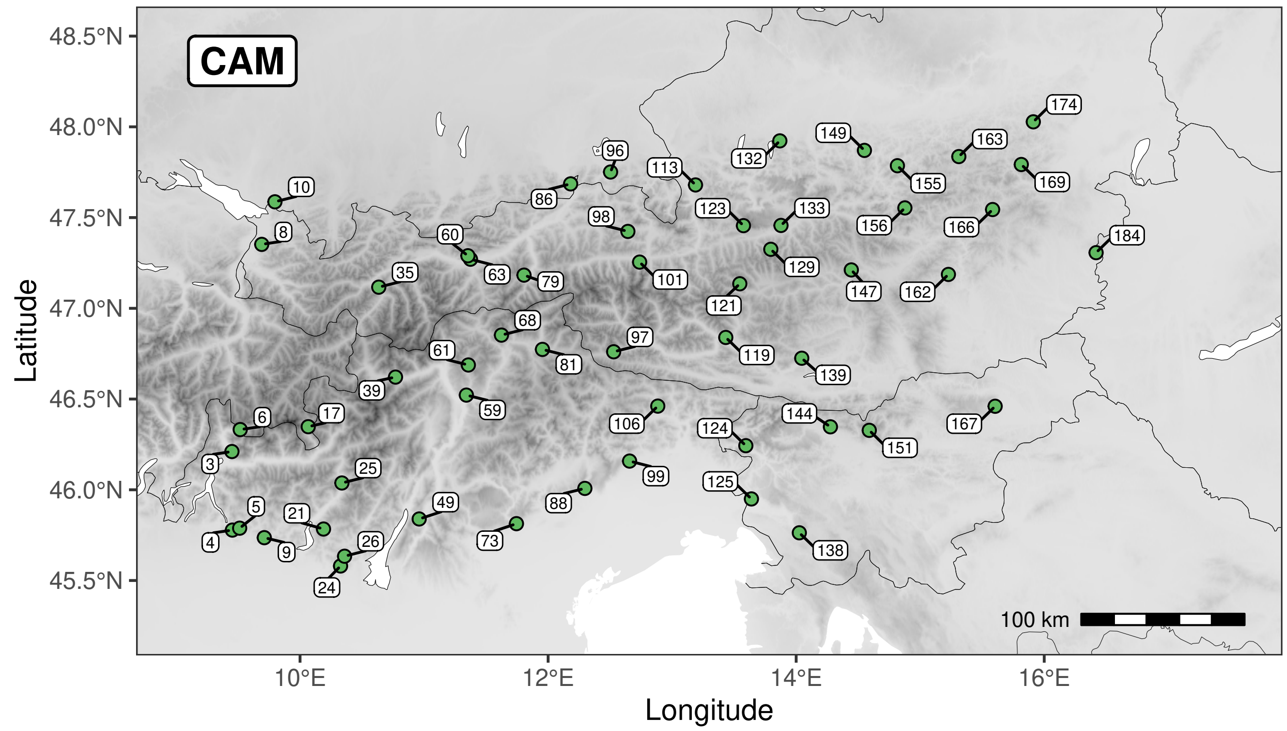
CAM
48.5°N
48.0°N
47.5°N
47.0°N
46.5°N
46.0°N
45.5°N
Latitude
10°E
12°E
14°E
16°E
Longitude
100 km
174
96
163
132
149
113
10
86
155
169
123
133
98
8
60
156
166
184
63
79
129
101
35
147
162
121
68
97
61
119
81
139
39
59
6
17
144
106
167
124
151
3
25
99
125
5
21
49
88
4
9
26
73
138
24

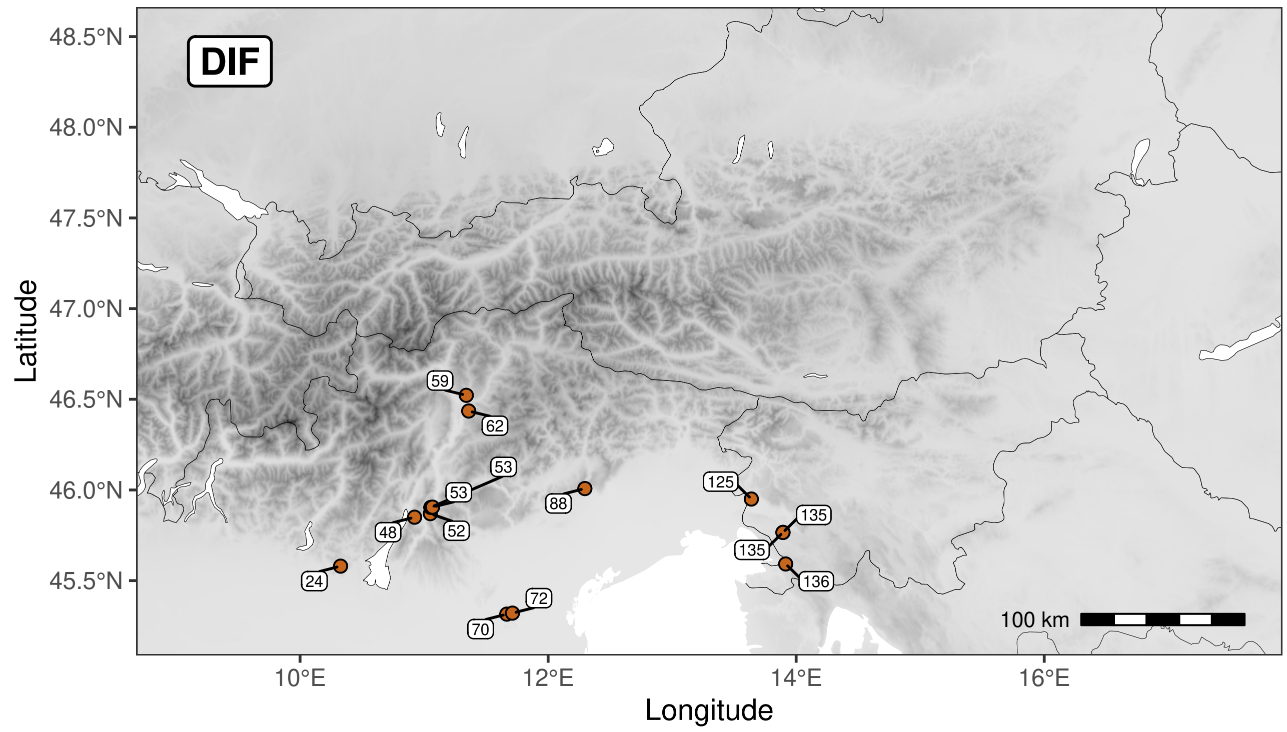
DIF
48.5°N
48.0°N
47.5°N
47.0°N
46.5°N
46.0°N
45.5°N
Latitude
10°E
12°E
14°E
16°E
Longitude
100 km
59
62
53
53
125
88
135
48
52
135
24
136
72
70

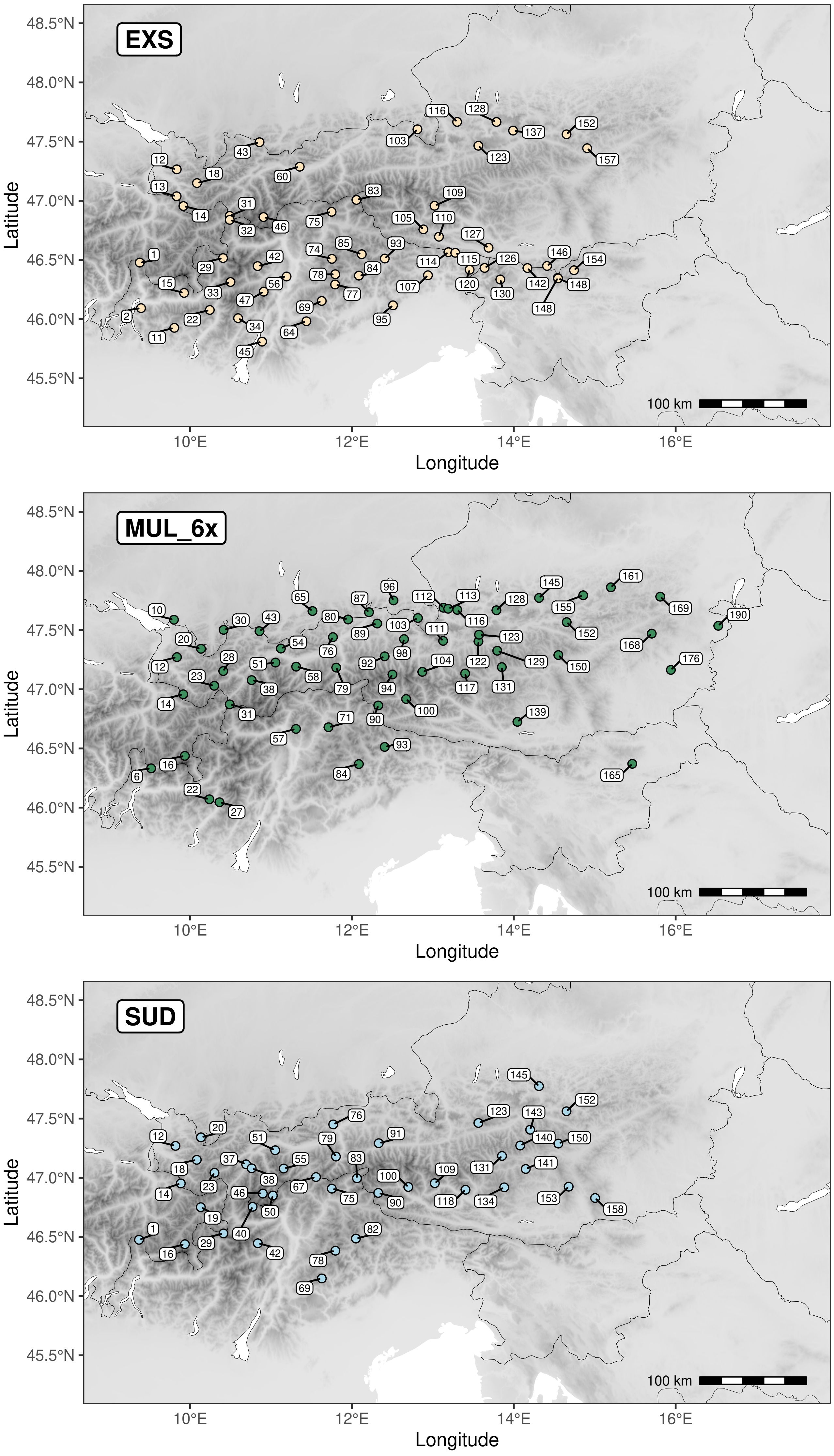
EXS
MUL_6x
SUD
48.5°N
48.0°N
47.5°N
47.0°N
46.5°N
46.0°N
45.5°N
10°E
12°E
14°E
16°E
Latitude
Longitude
100 km

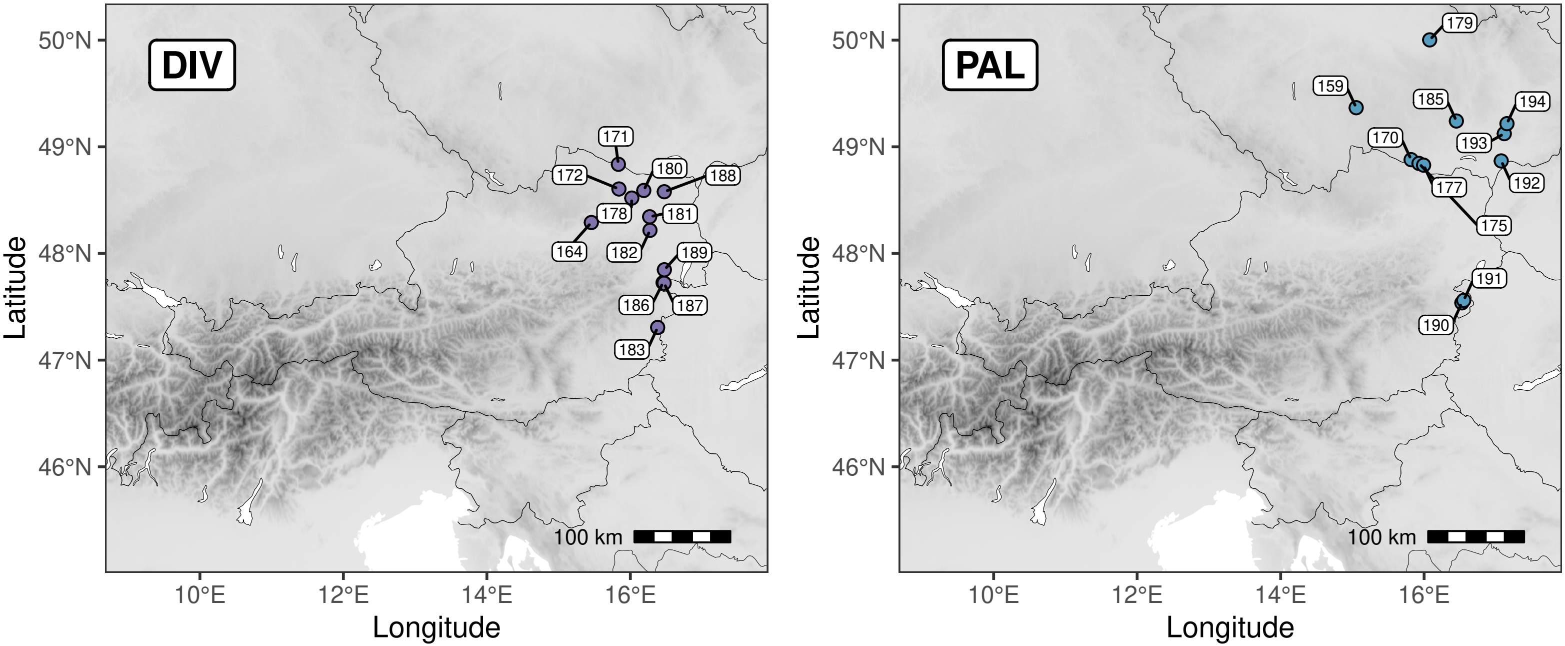

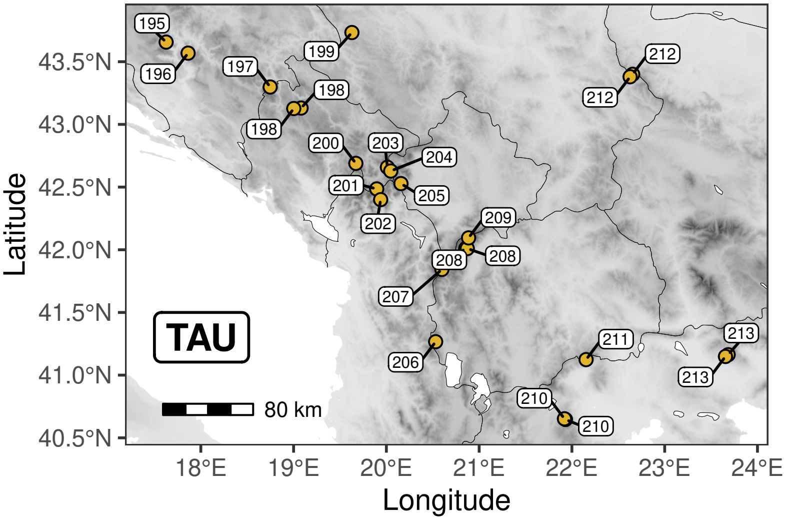

Supplementary Figure S1. Distribution of populations analyzed in this study for each species. Labels are population identifiers. *Luzula alpina* and tetraploid *L. multiflora* are combined in a single map as they were sampled collectively as 'alpine tetraploids' and often occur in mixed populations.

a)

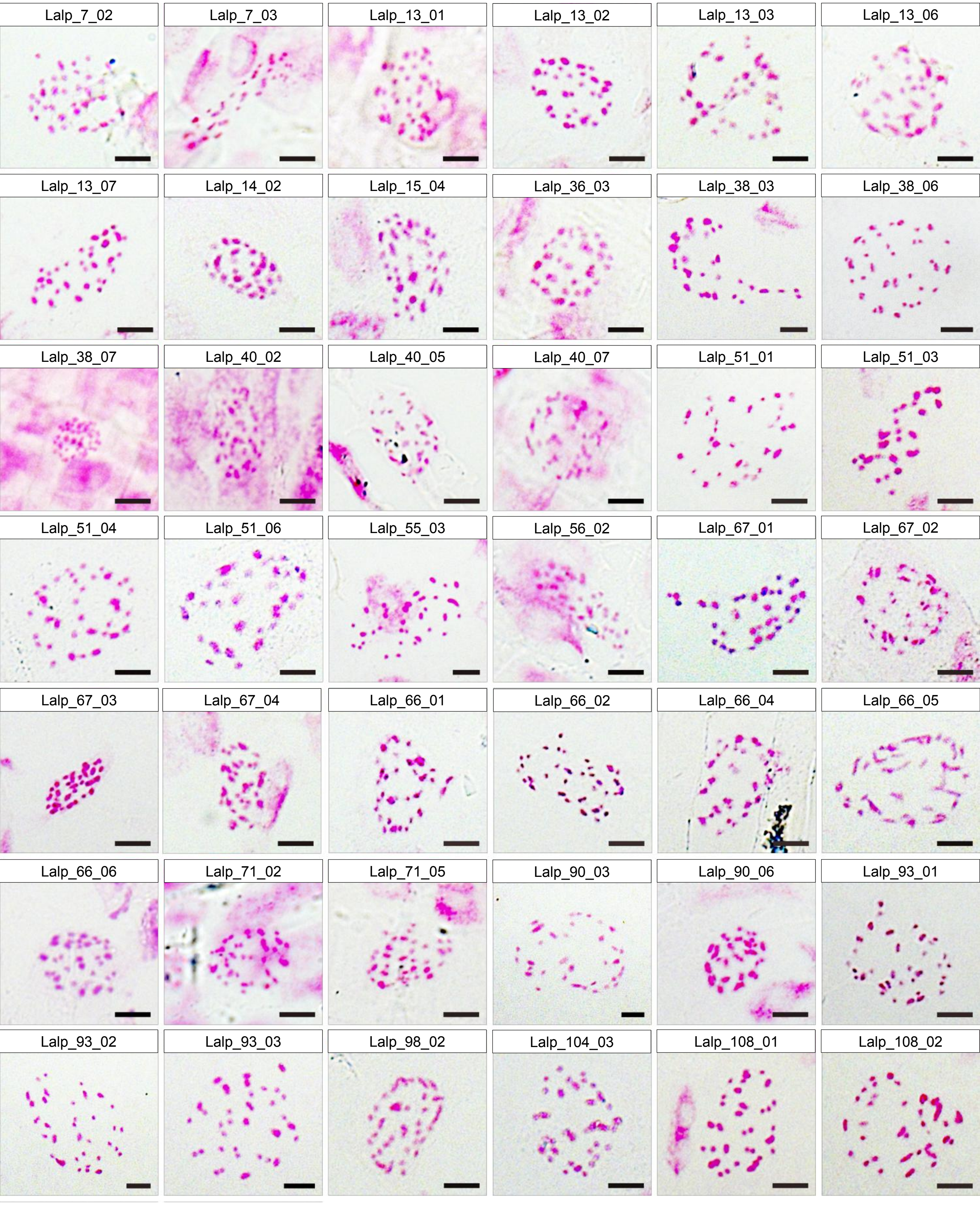
Lalp_7_02
Lalp_7_03
Lalp_13_01
Lalp_13_02
Lalp_13_03
Lalp_13_06
Lalp_13_07
Lalp_14_02
Lalp_15_04
Lalp_36_03
Lalp_38_03
Lalp_38_06
Lalp_38_07
Lalp_40_02
Lalp_40_05
Lalp_40_07
Lalp_51_01
Lalp_51_03
Lalp_51_04
Lalp_51_06
Lalp_55_03
Lalp_56_02
Lalp_67_01
Lalp_67_02
Lalp_67_03
Lalp_67_04
Lalp_66_01
Lalp_66_02
Lalp_66_04
Lalp_66_05
Lalp_66_06
Lalp_71_02
Lalp_71_05
Lalp_90_03
Lalp_90_06
Lalp_93_01
Lalp_93_02
Lalp_93_03
Lalp_98_02
Lalp_104_03
Lalp_108_01
Lalp_108_02

b)

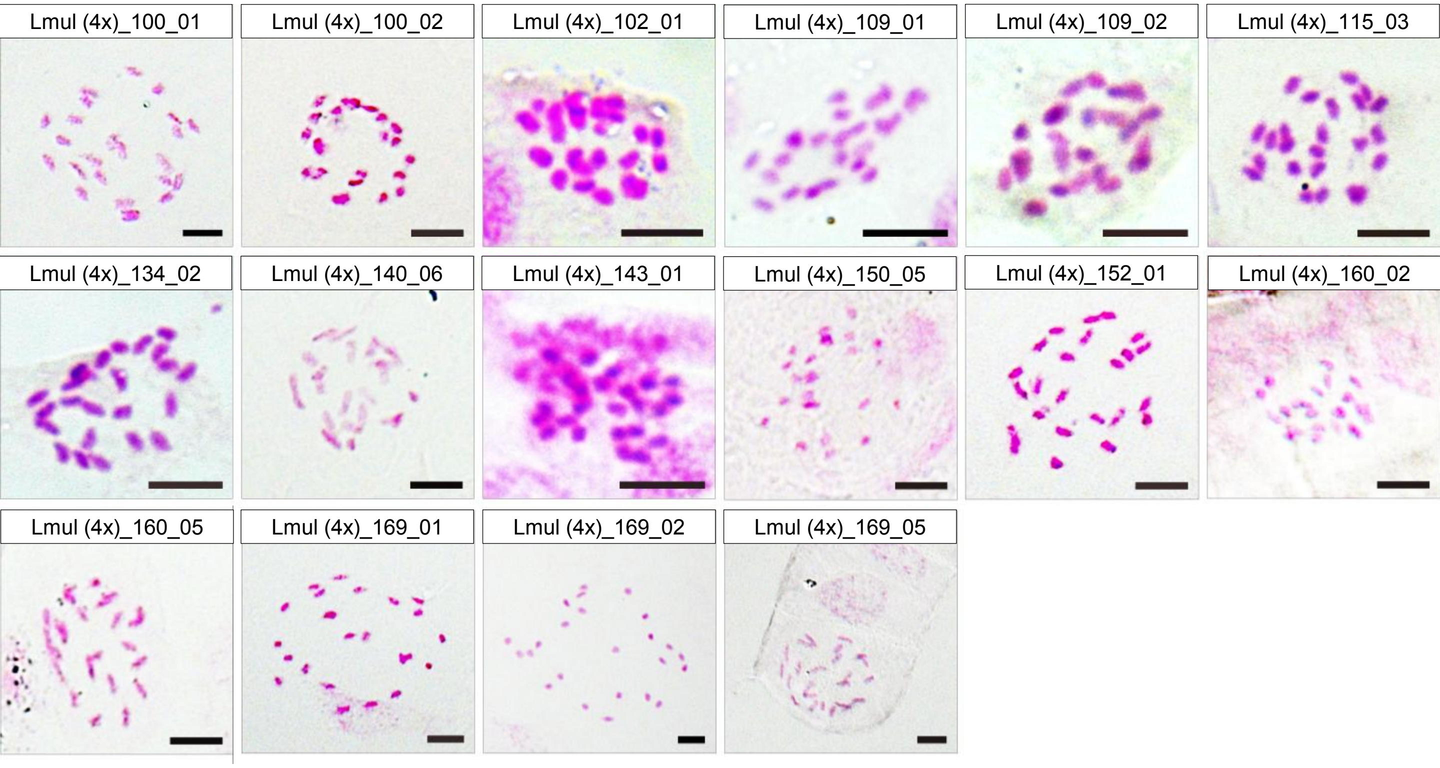

Supplementary Figure S2. Metaphase chromosomes of alpine tetraploids included in this study, either newly produced or from Pungaršek et al. (2023), corresponding to the 12AL+24BL cytotype of *Luzula alpina* (a) and the 24AL cytotype of tetraploid *L. multiflora* (b). Scale bars represent 5 µm.

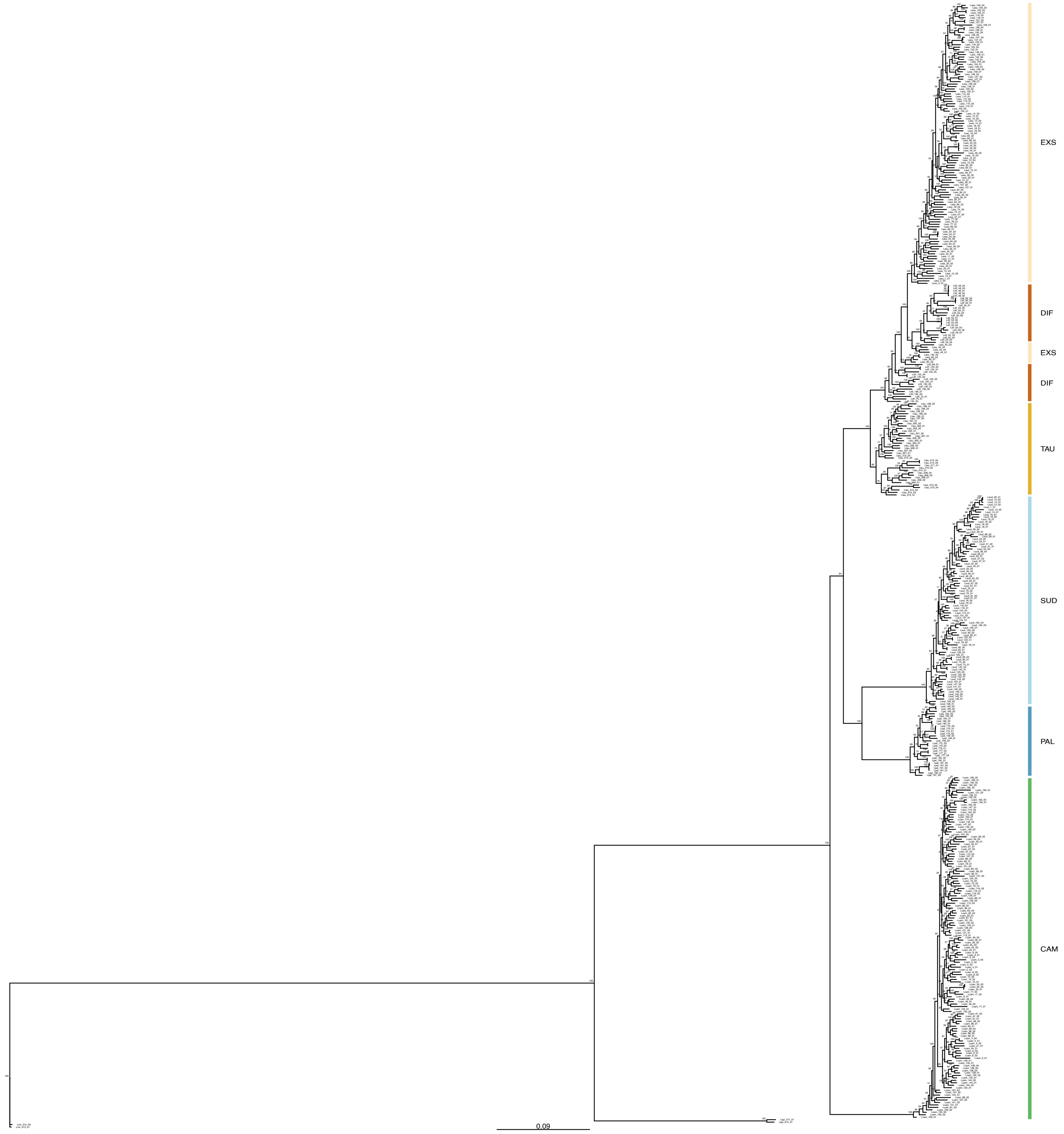

Supplementary Figure S3. Best-scoring maximum likelihood tree of diploid species of *Luzula* sect. *Luzula* and outgroup inferred in IQ-TREE 2 based on 27,664 SNPs derived from RADseq. Major clades corresponding to species are indicated on the right. Numbers above branches show bootstrap support values.

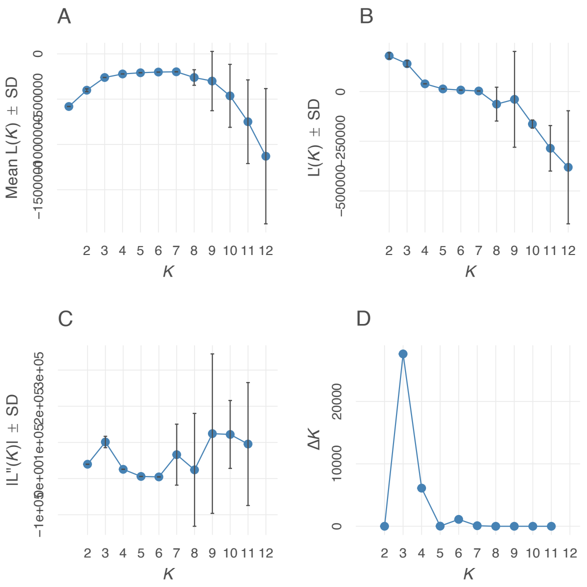

Supplementary Figure S4. Selection of the best fitting K for STRUCTURE run on diploids only. Change in likelihood (a), as well as first (b) and second (c) derivative and ΔK following Evanno for each K between 1 and 12.

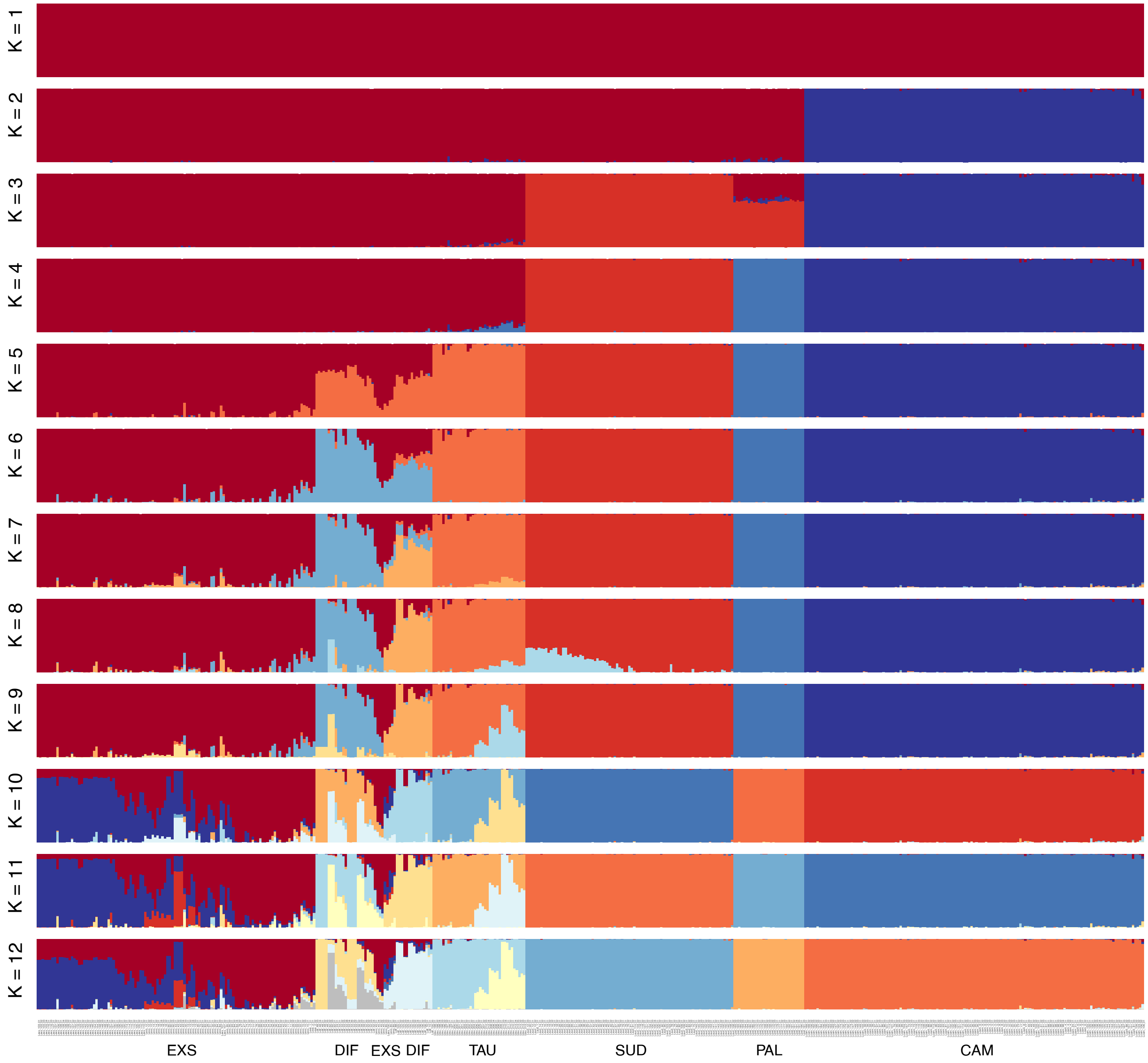

Supplementary Figure S5. STRUCTURE results for diploid *Luzula* sect. *Luzula* species for K = 1 to 12. Species are indicated below the plot and their order corresponds to that of clades in the ML tree.

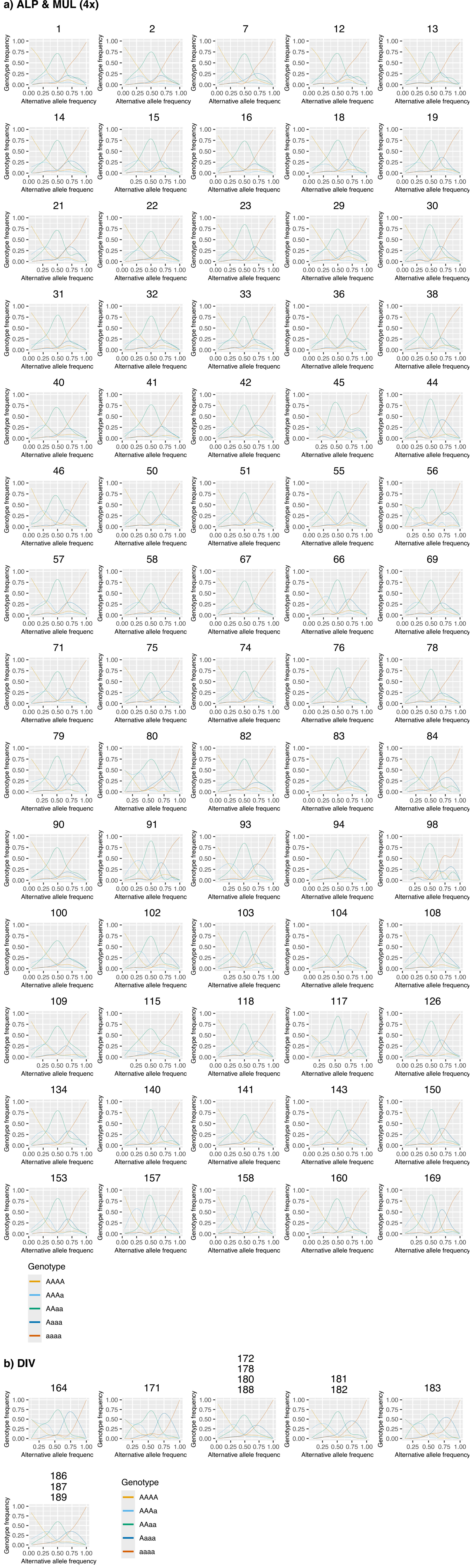

Supplementary Figure S6. Genotype frequencies (y-axis) relative to alternative allele frequency (x-axis) within each population of alpine tetraploids (*Luzula alpina* and tetraploid *L. multiflora*) (a) and *L. divulgata* (b). Genotype frequencies of alpine tetraploids show an excess of intermediate frequency genotypes (green), consistent with expectations of Hardy Weinberg equilibrium under disomic inheritance as found in allotetraploids. Patterns of genotype frequencies in *L. divulgata* are less conclusive but, at least in most cases, also indicate disomic inheritance.

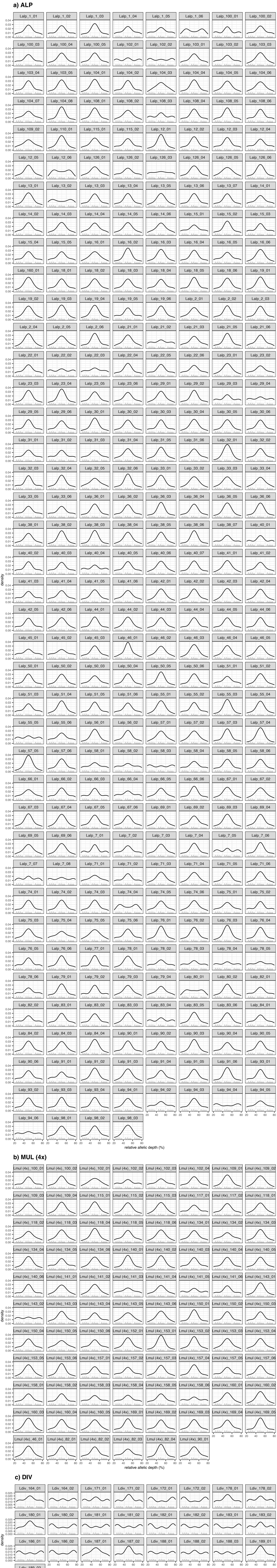

Supplementary Figure S7. Smoothed distributions of relative allelic depth inferred from BAM files with nQuire for all individuals of *Luzula alpina* (a), tetraploid *L. multiflora* (b) and *L. divulgata* (c). Most samples indicate an excess of intermediate allelic depth indicative of disomic inheritance and thus allopolyploid with the exception of six *L. alpina* individuals (Lalp_1_06, Lalp_12_06, Lalp_13_02, Lalp_74_04, Lalp_80_01, Lalp_94_04) that showed a pattern more fitting to tetrasomic inheritance. Similar to population-wide genotype frequencies (Supplemenatry Fig. S6), *Luzula divulgata* showed a mixed pattern of allelic depth distributions, with some individuals matching expectations of di- and others of tetrasomic inheritance.

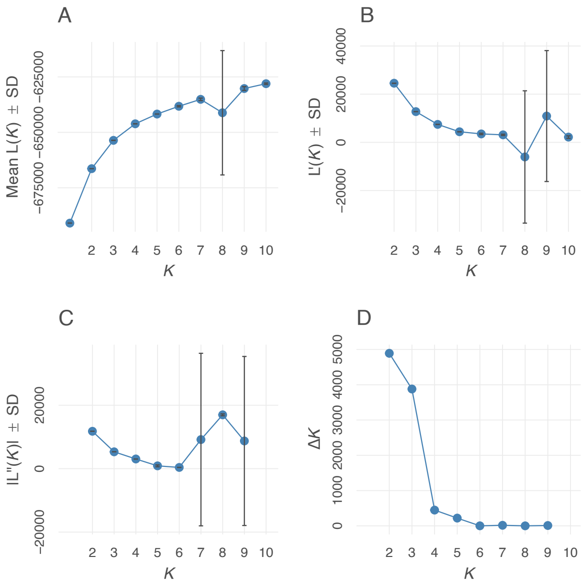

Supplementary Figure S8. Selection of the best fitting K for STRUCTURE run on alpine tetraploids only. Change in likelihood (a), as well as first (b) and second (c) derivative and ΔK following Evanno for each K between 1 and 10.

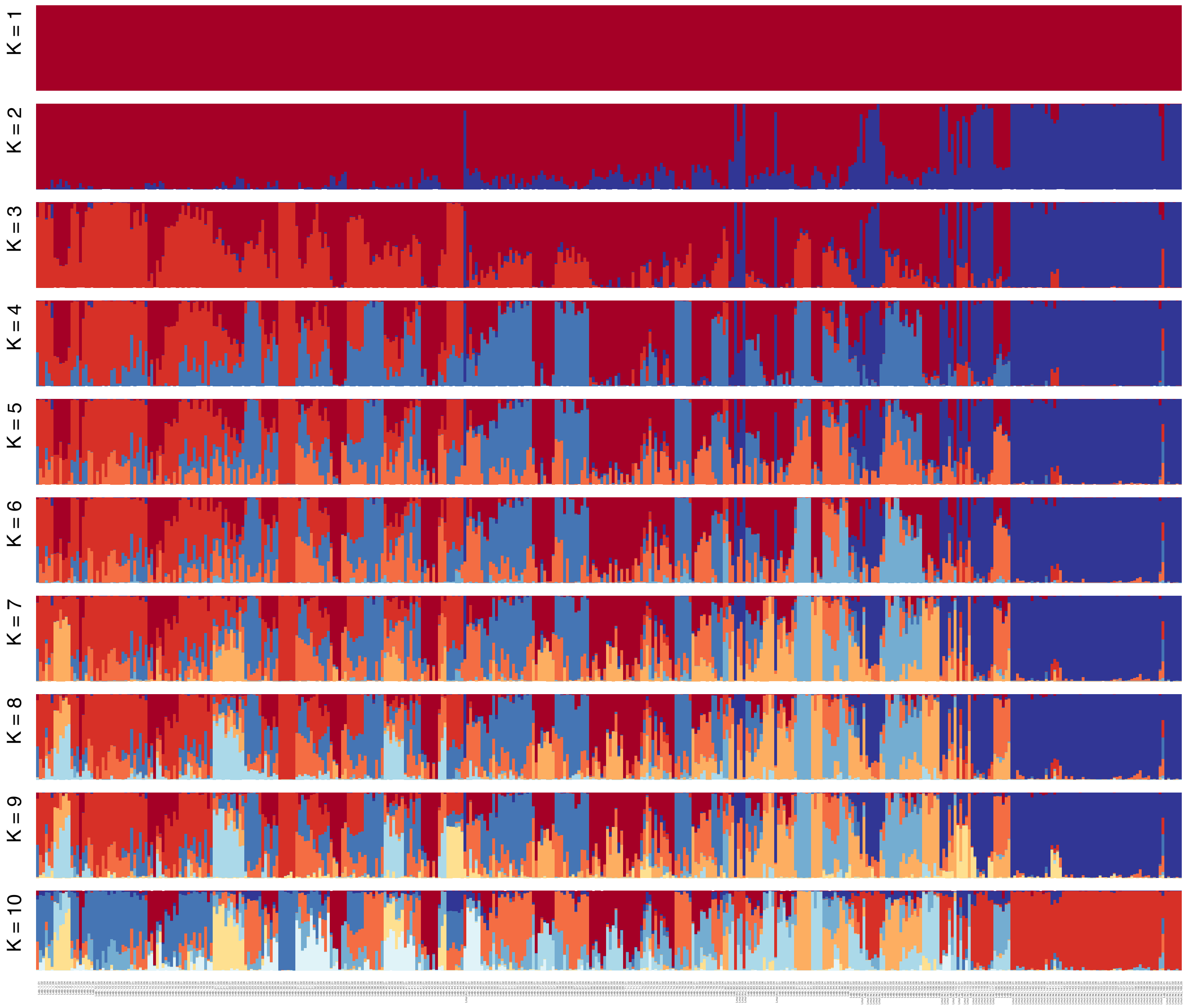

Supplementary Figure S9. STRUCTURE results for alpine tetraploids (*Luzula alpina* and tetraploid *L. multiflora*) for K = 1 to 10. Individuals are presented in the same order as in Fig. 3d.

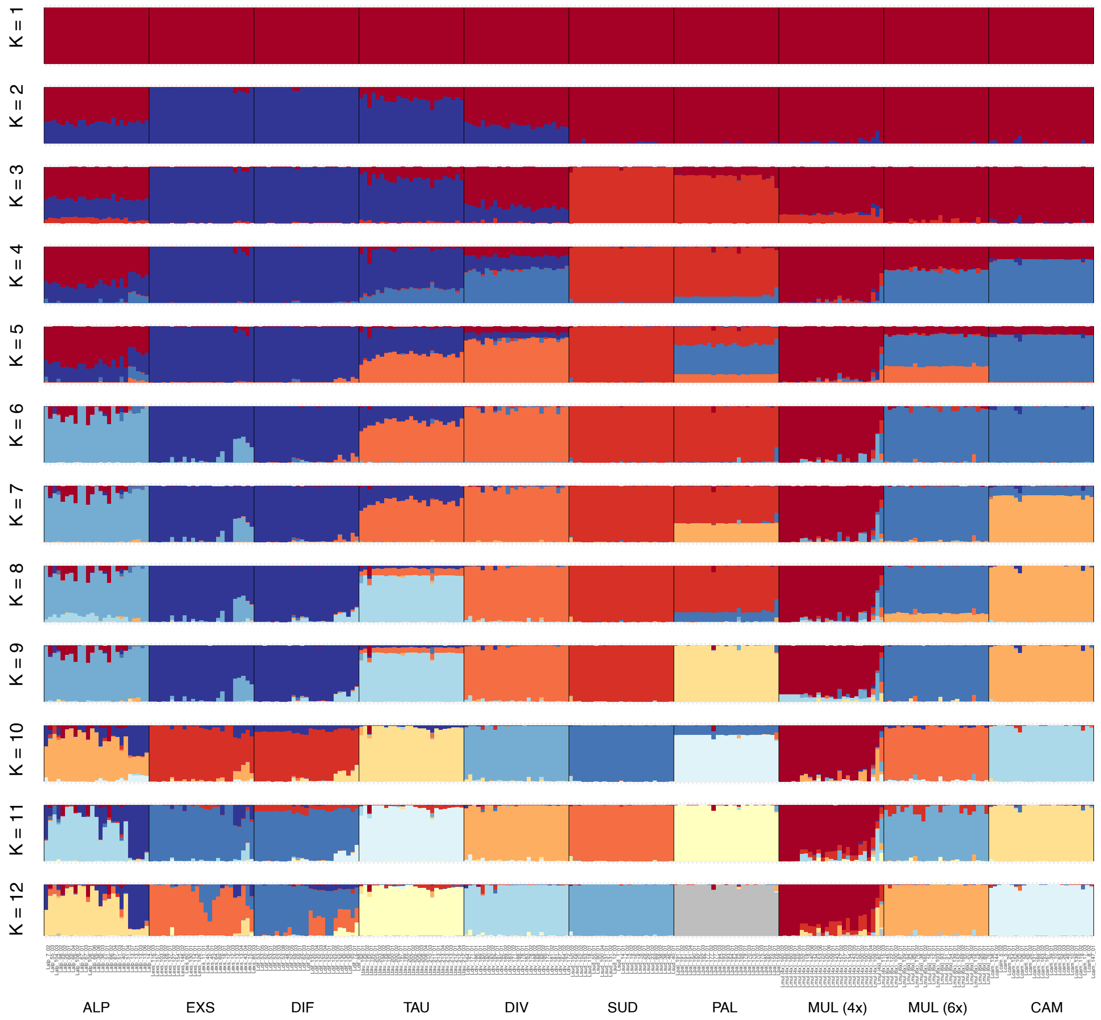

Supplementary Figure S10. STRUCTURE results for a subset of 25 individuals per species of diploid and polyploid species of *Luzula* sect. *Luzula* for K = 1 to 12. Species are indicated below the plot and separated by vertical black lines and their order corresponds to that of clades in the ML tree.

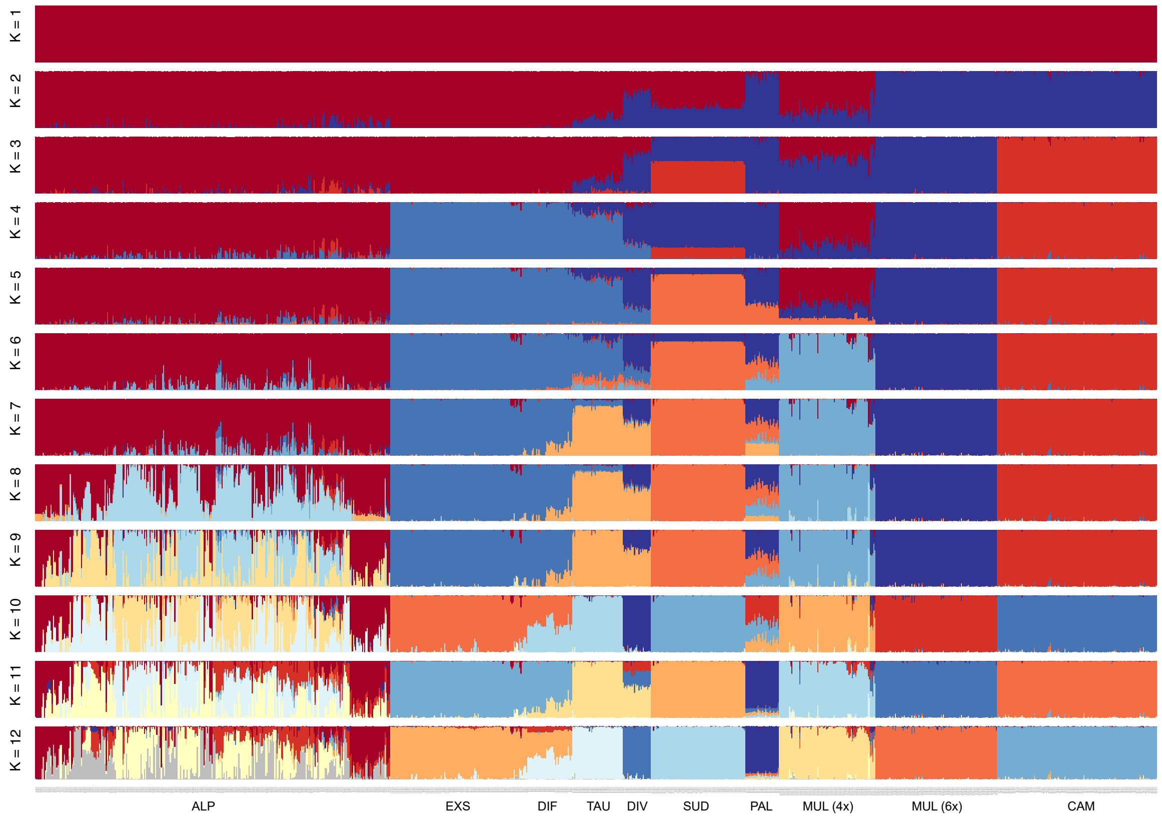

Supplementary Figure S11. STRUCTURE results for diploid and polyploid species of *Luzula* sect. *Luzula* for K = 1 to 12. Species are indicated below the plot and their order corresponds to that of clades in the ML tree (Fig. 4c).

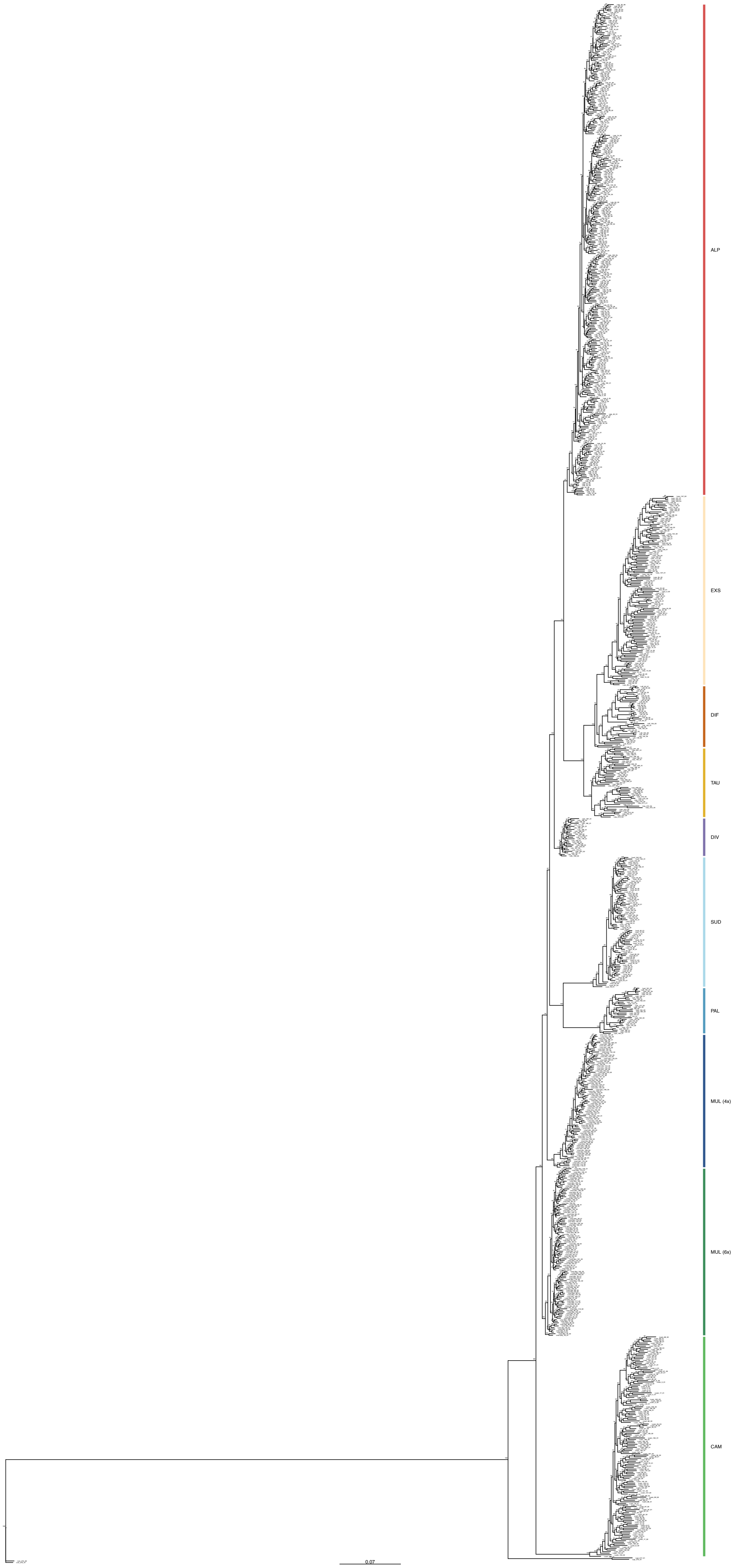

Supplementary Figure S12. Best-scoring maximum likelihood tree of diploid and polyploid species of *Luzula* sect. *Luzula* and outgroup inferred in IQ-TREE 2 based on 9,112 SNPs derived from RADseq. Major clades corresponding to species are indicated on the right. Numbers above branches show bootstrap support values.

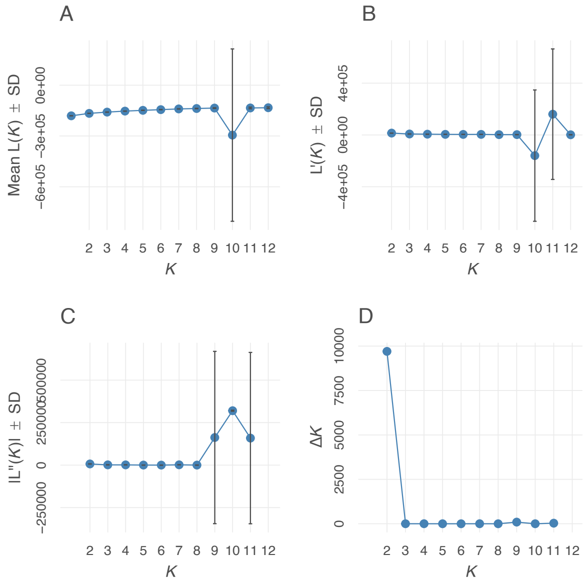

Supplementary Figure S13. Selection of the best fitting K for STRUCTURE run a subset of 25 individuals per species of diploids and polyploids. Change in likelihood (a), as well as first (b) and second (c) derivative and ΔK following Evanno for each K between 1 and 12.

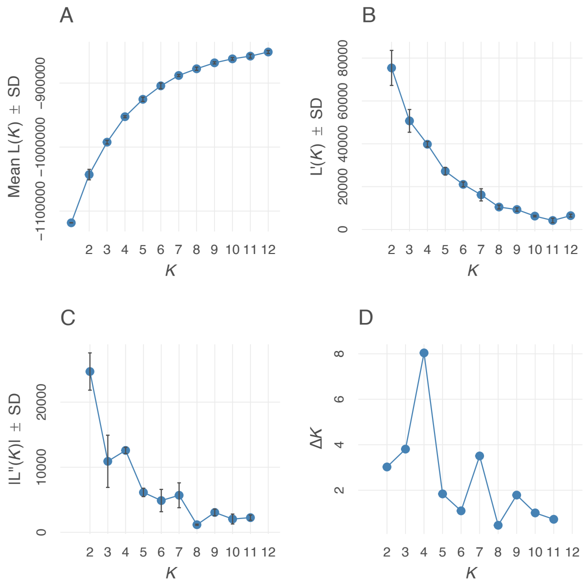

Supplementary Figure S14. Selection of the best fitting K for STRUCTURE run on diploid and polyploid samples. Change in likelihood (a), as well as first (b) and second (c) derivative and ΔK following Evanno for each K between 1 and 12.

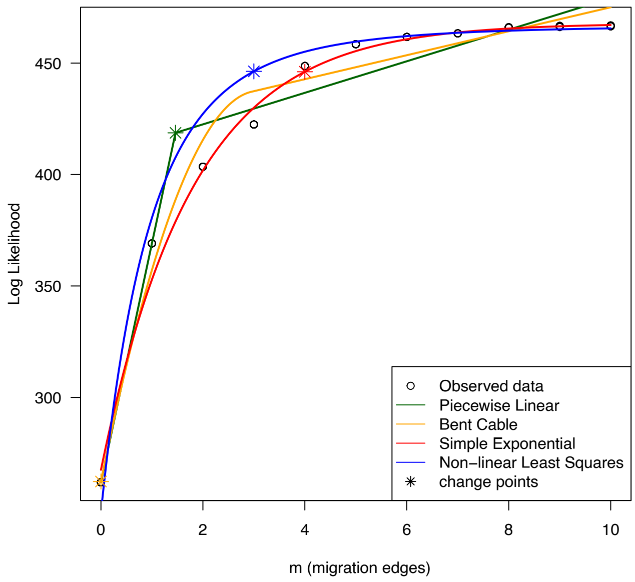

Supplementary Figure S15. Change in Log Likelihood for different numbers of migration edges (*m*) in the TreeMix analysis. The observed data are shown as open circles and different models fitted to the data are represented by colored lines.

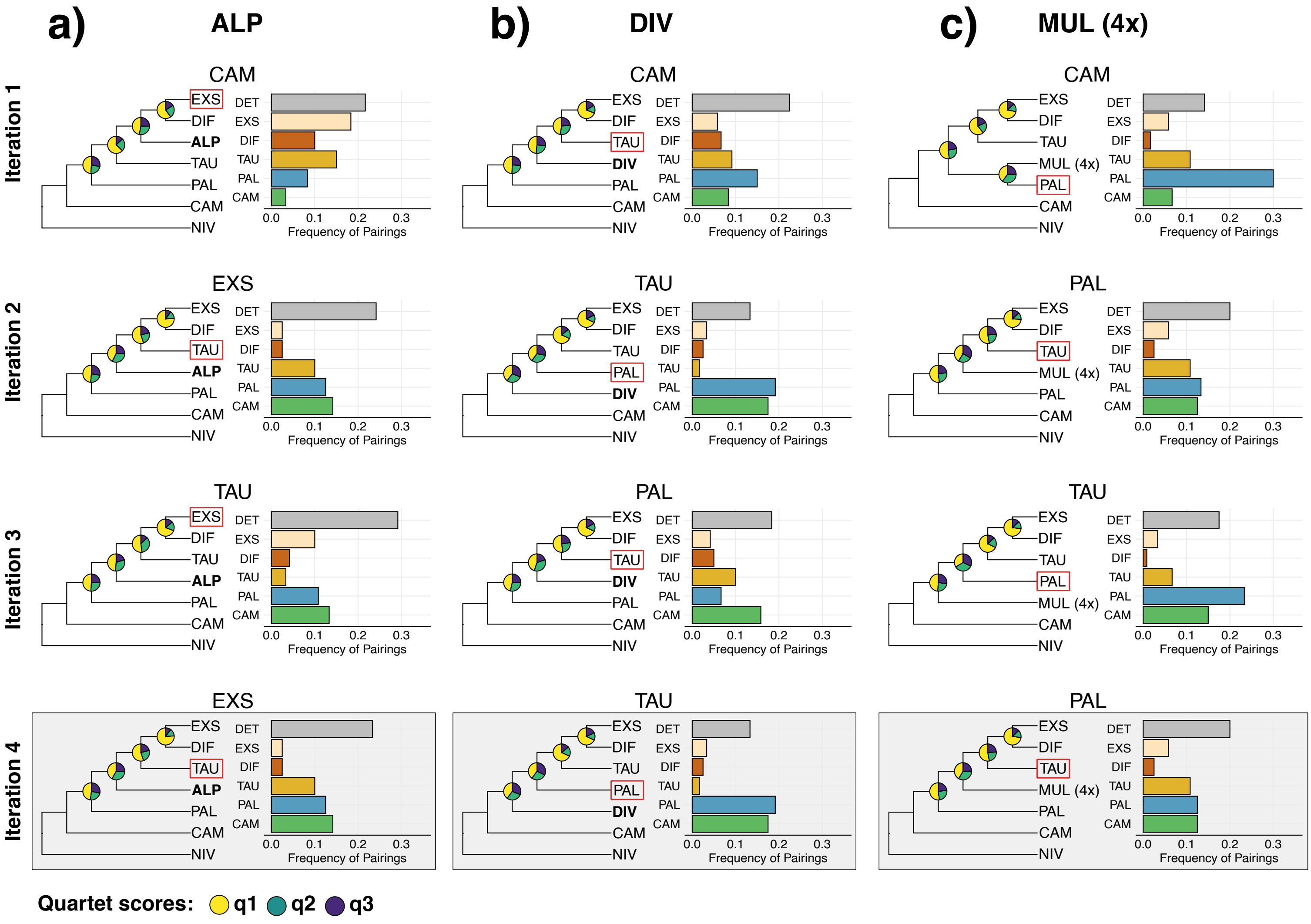

Supplementary Figure S16. Results of iterative genomic polarization of tetraploid a) *Luzula alpina*, b) *L. divulgata* and c) *L. multiflora* (4*x*). Species tree cladograms obtained with ASTRAL based on 120 'locus trees' are presented for four iterations. The species used as reference sequence for polarization is shown at the top and the tetraploid is highlighted in bold. Pie charts indicate quartet support for each branch. Bar plots on the right show pairing frequencies of the polarized tetraploid with other species across the 120 'locus trees' inferred in IQ-TREE2 with colors corresponding to species and grey for the DET clade. The species with the highest pairing within the sister clade of the tetraploid is highlighted with a red box in the cladograms and was chosen as the reference sequence for the next iteration. Grey shadings indicate convergence after the fourth iteration.

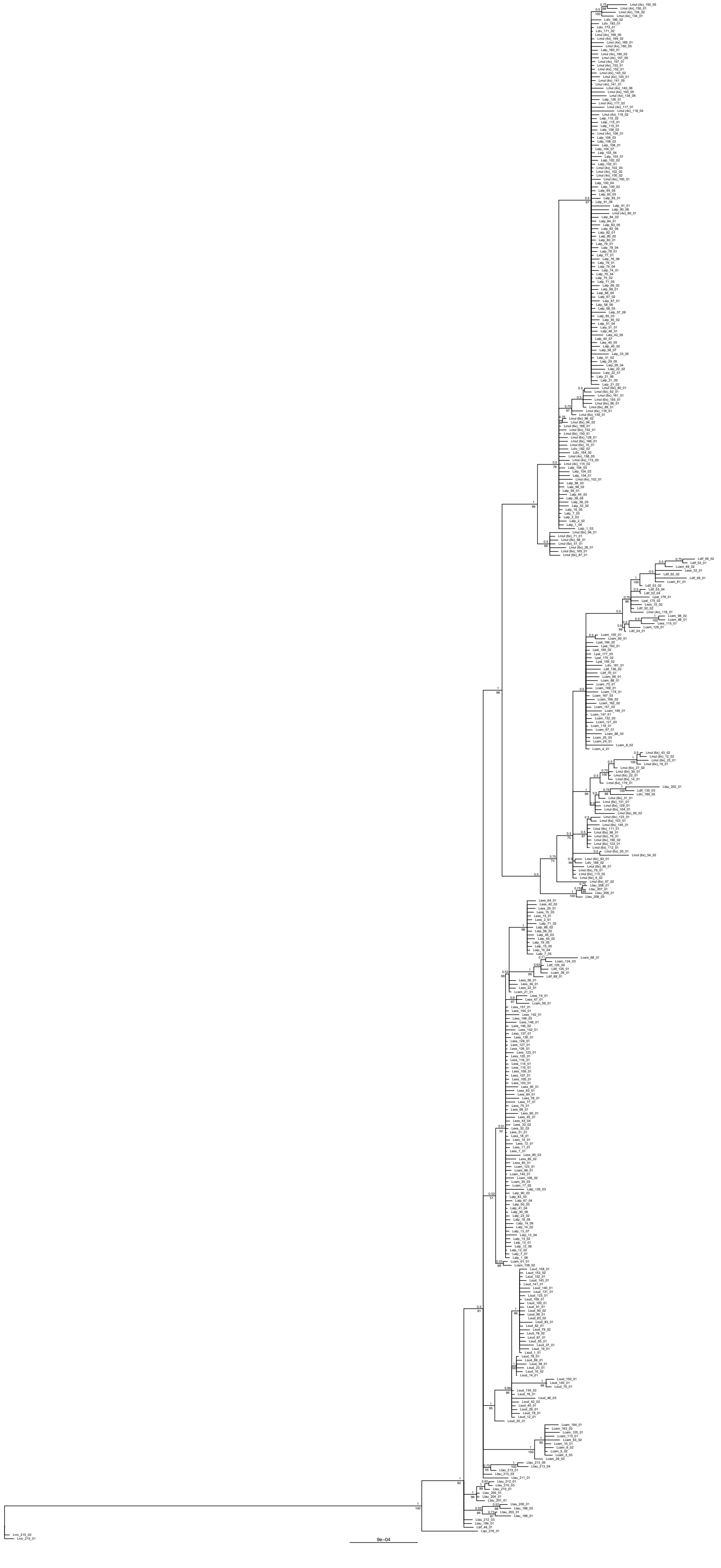

Supplementary Figure S17. Bayesian consensus phylogram inferred from concatenated plastid sequences of diploid and polyploid species of *Luzula* sect. *Luzula* and outgroup. Numbers above branches are posterior probabilities (PP) and those below branches are maximum likelihood bootstrap values (BS) for nodes that were recovered in both Bayesian and ML analyses.

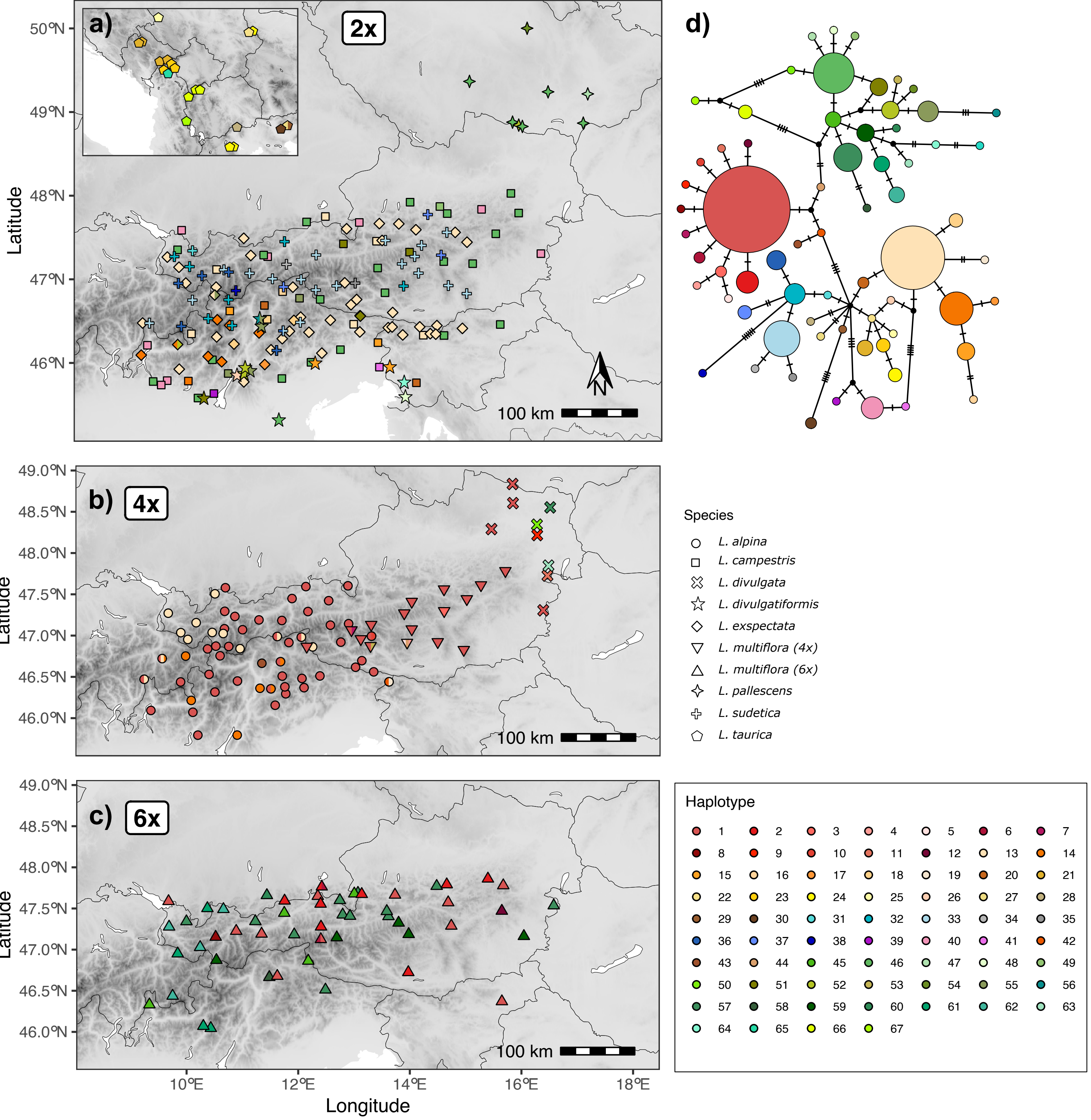

Supplementary Figure S18. Phylogeographic patterns of plastid haplotypes within *Luzula* sect. *Luzula*. Distribution of haplotypes among diploids (a), tetraploids (b) and hexaploids (c). (d) Statistical parsimony network of plastid haplotypes. The size of the circles is proportional to the haplotype's frequency and colors are the same as in (a), (b) and (c). Unsampled haplotypes are shown as black circles.

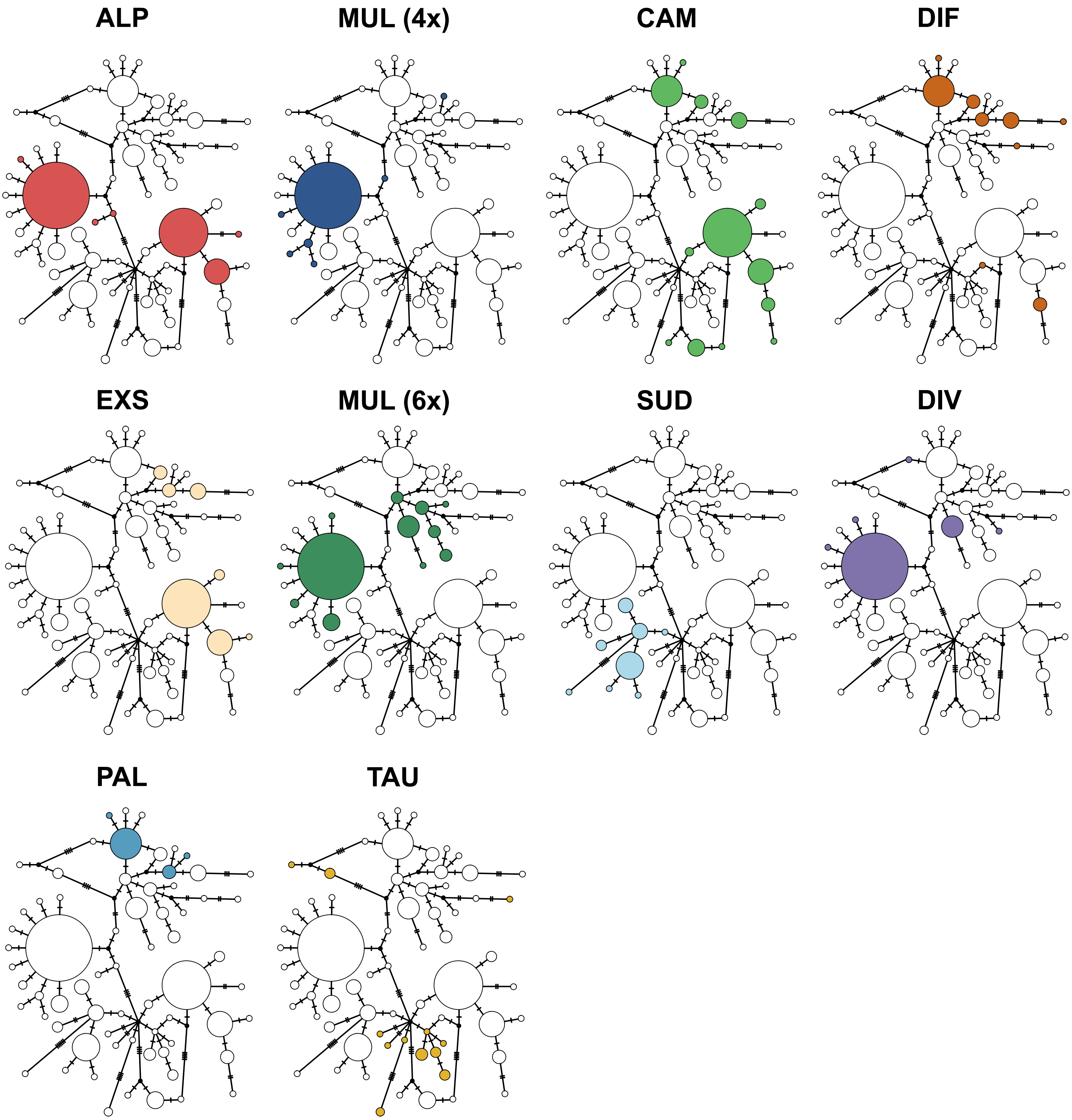

Supplementary Figure S19. Plastid DNA haplotypes for the ten taxa of *Luzula* sect. *Luzula*. For each taxon, sampled haplotypes are indicated by colored circles. The statistical parsimony network is the same as in Supplementary Fig. S18. The size of the circles is proportional to the square-root transformed frequency of the respective haplotype; haplotypes not sampled are shown as small black dots.

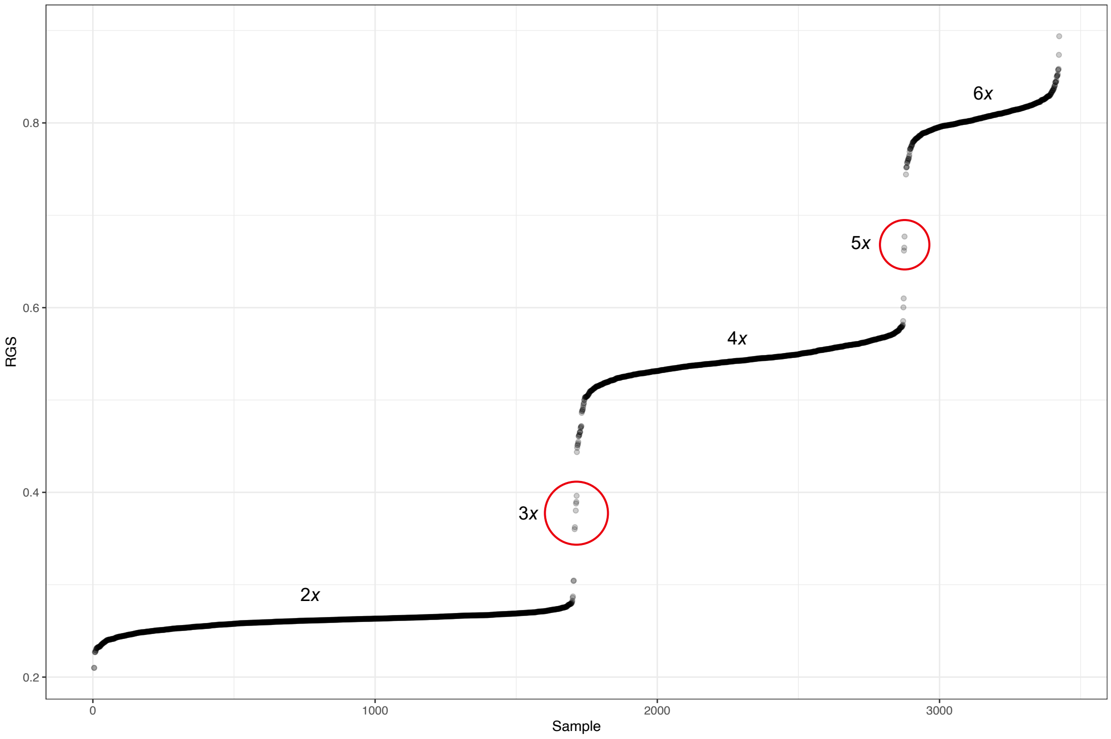

Supplementary Figure S20. Distribution of relative genome size (RGS) in a dataset of 3462 samples of European *Luzula* sect. *Luzula* species. Ploidy levels are indicated above the values and odd ploidies are highlighted with red circles.

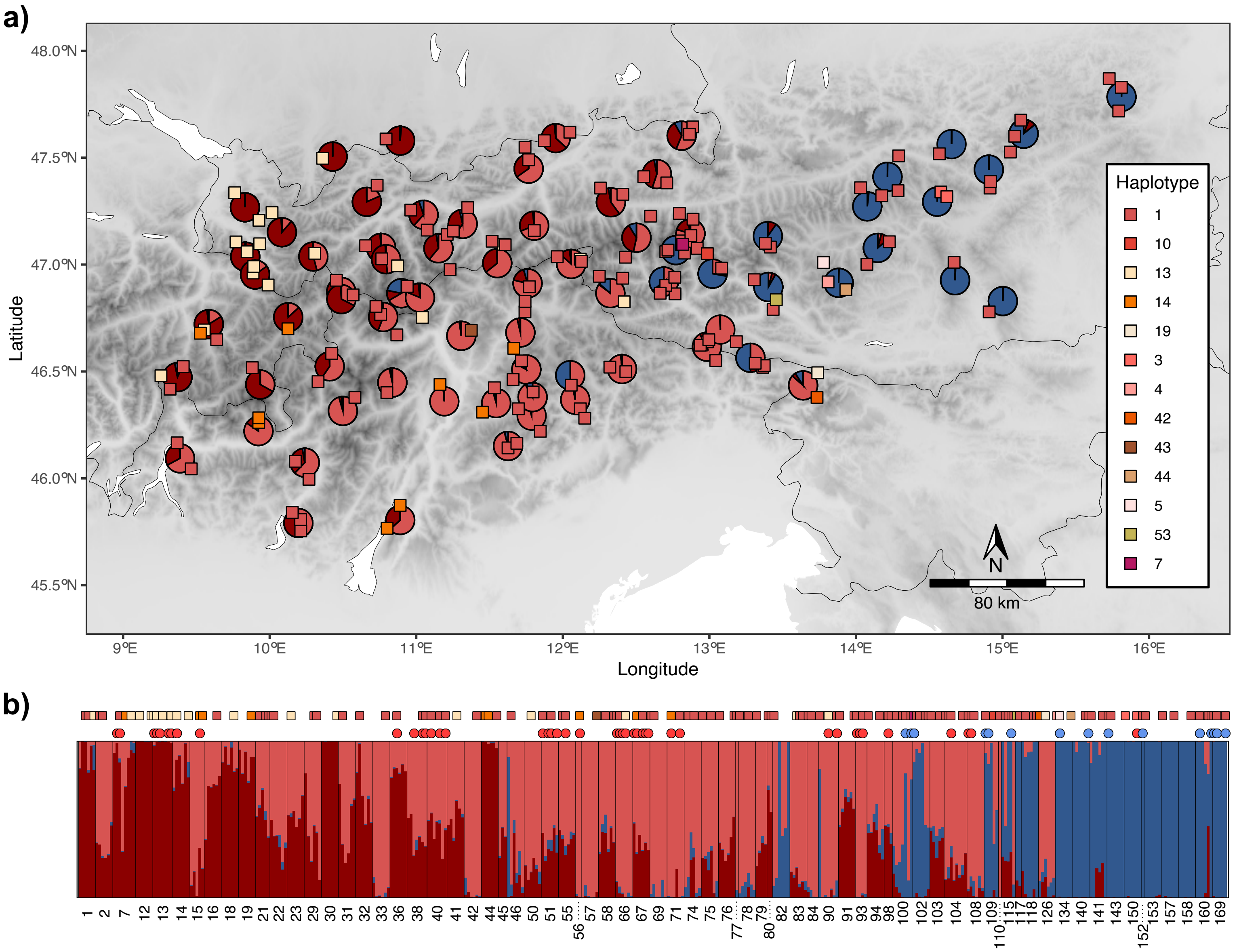

Supplementary Figure S21. Genetic structure within alpine tetraploids and plastid haplotypes. a) Pie charts showing population-averaged assignment of alpine tetraploid *L. alpina* and *L. multiflora* to genetic clusters inferred by STRUCTURE for K = 3, highlighting strong substructure along a south-east cline within *L. alpina.* Squares represent plastid haplotypes as in Supplementary Figs. S18 and S19 and were slightly shifted for better readability. b) STRUCTURE results at K = 3 for each individual grouped by population as indicated by population identifiers below the barcharts. Colored squares at the top indicate haplotypes as in a) and small dots represent chromosome counts for individuals included in this study, either newly produced or from Pungaršek et al. (2023), corresponding to the 12AL+24BL cytotype of *L. alpina* (red) and the 24AL cytotype of *L. multiflora*.
