# Supplementary material for: Hybridization and Polyploidy Shaped the Evolutionary History of a Complex of Cryptic Species in European Woodrushes (Luzula sect. Luzula)
Source: Syst Biol. 2025 Sep 25;75(3):493–516. doi: 10.1093/sysbio/syaf065 (PMC13048012; doi:10.1093/sysbio/syaf065)
Supplement: syaf065_Supplemental_Files [file syaf065_supplemental_files.zip › Supplementary_Results.pdf]

Supplementary Results

Hybridization and Polyploidy shaped the Evolutionary History of a Complex of Cryptic Species in European Woodrushes (*Luzula* sect. *Luzula*)

Valentin Heimer[1,2*], Pau Carnicero[1,3], Carolina Carrizo García[4,5], Andreas Hilpold[2], Jasna Dolenc Koce[6], J. Luis Leal[7], Mingai Li[4], Claudio Varotto[4], Peter Schönswetter[1], Božo Frajman[1]

[1] *Department of Botany, University of Innsbruck, Sternwartestraße 15, 6020 Innsbruck, Austria*

[2] *Institute for Alpine Environment, Eurac Research, Drususallee 1/Viale Druso 1, 39100, Bozen/Bolzano, Italy*

[3] *Department of Animal Biology, Plant Biology and Ecology, Autonomous University of Barcelona, 08193 Bellaterra, Spain.*

[4] *Centro Ricerca e Innovazione, Fondazione Edmund Mach, Via Mach 1, 38098 San Michele all'Adige, Italy*

[5] *Instituto Multidisciplinario de Biología Vegetal (CONICET-UNC), Av. Vélez Sarsfield 1611, 5000 Córdoba, Argentina*

[6] *Department of Biology, Biotechnical Faculty, University of Ljubljana, Jamnikarjeva 101, 1000, Ljubljana, Slovenia*

[7] *Department of Zoology, Stockholm University, Svante Arrheniusväg 18 B, 106 91 Stockholm, Sweden*

[*] Corresponding author

E-mail address: valentin.heimer@uibk.ac.at

Supplementary Results

Genomic Polarization

Genomic polarization of *L. alpina* using *L. campestris* as reference sequence resulted in *L. alpina* being placed sister to a clade containing *L. divulgatiformis* and *L. exspectata* in the species tree estimated by ASTRAL (quartet scores: q1 = 0.47, q2 = 0.28, q3 = 0.25; Fig. 6a). The highest frequency of pairings across locus trees occurred with the entire DET clade (22% of all locus trees) and most pairings with an individual species were with *L. exspectata* within this clade (18%), which was therefore chosen as reference for the second iteration that placed polarized *L. alpina* sister to the DET clade (quartet scores: q1 = 0.43, q2 = 0.33, q3 = 0.25; 24% of pairings). Within this clade, the species to which most pairings occurred was *L. taurica*. Using *L. taurica* as a reference to polarize *L. alpina* again resulted in a sister relationship to the DET clade (q1 = 0.49, q2 = 0.32, q3 = 0.2; 29% of all pairings), with the highest individual pairing frequency occurring with *L. exspectata* (10%), thus indicating convergence of the analysis. Indeed, a fourth iteration using again *L. exspectata* as reference yielded results highly similar to iteration two.

The most pairings across locus trees when analyzing *L. divulgata* with *L. campestris* as a reference occurred with the DET clade (23%), which was reflected in the phylogenetic position of *L. divulgata* recovered by ASTRAL (q1 = 0.49, q2 = 0.25, q3 = 0.27; Fig. 6b). Most pairings occurred with the entire clade; however, the species with the highest number of individual pairings was *L. taurica* (9%), which was therefore selected as reference for the next iteration. This resulted in *L. divulgata* now being placed more basal in the phylogenetic tree and sister to the clade containing *L. pallescens* and the DET clade (q1 = 0.40, q2 = 0.29, q3 = 0.31). The highest individual pairing frequency within that clade was found for *L. pallescens* (19%), which was used as reference for the third iteration, in which *L. divulgata* was again recovered as sister to the DET clade (q1 = 0.45, q2 = 0.35, q3 = 0.20; 18% of pairings). Among those three species, the most frequent pairing occurred with *L. taurica* (10%), once more representing convergence of the analysis, which was confirmed by a fourth iteration with *L. taurica* as reference, resulting in *L. divulgata* again being placed more basally, as in iteration two.

Applying the same approach to tetraploid *L. multiflora* placed this species sister to *L. pallescens* when using *L. campestris* as reference, albeit with relatively low support (q1 = 0.40, q2 = 0.35, q3 = 0.25; 30% of pairings; Fig. 6c). Using *L. pallescens* as reference in the second iteration, *L. multiflora* was sister to the DET clade (q1 = 0.41, q2 = 0.25, q3 = 0.33; 20%) and the most frequent individual pairings occurred with *L. taurica* (11%), which was therefore chosen as reference for iteration three. Here, *L. multiflora* was sister to a clade comprising the DET clade and *L. pallescens* (q1 = 0.49, q2 = 0.24, q3 = 0.27), with most individual pairings with the latter (23%). A fourth iteration using *L. pallescens* as reference yielded results very similar to iteration two, indicating convergence.
